# Investigating synthetic lethality and PARP inhibitor resistance in pancreatic cancer through enantiomer differential activity

**DOI:** 10.1038/s41420-025-02382-3

**Published:** 2025-03-16

**Authors:** Mirco Masi, Laura Poppi, Viola Previtali, Shannon R. Nelson, Kieran Wynne, Giulia Varignani, Federico Falchi, Marina Veronesi, Ennio Albanesi, Daniele Tedesco, Francesca De Franco, Andrea Ciamarone, Samuel H. Myers, Jose Antonio Ortega, Greta Bagnolini, Giovanni Ferrandi, Fulvia Farabegoli, Nicola Tirelli, Giuseppina Di Stefano, Giorgio Oliviero, Naomi Walsh, Marinella Roberti, Stefania Girotto, Andrea Cavalli

**Affiliations:** 1 Computational and Chemical Biology, Italian Institute of Technology IIT, 16163 Genoa, Italy; 2 Department of Pharmacy and Biotechnology, University of Bologna, 40126 Bologna, Italy; 3 National Institute for Cellular Biotechnology, School of Biotechnology, Dublin City University, D09 NR58 Dublin, Ireland; 4 Systems Biology Ireland, School of Medicine, University College Dublin, D04 V1W8 Dublin, Ireland; 5 Conway Institute of Biomolecular & Biomedical Research, University College Dublin, D04 V1W8 Dublin, Ireland; 6 Structural Biophysics Facility, Italian Institute of Technology IIT, 16163 Genoa, Italy; 7 Department of Neuroscience and Brain Technologies, Neurofacility, Italian Institute of Technology IIT, 16163 Genoa, Italy; 8 Institute for Organic Synthesis and Photoreactivity (ISOF), National Research Council of Italy (CNR), I-40129 Bologna, Italy; 9 TES Pharma S.r.l., I-06073 Perugia, Italy; 10 Laboratory for Polymers and Biomaterials, Italian Institute of Technology IIT, 16163 Genoa, Italy; 11 Department of Surgical and Medical Sciences, University of Bologna, 40126 Bologna, Italy; 12 Centre Européen de Calcul Atomique et Moléculaire (CECAM), Ecole Polytechnique Fédérale de Lausanne, 1015 Lausanne, Switzerland

**Keywords:** Small molecules, Pharmacology, Cell death

## Abstract

The RAD51-BRCA2 interaction is central to DNA repair through homologous recombination. Emerging evidence indicates RAD51 overexpression and its correlation with chemoresistance in various cancers, suggesting RAD51-BRCA2 inhibition as a compelling avenue for intervention. We previously showed that combining olaparib (a PARP inhibitor (PARPi)) with *RS*-35d (a BRCA2-RAD51 inhibitor) was efficient in killing pancreatic ductal adenocarcinoma (PDAC) cells. However, *RS*-35d impaired cell viability even when administered alone, suggesting potential off-target effects. Here, through multiple, integrated orthogonal biological approaches in different 2D and 3D PDAC cultures, we characterised *RS*-35d enantiomers, in terms of mode of action and single contributions. By differentially inhibiting both RAD51-BRCA2 interaction and sensor kinases ATM, ATR and DNA-PK, *RS*-35d enantiomers exhibit a ‘within-pathway synthetic lethality’ profile. To the best of our knowledge, this is the first reported proof-of-concept single small molecule capable of demonstrating this built-in synergism. In addition, *RS*-35d effect on *BRCA2*-mutated, olaparib-resistant PDAC cells suggests that this compound may be effective as an anticancer agent possibly capable of overcoming PARPi resistance. Our results demonstrate the potential of synthetic lethality, with its diversified applications, to propose new and concrete opportunities to effectively kill cancer cells while limiting side effects and potentially overcoming emerging drug resistance.

## Introduction

Synthetic lethality arises when the impairment of a single gene does not affect cell viability, while the simultaneous dysfunction of two genes results in cell death [1]. This paradigm has prompted the development of novel therapeutic strategies that exploit vulnerabilities arising from genetic alterations, such as mutations in DNA repair genes [2, 3]. DNA damage response (DDR) pathways safeguard genome integrity and have been proved to be suitable targets for synthetic lethality-based antitumoral strategies [4]. Dysregulation of one or more DDR pathways can lead to the accumulation of DNA lesions and genomic instability, hallmarks of many cancers [5]. When defects in a specific DDR pathway arise, cancer cells become over-reliant on other pathways for survival and targeting these DDR alternative mechanisms can lead to selective tumour cell death through synthetic lethality [2]. This strategy is exemplified by the clinical application of poly(ADP-ribose) polymerase (PARP) inhibitors (PARPi) in oncologic patients harbouring *BRCA1/2* mutations. While PARP is required for DNA single-strand breaks (SSBs) repair, BRCA1 and BRCA2 participate in DNA double-strand breaks (DSBs) repair by homologous recombination (HR). PARP inhibition upon *BRCA1/2* mutations-induced HR impairment results in cell cycle arrest, apoptosis and consequent cell death through synthetic lethality [2]. Among the FDA-approved PARPi, olaparib was the first to receive approval as first-line maintenance treatment for several BRCA-mutated cancers, including ovarian, breast, prostate and pancreatic cancer [2], which is still one of the major unmet oncological need [6]. Central to HR is the intricate interplay between BRCA2 and the recombinase RAD51, which allows an accurate DSBs repair [7]. RAD51-BRCA2 protein-protein interaction (PPI) relies on eight well-conserved BRC repeats, with BRC4 being crucial for RAD51 multimerization and activity [8]. BRC4 binds RAD51 in two hydrophobic pockets, zone I and zone II. Zone I is pivotal for RAD51 multimerization and accommodates BRC4 FxxA motif (BRCA2 residues 1524–1527), while zone II is more evolutionarily conserved and fits BRC4 LFDE motif (BRCA2 residues 1545–1548) [8–11]. We demonstrated that the BRC4 peptide-induced RAD51-BRCA2 disruption resulted in HR inhibition, sensitisation to anticancer drugs [12] and caused specific proteomic alterations within DDR pathways [13]. We also showed that RAD51-BRCA2 chemical inhibition potentiated PARPi-induced cell death [14–16]. Moreover, a wide range of cancers show elevated RAD51 expression and RAD51-mediated HR rate [17], which correlate with reduced overall survival [18] and resistance to DNA damage-inducing chemotherapy and radiotherapy [19]. Therefore, chemical inhibition of RAD51-BRCA2 interaction to mimic the BRCA2 mutation phenotype (i.e. BRCAness) presents a compelling avenue for intervention, as it could be applied in a fully small molecule-induced synthetic lethality anticancer strategy for difficult-to-treat tumours, including PARPi-resistant and BRCA-proficient pancreatic ductal adenocarcinoma (PDAC). We previously described the dihydroquinolone pyrazoline-based RAD51-BRCA2 inhibitor *RS*-35d, designed to target the LFDE motif of RAD51 zone II [14]. Despite the medicinal chemistry campaign undertaken to improve *RS*-35d solubility and target potency, *RS*-35d was the only compound to cause a strong HR inhibition and to significantly affect cell viability (patent: WO2021116999A1). Nevertheless, *RS*-35d induced cell death even without olaparib coadministration, hinting at a possible off-target activity. As *RS*-35d is a racemic mixture, we deemed it crucial to elucidate its mode of action by characterising the individual contributions of its two enantiomers. This approach aimed to determine whether the differential biological profiles of the enantiomers could account for the off-target effects observed in the racemic mixture. In the present study, through the integration of orthogonal biological approaches in different 2D and 3D PDAC cultures, we successfully demonstrated that the purported off-target *RS*-35d cytotoxicity is actually the realisation/manifestation of an intrinsic synthetic lethal profile that falls within the paradigm of ‘within-pathway synthetic lethality’ [20]. This serendipitous discovery may pave the way for new therapeutic strategies exploiting a novel and previously unexplored chemically-induced synthetic lethality anticancer framework.

## Results

### *RS*-35d enantiomers' differential inhibition of RAD51-BRCA2 interaction, induction of HR impairments and DNA damaging effects

**Fig. 1:**
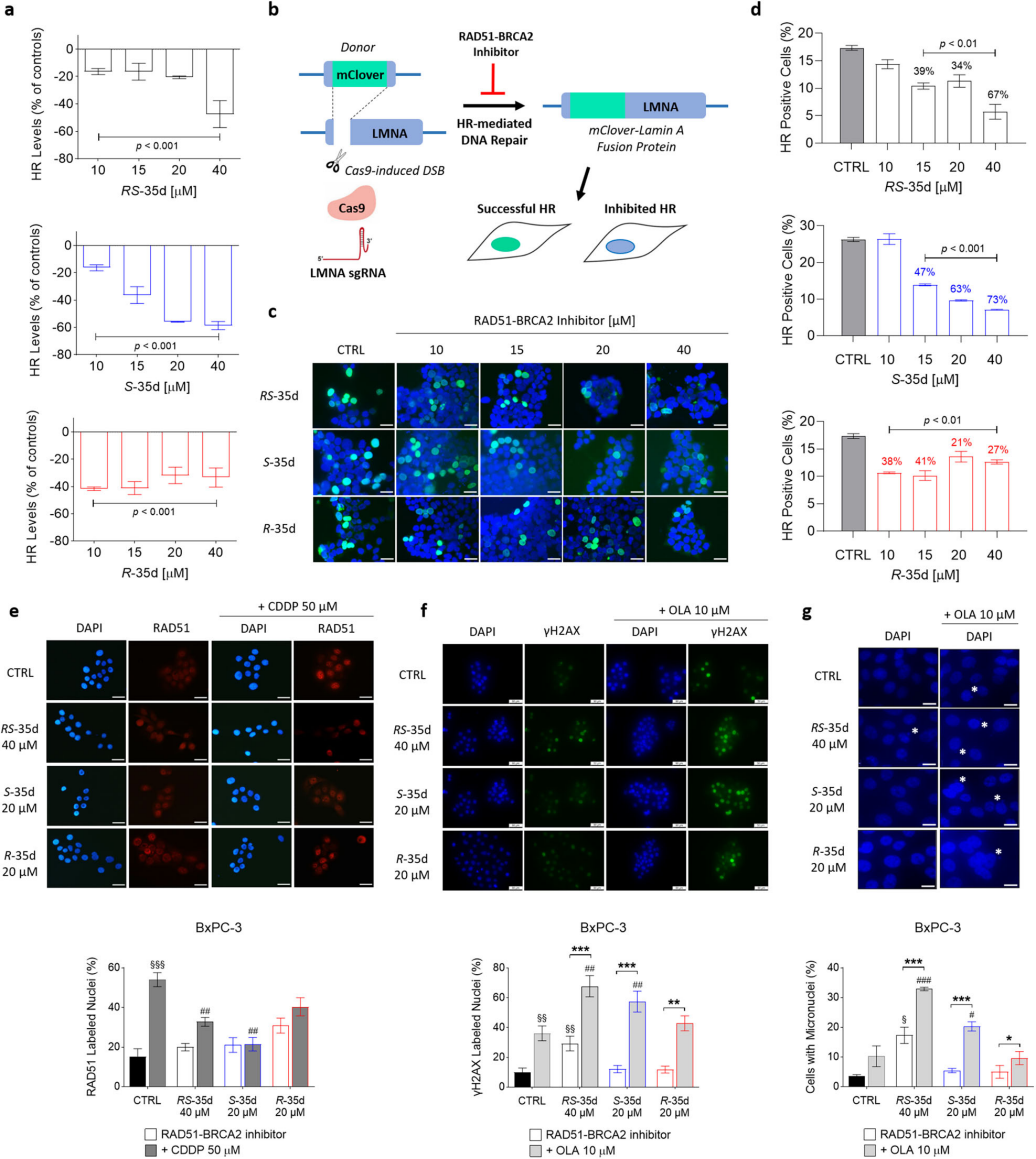
*RS*-35d, *S*-35d and *R*-35d differential effects on HR, RAD51 nuclear activity and DNA damage. **a** Effect on HR caused by *RS*-35d, *S*-35d and *R*-35d in BxPC-3 cells. Results are expressed as mean ± SD (n = 3). **b** mClover-Lamin assay for DNA repair by HR performed in HEK293 cells transfected with Lamin A sgRNA and mClover-Lamin A donor and treated with *RS*-35d, *S*-35d and *R*-35d. **c** Representative microscopic merged images of mClover-Lamin A fusion protein and DNA (DAPI staining) fluorescence after exposure to increasing doses of *RS*-35d, *S*-35d or *R*-35d (scale bar, 30 μm). **d** Analysis of HR-positive (mClover-Lamin A-positive) cells after administration of increasing doses of *RS*-35d, *S*-35d or *R*-35d (the three compounds were tested in independent experiments and their effects were compared to their respective vehicle control). Results are expressed as mean ± SD (n = 3). Statistical analysis was performed with one-way ANOVA followed by Dunnett’s multiple comparison test, with *p* < 0.01 or *p* < 0.001 vs CTRL. **e** Immunofluorescence detection of nuclear RAD51 in BxPC-3 exposed to 40 µM *RS*-35d, 20 µM *S*-35d or 20 µM *R*-35d alone or after pre-treatment with 50 µM cisplatin (CDDP). Representative images of DAPI-stained nuclei and the corresponding nuclear localisation of RAD51 immune-labelling (scale bar, 30 μm) and analysis of RAD51-positive nuclei (%). Results are expressed as mean ± SD (n = 3). Statistical analysis was performed with one-way ANOVA followed by Tuckey’s multiple comparison test, with ^§§§^*p* < 0.001 vs CTRL and ^##^*p* < 0.01 vs 50 µM CDDP. **f** Evaluation of DNA damage via nuclear γH2AX foci immune detection in BxPC-3 cells treated for 48 h with 40 µM *RS*-35d, 20 µM *S*-35d or 20 µM *R*-35d alone or in combination with 10 µM olaparib (OLA). Representative images of DAPI-stained nuclei and the corresponding γH2AX immune-labelling (scale bar, 50 μm) and analysis of γH2AX-positive nuclei (%). **g** Micronuclei evaluation in BxPC-3 cells treated for 72 h with 40 µM *RS*-35d, 20 µM *S*-35d or 20 µM *R*-35d alone or in combination with 10 µM OLA. Representative images of DAPI-stained nuclei (white asterisks indicate micronuclei presence) (scale bar, 15 μm) and analysis of micronuclei-bearing cells (%). Results are expressed as mean ± SD (n = 3). Statistical analysis was performed with one-way ANOVA followed by Tuckey’s multiple comparison test, with ^§^*p* < 0.05 or ^§§^*p* < 0.01 vs CTRL; ^#^*p* < 0.05, ^##^*p* < 0.01 or ^###^*p* < 0.001 vs 10 µM OLA; *p < 0.05, **p < 0.01 or ***p < 0.001 vs RAD51-BRCA2 inhibitor alone.

*RS*-35d enantiomers *S*-35d and *R*-35d were separated, assigned their absolute configuration and investigated for their ability to bind RAD51 and inhibit its interaction with BRCA2 by means of a competitive ELISA assay, microscale thermophoresis (MST) and molecular docking calculations (Supplementary Figs. S1–S4, Supplementary Tables S1 and S2). Obtained data indicate a different RAD51-BRCA2 inhibitory activity for the two enantiomers, with *S*-35d as a better RAD51-BRCA2 inhibitor compared to *R*-35d. Upon investigation of a possible differential effect on HR repair in *BRCA2*-proficient PDAC cells BxPC-3, *RS*-35d induced a 50% HR inhibition at 40 µM, while a reduction of 55% and 58% in HR efficiency was observed for *S*-35d at 20 and 40 µM doses respectively (Fig. 1a), consistent with MST data. Conversely, *R*-35d produced a limited dose-independent HR inhibition, with an average 35% inhibition at all tested concentrations (Fig. 1a). To better examine if RAD51 inhibition induced by *RS*-35d, *S*-35d or *R*-35d affected HR-mediated DNA repair, we employed the mClover-Lamin A assay [21]. The repair of a Cas9 nuclease-induced DNA break in Lamin A gene through HR but no other repair mechanisms can lead to the reconstitution of a mClover-Lamin A fluorescent fusion protein localised to the nuclear membrane (Fig. 1b). HR-mediated repair efficiency was measured by counting cells positive for the fusion protein (i.e. that exhibit a nuclear-limited green fluorescence) after treatment with *RS*-35d, *S*-35d and *R*-35d (Fig. 1c). Consistently with data shown in Fig. 1a, R*S*-35d treatment resulted in a 67% HR inhibition only at 40 µM, *S*-35d induced a significant (>50%) HR inhibition at both 20 µM (63%) and 40 µM (73%), while *R*-35d treatment produced a dose-independent, limited HR inhibition (Fig. 1d). These data further suggest that *S*-35d higher binding affinity for RAD51 correlates with its higher inhibitory effect on HR repair, while *R*-35d weaker binding to RAD51 does not strongly impair HR-directed DNA repair. To confirm the correlation between RAD51 inhibition and compromised HR, we analysed RAD51 nuclear localisation after cisplatin (CDDP)-induced DNA damage in BxPC-3 cells. RAD51 nuclear foci were significantly reduced by 40 µM *RS*-35d treatment and the same trend was observed for 20 µM *S*-35d, but not for 20 µM *R*-35d, which appeared ineffective (Fig. 1e). These results are in line with MST data indicating *S*-35d stronger RAD51 binding compared to *R*-35d, as well as for *S*-35d and *R*-35d HR disrupting effect. A prolonged HR inhibition results in increased DNA damage, leading to the acquisition of mutations and genomic instability [5]. These effects are exacerbated and amplified when DNA repair mechanisms for SSBs are inhibited through PARP inhibition. Therefore, the extent of DNA damage and genomic instability produced by administration of *RS*-35d, *S*-35d or *R*-35d alone or in combination with 10 µM olaparib [14–16]—chosen to mimic olaparib plasma concentrations (C_min_ = 1 μM; C_max_ = 8.5 μM) in adult pancreatic cancer patients receiving olaparib 100 mg BID in combination with other drugs (e.g. gemcitabine 600 mg/m^2^) [22, 23]—to BxPC-3 cells was assessed by nuclear γH2AX foci detection and micronuclei evaluation. 40 µM *RS*-35d significantly increased γH2AX nuclear labelling and micronuclei formation, further enhanced by olaparib coadministration (Fig. 1f, g). Noteworthily, treatment with either 20 µM *S*-35d or *R*-35d alone appeared ineffective, while only *S*-35d/olaparib coadministration enhanced γH2AX foci and micronuclei formation (Fig. 1f, g). These data are consistent with *S*-35d stronger binding to RAD51 and HR disrupting effects compared to *R*-35d. These results support a differential inhibitory effect of *S*-35d and *R*-35d on RAD51 activity/HR repair and suggest that the two enantiomers may induce synthetic lethality to a different extent.

### *S*-35d and *R*-35d differential synergism with olaparib and synthetic lethality profile in PDAC cultures

**Fig. 2:**
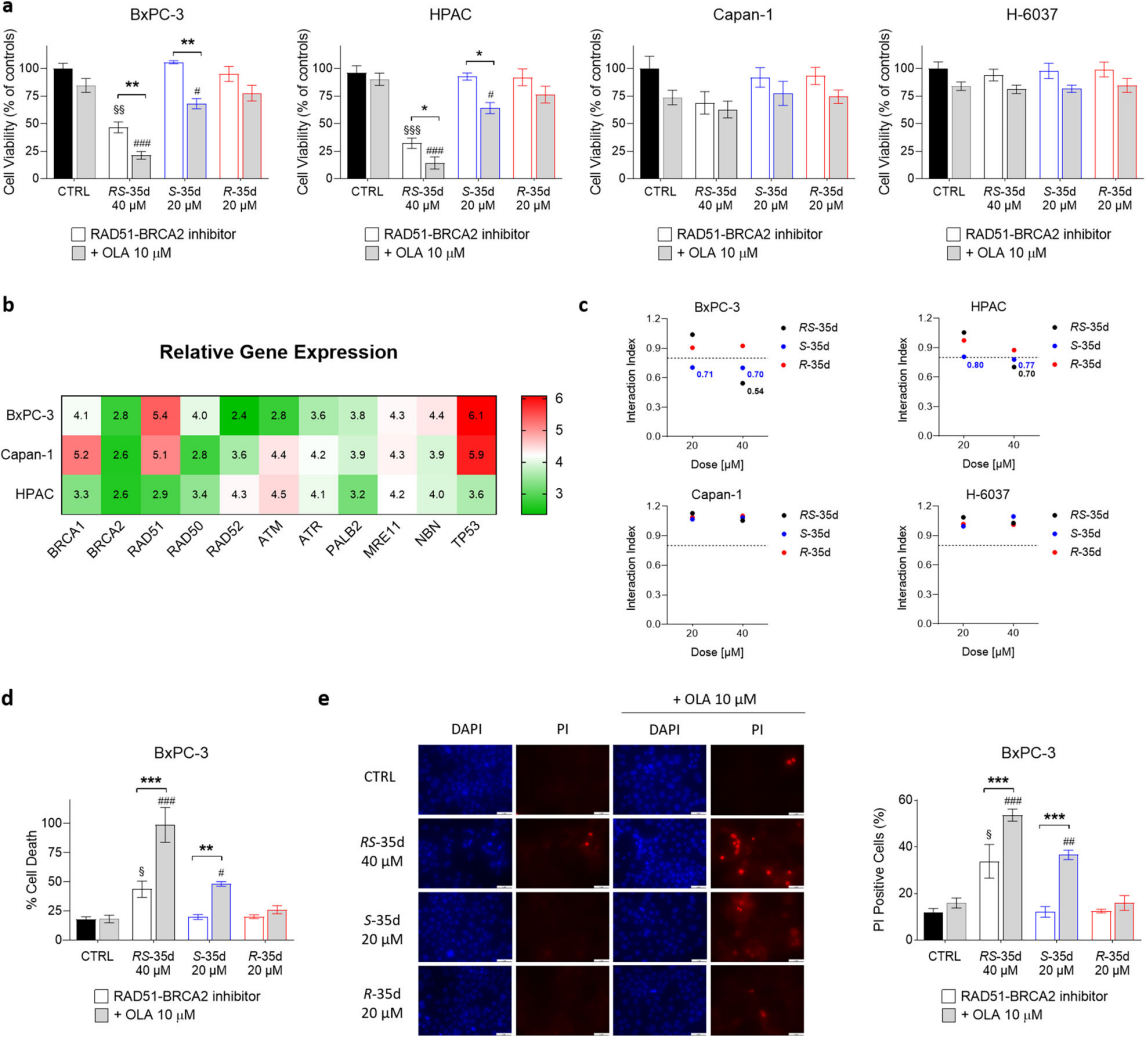
*RS*-35d, *S*-35d and *R*-35d differential synergism with olaparib and their diverse impact on cell viability. **a** Cell viability measured after 72 h exposure to 40 µM *RS*-35d, 20 µM *S*-35d or 20 µM *R*-35d alone or in combination with 10 µM olaparib (OLA) in BxPC-3, HPAC, Capan-1 and H-6037 cells. **b** Gene expression profile of the main HR genes in the employed three PDAC cell lines. Relative gene expression values were obtained from the Cancer Dependency Map portal Depmap (https://depmap.org/portal/) and are inferred from RNA-seq data using the RSEM tool and are reported after log_2_ transformation, using a pseudo-count of 1; log_2_(TPM + 1). **c** Interaction index (i. index) of *RS*-35d, *S*-35d or *R*-35d association with olaparib in BxPC-3, HPAC, Capan-1 and H-6037 cell lines. I. index values were made explicit in their respective graphs when the drug association showed synergism (i. index < 0.8). Cell death assessment by CellTox Green (**d**) and vital dyes staining (**e**) measured after 72 h exposure to 40 µM *RS*-35d, 20 µM *S*-35d or 20 µM *R*-35d alone or in combination with 10 µM OLA in BxPC-3 cells; **d** *RS*-35d, *S*-35d or *R*-35d alone or in combination with 10 µM OLA in BxPC-3 cells; **e** Representative images of DAPI-stained nuclei and the corresponding PI-stained cells (scale bar, 50 μm) and analysis of PI-positive cells (%). Results are expressed as mean ± SD (n = 3). Statistical analysis was performed with two-way ANOVA followed by Tuckey’s multiple comparison test, with ^§^*p* < 0.05, ^§§^*p* < 0.01 or ^§§§^*p* < 0.001 vs CTRL; ^#^*p* < 0.05, ^##^*p* < 0.01 or ^###^*p* < 0.001 vs 10 µM OLA; *p < 0.05, **p < 0.01 or ***p < 0.001 vs RAD51-BRCA2 inhibitor alone.

To elucidate a possible *S*-35d and *R*-35d differential impact on cell viability, we evaluated their synthetic lethality profile when combined with olaparib in multiple PDAC cell lines (Fig. 2, Supplementary Fig. S5). BxPC-3 and HPAC are primary tumour-derived cell lines displaying different expression for multiple relevant genes for HR pathway. In particular, BxPC-3 cells express high RAD51 levels and mutated TP53, while HPAC cells display lower RAD51 levels and wild-type TP53 [24]. Capan-1 are a liver metastasis PDAC line harbouring a somatic *BRCA2* mutation and H-6037 are non-cancerous primary pancreatic epithelial cells. In agreement with DNA damage data, 40 µM *RS*-35d alone significantly reduced BxPC-3 cells viability, further enhanced by olaparib coadministration (Fig. 2a). Conversely, both *S*-35d and *R*-35d alone could not affect cell viability, while only *S*-35d reduced cell viability, at both 20 and 40 µM, upon coadministration with olaparib (Fig. 2a). These data correlate with *S*-35d stronger RAD51 binding and HR-impairing effect compared to *R*-35d, suggesting that the two enantiomers have a different synthetic lethality profile when combined with a PARPi. To note, despite producing a dose-dependent effect not observed in BxPC-3 cells (Supplementary Fig. S5), similar results were obtained on HPAC cells, although exposure to 40 µM *RS*-35d had a higher impact on cell viability compared to BxPC-3 cells (Fig. 2a), suggesting HPAC cells higher sensitivity to *RS*-35d effect potentially correlated to the different TP53 status and HR pathway genes expression (Fig. 2b) [24]. Conversely, *RS*-35d, *S*-35d and *R*-35d alone or combined with olaparib did not affect cell viability of *BRCA2*-mutated, HR-defective Capan-1 cells and non-neoplastic, pancreatic H-6037 cells (Fig. 2a), confirming that the resulting antiproliferative effect observed in BxPC-3 cells was a consequence of the impairment of RAD51-BRCA2 mechanism and synergism with olaparib. Interaction index (i. index) of *RS*-35d, *S*-35d or *R*-35d with olaparib indicated a synergistic effect of 40 µM *RS*-35d/10 µM olaparib, 20 µM *S*-35d/10 µM olaparib and 40 µM *S*-35d/10 µM olaparib in BxPC-3 and, to a lesser extent, in HPAC cells, while for Capan-1 and H-6037 only an additive effect was reported (Fig. 2c). These results are in line with literature data indicating an overdependency of cancer cells on DDR pathways and their consequent up-regulation [25]. Highly proliferating cancer cells like BxPC-3 and HPAC greatly rely on BRCA2-dependent HR repair [25], where RAD51-BRCA2/PARP inhibition-based synthetic lethality strategy is effective in eradicating tumour cells, although its impact and outcome may be influenced by TP53 status and DDR machinery regulation and expression (Fig. 2b) [26]. Conversely, *BRCA2*-mutated/HR-defective Capan-1 cells do not operate RAD51-BRCA2-dependent HR [27] and the rationale here employed cannot significantly affect cell viability. In addition to a slower replication rate compared to tumour cells, non-cancerous cells like H-6037 display a full complement of DNA repair pathways, thus compensating for the chemically-induced loss of individual DDR pathways [28] and mitigating RAD51-BRCA inhibitor/olaparib impact on cell viability. The simultaneous inhibition of PARP and RAD51-BRCA2-dependent HR should result in BxPC-3 cell death as a consequence of a progressive DNA damage accumulation. To confirm the synthetic lethality strategy applied in cell cultures, we assessed cell death, the correlated cell morphology and reaction to vital dyes 4′,6-diamidino-2-phenylindole (DAPI) and propidium iodide (PI) in BxPC-3 cells. While healthy cells display a normal nuclear morphology and absence of PI staining, apoptotic cells show increased DAPI staining (due to an increased nuclear condensation) and PI staining (due to compromised membrane integrity) [29]. In line with cell viability data, we observed a statistically significant cell death produced by 40 µM *RS*-35d, markedly enhanced by olaparib coadministration (Fig. 2d, e). Conversely, the two enantiomers alone could not produce any significant effect on BxPC-3 cells and only *S*-35d/olaparib association induced a marked increase of cell death, while *R*-35d/olaparib coadministration was ineffective (Fig. 2d, e). Altogether, these data clearly indicate that the stronger RAD51 binding and inhibition exerted by *S*-35d results in a synergistic effect when administered with a PARPi, showing a fully chemical-induced synthetic lethality pattern which cannot be observed for *R*-35d. However, in terms of therapeutic strategy and enantiomers individual contribution, a further characterisation is warranted to better dissect their relevance within cancer cells molecular complexity.

### *S*-35d and *R*-35d differential impact on cancer hallmarks of tumour aggressiveness

**Fig. 3:**
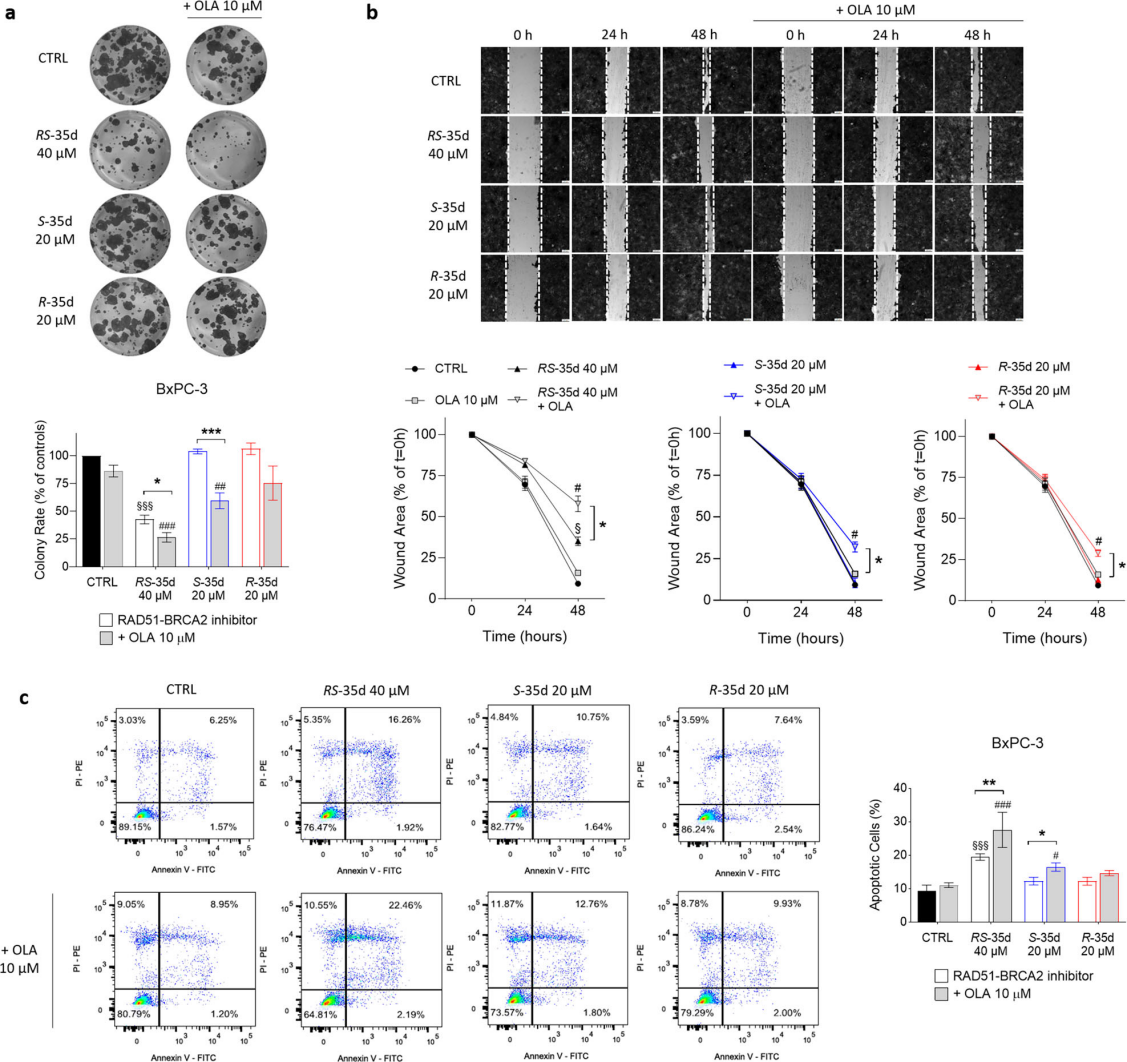
*RS*-35d, *S*-35d and *R*-35d differentially influence cancer hallmarks of tumour aggressiveness. Evaluation of BxPC-3 cell proliferation (**a**), migration (**b**) and apoptosis (**c**) after exposure to 40 µM *RS*-35d, 20 µM *S*-35d or 20 µM *R*-35d alone or in combination with 10 µM OLA. **a** Representative images of BxPC-3 colonies and analysis of the corresponding colony rate (expressed as % of CTRL). **b** Representative images of BxPC-3 wound-healing scratches and analysis of the corresponding migration rate (expressed as % of the wound area at t = 0 h). **c** Representative dot-plots of PI- and FITC-Annexin V-stained BxPC-3 cells analysed through flow cytometry and analysis of the measured apoptotic cells (%). Results are expressed as mean ± SD (n = 3). Statistical analysis was performed with one-way ANOVA followed by Tuckey’s multiple comparison test, ^§^*p* < 0.05 or ^§§§^*p* < 0.001 vs CTRL; ^#^*p* < 0.05, ^##^*p* < 0.01 or ^###^*p* < 0.001 vs 10 µM OLA; *p < 0.05, **p < 0.01 or ***p < 0.001 vs RAD51-BRCA2 inhibitor alone.

Increasing literature data correlate DNA repair pathways deregulation with cancer initiation and progression [30] and DNA repair mechanisms play a pivotal role in modulating cancer risk, progression and therapeutic response [31]. RAD51 has been implicated in PDAC cell proliferation [18], as well as in cancer progression and metastasis in other tumour types [32], indicating a correlation between successful DNA repair and the acquisition of cancer hallmarks of an aggressive tumour phenotype. To investigate whether disrupting RAD51-BRCA2-mediated HR together with PARP inhibition could be a feasible strategy to target cancer aggressiveness, we assessed the effects of *RS*-35d, *S*-35d and *R*-35d on cancer cell proliferation, migration and survival. Colony formation experiments revealed that 40 µM *RS*-35d alone significantly decreased BxPC-3 cell colony rate, which was further impaired upon olaparib coadministration (Fig. 3a). Conversely, *S*-35d and *R*-35d alone did not reduce cancer cell proliferation, but *S*-35d synergised with olaparib to significantly reduce colony rate compared to *S*-35d alone. Wound-healing experiments showed that *RS*-35d/olaparib, *S*-35d/olaparib and *R*-35d/olaparib combinations significantly reduced cell migration compared to the respective single treatment, but only *RS*-35d decreased BxPC-3 migration even when administered alone (Fig. 3b). As for cell survival, flow cytometry-assisted apoptosis analysis (Supplementary Fig. S6) via Annexin V/PI staining revealed a higher percentage of apoptotic cells upon exposure to both *RS*-35d/olaparib and *S*-35d/olaparib combinations compared to *RS*-35d or *S*-35d alone, while *R*-35d/olaparib combination appeared ineffective (Fig. 3c). Moreover, *RS*-35d alone was able to increase apoptotic cell rate in line with previous observations on cell viability/death, proliferation and migration. Overall, these data suggest that while *S*-35d synergistic action with olaparib significantly affects cancer hallmarks of tumour aggressiveness compared to *R*-35d, the treatment with *RS*-35d racemate exerts effects that cannot be entirely recapitulated by the single enantiomers. Hence, we analysed these RAD51-BRCA2 inhibitors within experimental conditions featuring a further level of cellular complexity to better characterise their antineoplastic profile.

### *RS*-35d, but not *S*-35d nor *R*-35d, retains synergism with PARPi in PDAC three-dimensional cultures

**Fig. 4:**
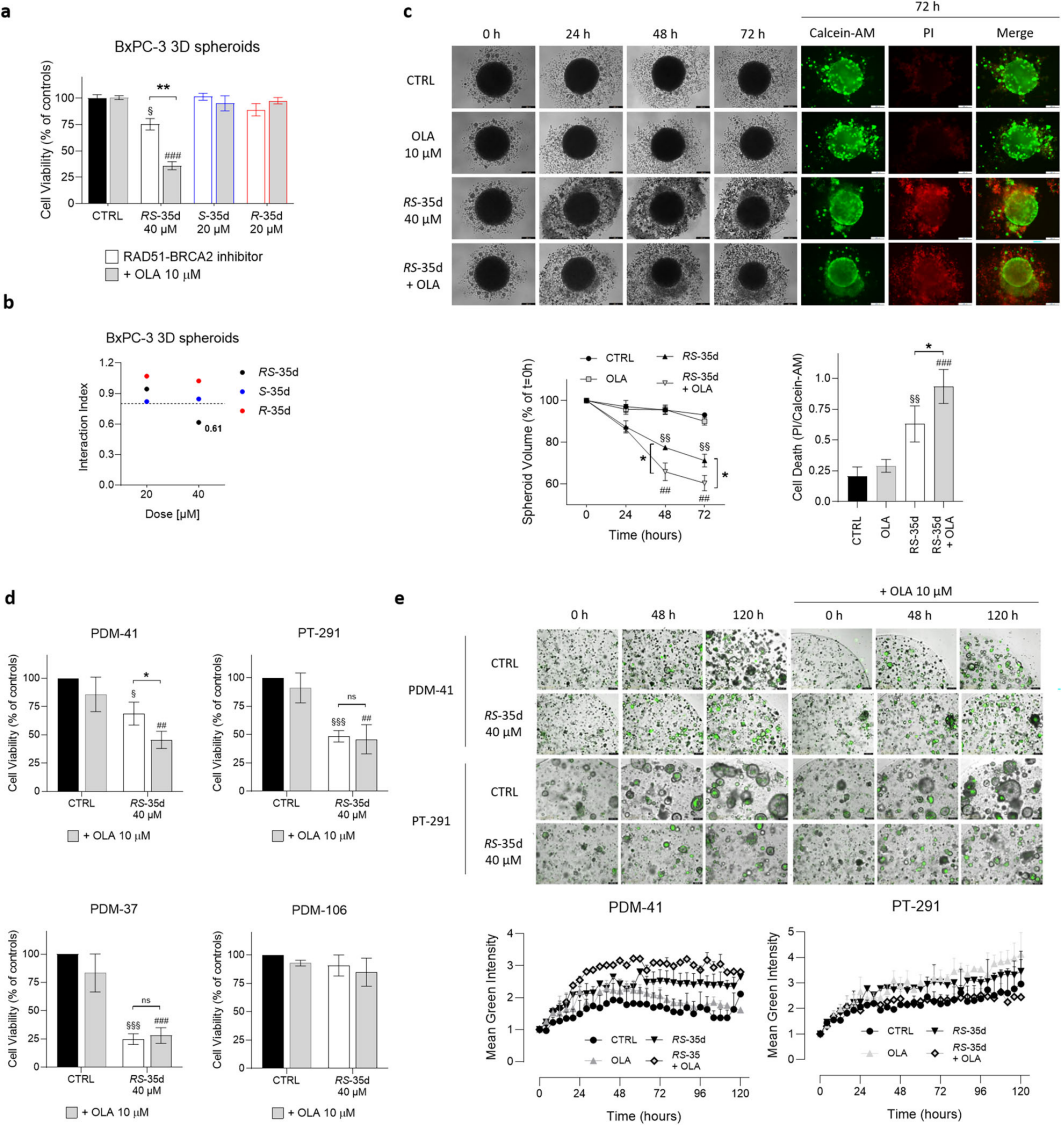
*RS*-35d, *S*-35d and *R*-35d differential effect in PDAC 3D cultures. **a** Cell viability measured after 72 h exposure to 40 µM *RS*-35d, 20 µM *S*-35d or 20 µM *R*-35d alone or in combination with 10 µM olaparib (OLA) in BxPC-3 3D spheroids. **b** I. index of *RS*-35d, *S*-35d or *R*-35d association with olaparib in BxPC-3 3D spheroids. I. index values were made explicit when the drug association showed synergism (i. index < 0.8). **c** Evaluation of the effect of the 72 h treatment with 40 µM *RS*-35d alone or in combination with 10 µM OLA on BxPC-3 3D spheroid volume and cell death; time-course brightfield and fluorescence images of BxPC-3 3D spheroids treated as previously described (scale bar, 200 μm); time-course analysis of the corresponding 3D spheroid volume (expressed as % of the spheroid volume at t = 0 h); analysis of cell death in 3D spheroid at 72 h (expressed as PI/Calcein-AM ratio). Results are expressed as mean ± SD (n = 6). Evaluation of cell viability (**d**) and apoptosis (**e**) upon 120 h treatment with 40 µM *RS*-35d alone or in combination with 10 µM OLA in human PDAC organoids; time-course representative images of *RS*-35d- and *RS*-35d/olaparib-induced apoptosis via caspase 3/7 cleavage (scale bar, 400 μm) and analysis of the corresponding caspase 3/7 cleavage-induced green fluorescence signal. Results are expressed as mean ± SD (n = 3). Statistical analysis was performed with two-way ANOVA (**a**, **c**, **d**, **e**) or one-way ANOVA (**c**) followed by Tuckey’s multiple comparison test, with ^§^*p* < 0.05, ^§§^*p* < 0.01 or ^§§§^*p* < 0.001 vs CTRL; ^##^*p* < 0.01 or ^###^*p* < 0.001 vs 10 µM OLA; *p < 0.05 or **p < 0.01 vs RAD51-BRCA2 inhibitor alone.

Two-dimensional (2D) cultures notoriously have limited predictive value, since they force cells at a solid-liquid planar interface that is very artificial for most cell phenotypes. Further, they facilitate drug diffusion to cells, well beyond what is possible in a solid tissue [33]. Hence, 3D cultures offer a model that more closely reflects in vivo conditions from a cellular (cell-cell and cell-matrix interactions), diffusional and mechanical point of view [34]. Therefore, after the optimisation of BxPC-3 3D spheroid culturing conditions and the confirmation of their tumour-mimicking properties at both mRNA and protein levels (Supplementary Fig. S7a–c) [35], analyses on BxPC-3 3D spheroids showed that, similarly to 2D data, 40 µM *RS*-35d significantly decreased cell viability, which was further reduced upon coadministration with olaparib (Fig. 4a). Surprisingly, and in contrast with 2D results, *S*-35d and its association with olaparib did not significantly affect cell viability (Fig. 4a). Indeed, only *RS*-35d retained its antitumoral activity and synergism with olaparib (i. index 3D = 0.61) (Fig. 4b). These results cannot be ascribed to *RS*-35d’s better penetration: pure enantiomers and racemic mixture have identical diffusion coefficients. However, the generally higher drug resistance and the more difficult accessibility of the 3D cultured cells (in particular deep in the spheroids) may further enhance the previously seen better performance of *RS*-35d compared to its enantiomeric components [35, 36]. Despite showing synergism at both 20 µM (i. index 2D = 0.71) and 40 µM (i. index 2D = 0.70) in 2D cultures (Fig. 2c), *S*-35d/olaparib combination-induced synthetic lethality was less effective in reducing cell viability compared to *RS*-35d/olaparib combination. To further evaluate *RS*-35d antineoplastic effects, we analysed changes in 3D spheroid volume during a time-course treatment with *RS*-35d or *RS*-35d/olaparib. In agreement with cell viability results, 40 µM *RS*-35d significantly decreased spheroid volume, which was further reduced by olaparib association (Fig. 4c). Moreover, cell death evaluation with Calcein-AM and PI vital dyes (Supplementary Fig. S7d) mirrored spheroid volume analysis, indicating a significantly increased spheroid death upon treatment with *RS*-35d, which was amplified by olaparib coadministration (Fig. 4c). Considering that only *RS*-35d retained a synthetic lethality profile in 3D spheroids, we analysed *RS*-35d and olaparib antineoplastic effects also in human PDAC organoids (Supplementary Fig. S7e). PDM-41, PDM-106 and PDM-37 are primary tumour-derived PDAC, liver metastasis PDAC and squamous cell pancreatic carcinoma organoids respectively. PT-127 and PT-291 are invasive moderately differentiated PDAC and invasive PDAC/cholangiocarcinoma organoids originated from patient-derived xenografts [37]. These in vitro models have more complex organisation, structure and function than most 3D spheroids, and therefore better mimic real tumours [37]. In line with data obtained on 2D and 3D cultures, 40 µM *RS*-35d decreased cell viability, to different extents, of PDM-41, PDM-37 and PT-291 organoids. In particular, *RS*-35d anticancer effect was further and significantly amplified by olaparib coadministration only in PDM-41 (i. index = 0.69), while in PT-291 and PDM-37 already *RS*-35d alone strongly reduced cell viability (Fig. 4d). Conversely, *RS*-35d or its combination with olaparib did not alter cell viability of PDM-106 organoids (Fig. 4d). Interestingly, cell viability results were mirrored by an increased apoptotic rate in PDM-41 organoids in line with results obtained in 2D BxPC-3 cultures, but not in PT-291 (Fig. 4e). Altogether, these results indicate that, despite synergising with olaparib in 2D cultures, *S*-35d does not induce synthetic lethality in 3D spheroids. On the other hand, the racemate retains its antineoplastic effect even in environmental conditions that more closely reflect those faced in vivo, although the different effect of *RS*-35d observed on the different PDAC cell lines and organoids employed suggests that mutational status of PDAC driver genes (e.g. *KRAS*, *TP53*, *CDKN2A, SMAD4*) and DDR machinery differential expression [24] may dictate the impact of *RS*-35d treatment and type of cell death mode. Altogether, these data indicate that the two enantiomers may have additive/synergistic effects when combined as a racemate and not visible upon single enantiomer exposure due to the simultaneous inhibition of additional targets.

### *RS*-35d-mediated DDR sensor kinases inhibition and intrinsic synthetic lethality profile

**Fig. 5:**
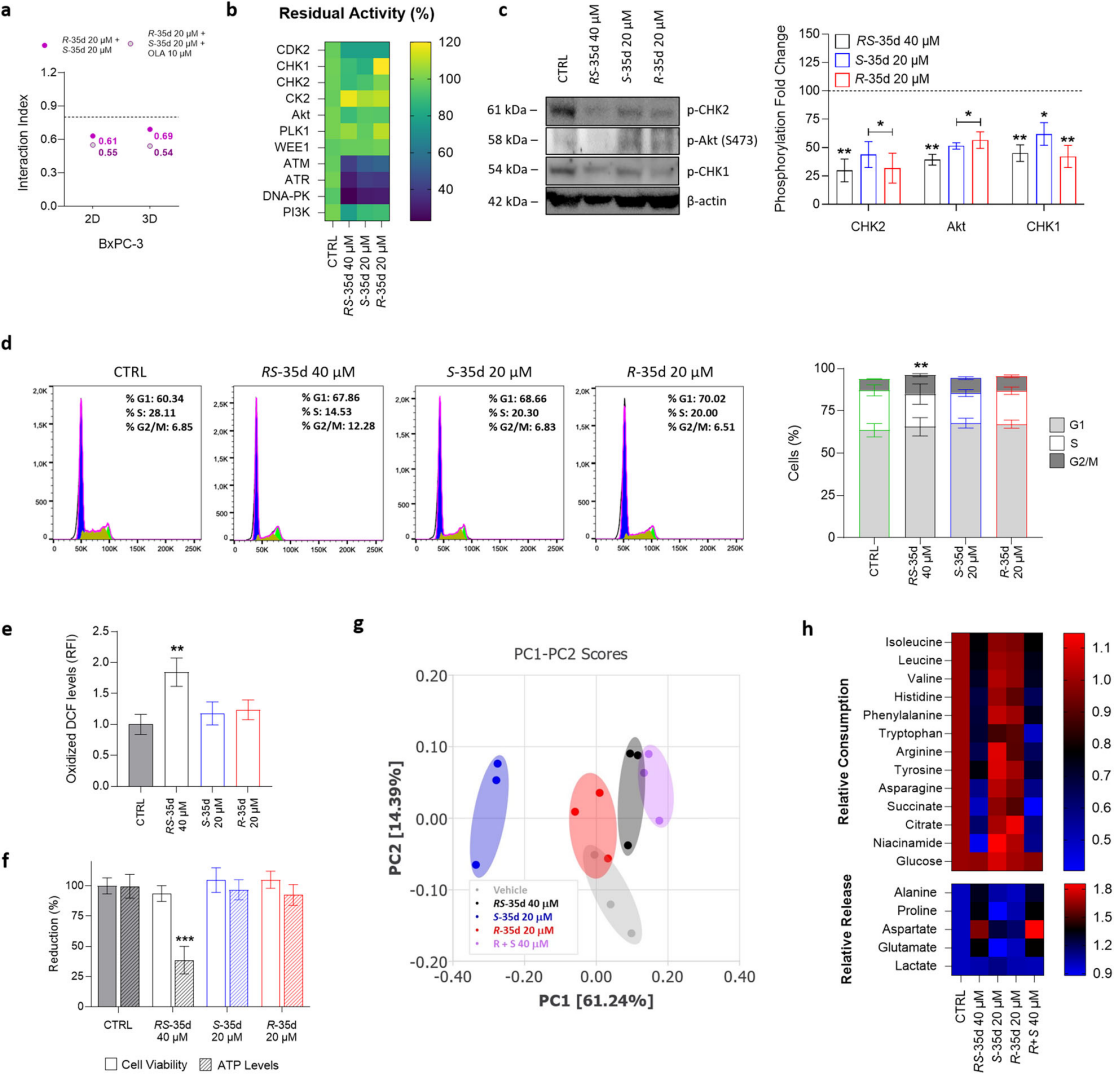
*RS*-35d, *S*-35d and *R*-35d inhibition of DDR kinases and dissection of *RS*-35d mechanism of action. **a** 2D and 3D i. indices of the reconstituted racemic mixture (20 µM *S*-35d + 20 µM *R*-35d) and their association with 10 µM olaparib (OLA). **b** *RS*-35d, *S*-35d and *R*-35d inhibitory effect, expressed as residual activity (% of controls), on a panel of DDR cascade kinases. Results are expressed as mean ± SD (n = 2). **c** Western blot analysis of p-CHK1, p-CHK2, p-Akt (S473) levels in BxPC-3 cells treated with 40 µM *RS*-35d, 20 µM *S*-35d or 20 µM *R*-35d for 24 h (DMSO 0.6% as vehicle control, CTRL). The images are representative Western blots. Effect of 40 µM *RS*-35d, 20 µM *S*-35d or 20 µM *R*-35d treatment for 24 h on cell cycle phases (**d**), ROS production (**e**) and ATP levels (**f**). Unsupervised PCA score plot (**g**) and relative metabolites consumption and release (**h**) of culture media of BxPC-3 cells exposed to 40 µM *RS*-35d, 20 µM *S*-35d or 20 µM *R*-35d for 24 h (DMSO 0.6% as vehicle control, CTRL); **g** 95% confidence ellipses are displayed for each group; **h** heatmap of the significative consumption/release (relative to CTRL values) of metabolites identified from univariate analysis. Results are expressed as mean ± SD (n = 3). Statistical analysis was performed with one-way (**b**, **e**, **h**) or two-way ANOVA (**c**, **d**, **f**) followed by Tuckey’s (**b**–**f**) or Bonferroni’s (**h**) multiple comparison test, with *p < 0.05, **p < 0.01 or ***p < 0.001 vs CTRL.

To investigate if *RS*-35d enantiomers synergistically act when combined in the same proportions of the racemate, we analysed the effect of the reconstituted racemic mixture of *S*-35d and *R*-35d (Supplementary Fig. S8). Besides exerting effects completely superimposable, both in 2D and 3D conditions, with those of the racemate, data obtained with the reconstituted racemic mixture indicate that *S*-35d and *R*-35d display an intrinsic synergism (i. index 2D = 0.61; i. index 3D = 0.69), which was even stronger when combined with olaparib (i. index 2D = 0.55; i. index 3D = 0.54) (Fig. 5a). These results are in line with the reduced cell viability observed upon treatment with *RS*-35d alone in both 2D (Fig. 2a) and 3D (Fig. 4a) and suggest that *S*-35d and *R*-35d, by simultaneously inhibiting putative additional targets together with RAD51, may display a built-in, fully chemical-induced synthetic lethality that could explain *RS*-35d cytotoxic effect. Generally, synthetic lethality arises upon inhibition of parallel, mutually compensatory pathways required for the same or related essential functions [20]. Noteworthy, HR and other DDR pathways were reported to be hotspots for negative genetic interactions exploitable for synthetic lethality [20]. Scientific research particularly focused on DDR sensor kinases ataxia-telangiectasia mutated (ATM), ataxia-telangiectasia and Rad3-related protein (ATR) and DNA-dependent protein kinase (DNA-PK) as pivotal synthetic lethal partners/pair members [38], as also demonstrated by the high number of ATM, ATR and DNA-PK inhibitors currently undergoing clinical trials [2]. Considering that (i) *RS*-35d was designed to inhibit RAD51-BRCA2 interaction [14], (ii) HR and other DDR pathways have been reported as hotspot for negative genetic interaction [20], (iii) DDR kinases are being studied to evaluate their potential as drug target within synthetic lethality strategies and (iv) *RS*-35d enantiomers were found to intrinsically synergise (Fig. 5a, Supplementary Fig. S8), we wanted to investigate if *RS*-35d, *S*-35d and *R*-35d could inhibit DDR kinases to deepen their individual contribution in terms of inhibitory effects on synthetic lethal partners of RAD51. Therefore, we assessed the putative inhibitory effect of the racemate and the two enantiomers on a panel of selected kinases belonging to different DDR pathways that may lead to synthetic lethality upon their concurrent inhibition [2, 39]. Among all tested kinases, only ATM, ATR and DNA-PK showed a statistically significant reduction upon treatment with 40 µM *RS*-35d (*p* < 0.001) (Fig. 5b). Noteworthily, also 20 µM *S*-35d and 20 µM *R*-35d decreased their activity (*p* < 0.001), indicating that their inhibition is mediated by both enantiomers rather than either one of the two (Fig. 5b). Moreover, molecular docking calculations performed to gain structural insight into *S*-35d and *R*-35d interactions with ATM, ATR and DNA-PK further corroborated our biochemical data obtained on the aforementioned kinases (Supplementary Figs. S9–S11). Finally, to provide evidence of ATM, ATR and DNA-PK inhibition mediated by *RS*-35d, *S*-35d and *R*-35d in PDAC cells, we evaluated the phosphorylation levels of ATR, ATM and DNA-PK direct downstream targets CHK1, CHK2 and Akt respectively [39, 40] after a 24 h treatment (a timing without visible cytotoxic effects based on preliminary screenings) with RAD51-BRCA2 inhibitors in BxPC-3 cells. In line with previous results, the early exposure to *RS*-35d, *S*-35d and *R*-35d reduced the phosphorylation of CHK1, CHK2 and Akt, further strengthening computational and biochemical data on DDR sensor kinases inhibition (Fig. 5c). Literature data indicate that HR impairments due to RAD51 depletion or blockade result in cell cycle arrest at G2/M phase. This ultimately increases reactive oxygen species (ROS) accumulation, inducing nuclear DNA damage and impairing mitochondrial functions, which in turn further exacerbates nuclear DNA damage [18, 41–43]. However, also DDR kinases ATM, ATR and DNA-PK have been described to play a pivotal role in cell cycle progression, ROS regulation and mitochondrial activity [39, 41, 44, 45]. Considering its ability to inhibit RAD51-BRCA2 interaction as well as DDR sensor kinases, to completely elucidate *RS*-35d mechanism of action, we analysed cell cycle state, ROS levels and mitochondrial activity upon an early exposure (i.e. 24 h) to the racemate and the two enantiomers. Despite single enantiomer treatments at 20 µM did not significantly impact any of the analysed cell features, 40 µM *RS*-35d induced a cell cycle arrest in the G2/M phase (Fig. 5d), an increase in ROS production (Fig. 5e) and a reduction in ATP levels (Fig. 5f) (see also Supplementary Fig. S12). Data obtained with lower (20 µM) *RS*-35d and higher (40 µM) *S*-35d and *R*-35d concentration (as well as those obtained with the reconstituted racemic mixture) at the same timings confirm that the observed effects on cell cycle, ROS levels and mitochondrial efficiency are a consequence of the exerted mechanism of action (Supplementary Fig. S12). Exometabolomic analyses on growth media were then performed to further substantiate data obtained on mitochondrial function and redox state. Culture media of cells exposed for 24 h to the different compounds were analysed through ^1^H nuclear magnetic resonance (NMR) spectroscopy. Besides indicating a partial (only for *R*-35d) or complete separation of the treated groups compared to the controls, the unsupervised Principal Component Analysis (PCA) of the growth media NMR spectra revealed a clear separation of 20 µM *S*-35d and 20 µM *R*-35d groups from the 40 µM *RS*-35d group, which is in turn overlapping with the 40 µM *R* + *S* group (Fig. 5g). This further corroborates the hypothesis that the racemate (and reconstituted racemate) induces cellular effects not observed upon single enantiomer exposure. To gain further insight into energetic/oxidative alterations resulting from *RS*-35d mechanism of action, we analysed the consumption and the production/release of metabolites in culture media, which has been proven as a reliable approach to indirectly investigate the metabolic, energetic and redox status of the cell [46, 47]. In this regard, statistically significant (p < 0.05 as reference value, one-way ANOVA followed by Bonferroni’s post hoc test) metabolic modulations were found only for 40 µM *RS*-35d and 40 µM *R* + *S*, while 20 µM *S*-35d and 20 µM *R*-35d NMR signals were comparable to the vehicle control. In particular, the reduced consumption of tricarboxylic acid (TCA) cycle intermediates (i.e. citrate and succinate) [48], branched-chain amino acids (BCAA; i.e. isoleucine, leucine, valine) [49, 50] and other amino acids (i.e. histidine, phenylalanine, tryptophan, tyrosine, arginine, asparagine) [46, 51, 52] (Fig. 5h)—together with unchanged glycolytic activity due to unaltered glucose consumption and lactate production for the different treatments—suggests a dysfunctional mitochondrial activity and strengthen previous results obtained on ATP levels. In parallel, a reduction in niacinamide consumption [46] and an increased release of alanine [53], proline, glutamate and aspartate [46, 51, 52] (Fig. 5h) may be indicative of an increased/altered oxidative stress, further supporting oxidised DCF results. Altogether, these findings are in line with literature data on RAD51 and DDR kinases inhibition and reveal *RS*-35d complex mechanism of action of intrinsic cytotoxic effect as a resultant of *S*-35d and *R*-35d synergistic action.

### *RS*-35d proteomic profile in *BRCA2*-proficient PDAC cells

**Fig. 6:**
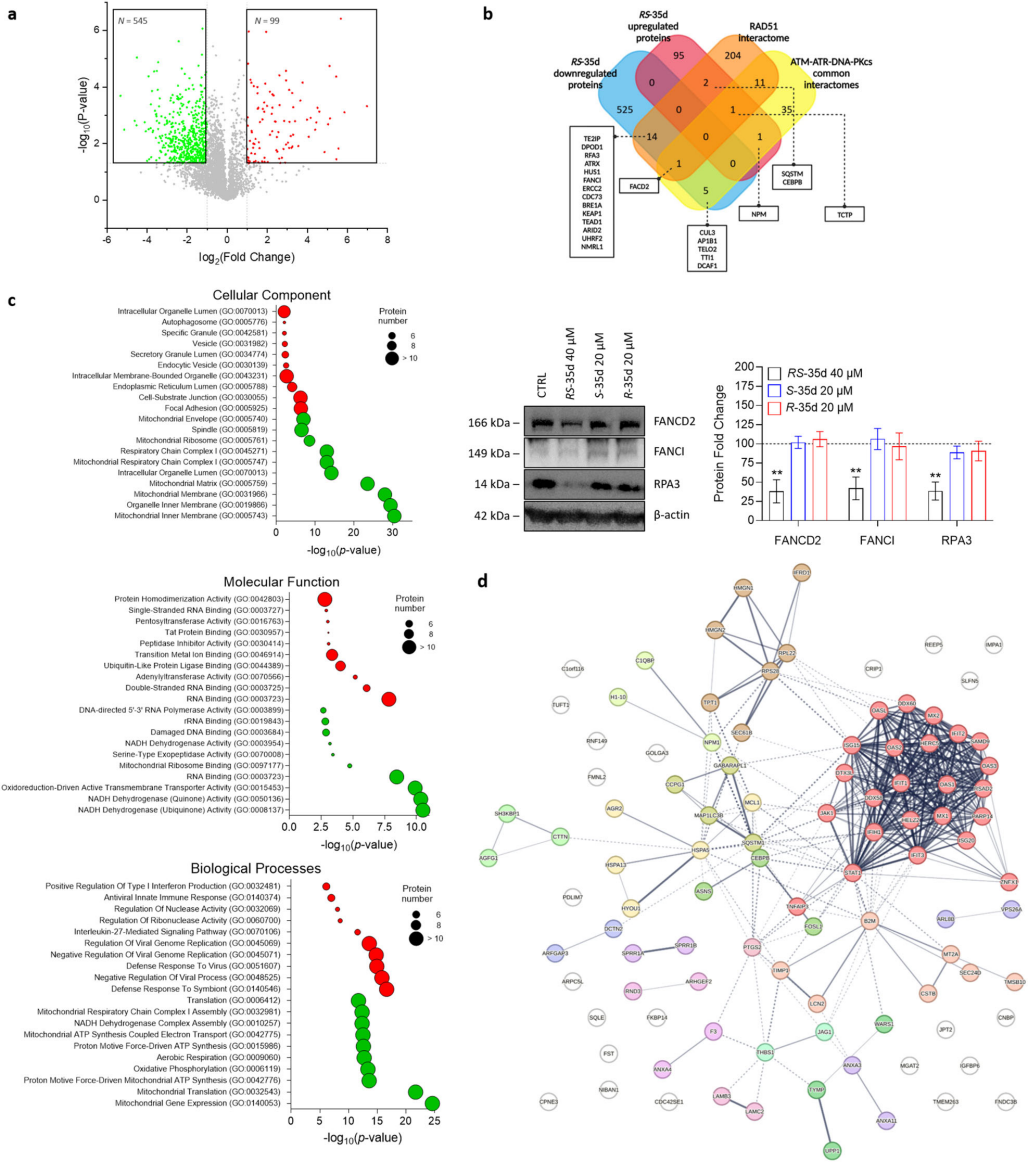
*RS*-35d proteomic profile in BxPC-3 cells. **a** Volcano plot of control vs *RS*-35d up- (red) and downregulated (green) proteins in BxPC-3 cells, *p*-value < 0.05 and log_2_(fold change) > 1 or < −1 were used as significance cut-off. **b** Venn diagram of *RS*-35d up- and downregulated common proteins with RAD51 interactome and ATM/ATR/DNA-PK common interactomes; Western blot analysis of FANCD2, FANCI and RPA3 expression in BxPC-3 cells treated with 40 µM *RS*-35d, 20 µM *S*-35d or 20 µM *R*-35d for 24 h. The images are representative Western blots. Results are normalised over β-actin expression and expressed as mean ± SD (n = 3). Statistical analysis was performed with two-way ANOVA followed by Dunnett’s multiple comparison test, with **p < 0.01 vs CTRL. **c** Gene Ontology (GO) cellular component, molecular functions and biological processes enriched from the analyses of the up- (red) and downregulated (green) proteins by *RS*-35d. **d** STRING functional analysis of up-regulated proteins by *RS*-35d. Proteins were clustered using Markov clustering (MCL), with default inflation parameter = 3; the thickness of lines representing protein-protein interactions indicates the strength of data support.

We previously demonstrated that the BRC4 peptide-induced inhibition of RAD51-BRCA2 interaction caused specific proteomic alterations, particularly within DDR pathways [13]. Considering the racemate ability to inhibit not only RAD51-BRCA2 interaction but also multiple regulators of DDR mechanisms (i.e. ATM, ATR and DNA-PK), a mass spectroscopy (MS) campaign was performed to investigate the protein expression changes induced by *RS*-35d in BxPC-3 cells. *RS*-35d proteomic profiling allowed the identification of a molecular fingerprint of 3603 proteins, which were screened for proteomic hits (Fig. 6a). A total of 644 proteins were differentially regulated by *RS*-35d treatment, of which 99 were upregulated and 545 downregulated (the full list of proteins is provided in the Supplementary Information). When comparing the up- and downregulated proteins of BRC4 to the proteomic alterations caused by *RS*-35d, only a partial overlap was evident (Supplementary Fig. S13a). This is consistent with the distinct mechanisms of action for BRC4 and *RS*-35d and prompted further investigations into the proteomic fingerprint of the small molecule. Hence, *RS*-35d proteomic hits were compared to RAD51, ATM, ATR, and DNA-PK interactomes (the full list of proteins is provided in the Supplementary Information) to identify *RS*-35d-linked proteomic signatures relevant to the disruption of such targets and to further shed light on its mechanism of action (Fig. 6b) (detailed description of depicted proteins is reported in Table 1). Common downregulated proteins within RAD51 interactome well recapitulated our previous findings, where we highlighted that alterations of FANCD2, FANCI and RPA3 levels could serve as potential indicators of DDR impairment (Supplementary Fig. S13b) [13]. Based on these results that strongly correlate *RS*-35d treatment and DNA damage (see Table 1), we evaluated if *RS*-35d, *S*-35d or *R*-35d could alter FANCD2, FANCI and RPA3 protein levels as a consequence of DDR impairment. Similarly to BRC4 treatment [13], *RS*-35d induced their downregulation, while exposure to either *S*-35d or *R*-35d separately did not alter their protein levels (Fig. 6b). These data strengthen the hypothesis that the simultaneous action of both enantiomers is pivotal for *RS*-35d to significantly impact on DDR mechanisms and exert its antineoplastic effects as a resultant of *S*-35d and *R*-35d synergistic action, conferring a serendipitous, intrinsic synthetic lethal profile to the racemate. To gain further insight into *RS*-35d protein fingerprint, we performed a functional enrichment analysis on the differentially expressed proteins (DEPs) to identify their cellular localisation, biological processes, and molecular function (Fig. 6c) (detailed description of enriched pathways is reported in the Supplementary Information). Downregulated proteins were found to be mainly involved in DNA damage and mitochondrial activity (Supplementary Fig. S13c), consistently with previous observations of *RS*-35d mechanism of action (Fig. 5) and *RS*-35d-mediated DDR impairment (Fig. 6b). Interestingly, upregulated proteins were found to be particularly enriched in pathways of nucleic acid binding, type I interferon (IFN) response and defence response to virus (Fig. 6c), suggesting that *RS*-35d may up-regulate pathways involved in response to cytoplasmic nucleic acids. In this regard, our STRING analysis revealed that upregulated proteins that are most and significantly correlated belong to the Retinoic acid-inducible gene I (RIG-I)/melanoma differentiation-associated protein 5 (MDA5) pathway (red cluster) (Fig. 6d) [54]. This is in line with *RS*-35d ability to induce micronuclei formation (Fig. 1g) and to down-regulate AP1B1 (recently reported to control termination of cyclic GMP-AMP synthase (cGAS)/stimulator of interferon genes (STING) signalling, a parallel cytosolic DNA-triggered cascade in crosstalk with RIG-I/MDA5 pathway [55]) (Fig. 6b; Table 1) and is consistent with literature data of RIG-I/MDA5 pathway activation in response to increased micronuclei formation upon DDR blockade [56]. Building upon the chemically-induced synthetic lethality paradigm, our data indicate that *RS*-35d not only can broadly affect DDR mechanisms and genomic stability beyond RAD51-BRCA2 interaction, but also can potentially elicit additional cellular responses of oncological interest and immunotherapy-correlated pharmacological strategies [56] as a consequence of the exerted cell death mechanism.

**Table 1:** *RS*-35d DEPs common to RAD51, ATM, ATR and DNA-PK interactomes.

Protein	Gene	log_2_ (FC)	−log (*p*-value)	Pathway/role in PDAC		Ref.
CEBPB	*CEBPB*	1.29	1.85	Transcription factor involved in inflammatory responses; Tumour suppressor in PDAC		[93, 94]
NPM	*NPM1*	1.07	5.96	Involved in genomic stability; Promoter of aerobic glycolysis and cell cycle regulator in PDAC		[95–97]
SQSTM	*SQSTM1*	1.20	4.15	Autophagy and oxidative stress regulator also in PDAC		[98]
TCTP	*TPT1*	1.56	1.34	DNA damage-responsive and involved in PDAC cell growth and progression		[99, 100]
AP1B1	*AP1B1*	−1.71	3.00	Controls cGAS/STING pathway termination		[101]
ARID2	*ARID2*	−1.91	1.64	Major component of the SWI/SNF chromatin-remodelling complex		[102]
ATRX	*ATRX*	−1.88	1.49	Chromatin-remodelling factor linked to KRAS-mediated PDAC promotion		[103]
BRE1A	*RNF20*	−2.10	1.91	Promotes chromosome segregation and HR as part of RPA/RNF20/SNF2H cascade		[104]
CDC73	*CDC73*	−1.03	1.42	Associated with PDAC patients with the germline pathogenic variant *CDKN2A*+		[105]
CUL3	*CUL3*	−1.44	1.57	Promotes tumour growth as part of the KEAP1/Nrf2/CUL3 axis		[106]
DCAF1	*DCAF1*	−1.83	1.34	Linked with cell cycle, cell growth, cell division, cell survival and tumorigenesis in PDAC		[107]
DPOD1	*POLD1*	−2.90	1.34	3′-exonuclease domain-containing DNA polymerase linked with genome stability		[108]
ERCC2	*ERCC2*	−2.26	2.10	NER mediator required for DNA unwinding; linked to gemcitabine/CDDP polychemotherapy resistance		[109]
FACD2	*FANCD2*	−3.45	2.51	HR efficiency-related; Pro-tumorigenic role; linked to platinum sensitivity/resistance also in PDAC		[13, 110, 111]
FANCI	*FANCI*	−1.76	2.07	HR efficiency-related; linked to platinum sensitivity/resistance also in PDAC		[13, 110]
HUS1	*HUS1*	−1.77	1.53	Part of the 9-1-1 DNA checkpoint clamp involved in DNA DSBs repair; proposed synergism with PARGi in PDAC		[112, 113]
KEAP1	*KEAP1*	−1.20	2.53	Promotes tumour growth as part of the KEAP1/Nrf2/CUL3 axis		[106, 114]
NMRL1	*NMRAL1*	−2.47	1.85	Prevents apoptosis in PDAC		[115]
RFA3	*RPA3*	−3.53	1.41	HR efficiency-related; necessary for replication in various cell types		[13, 116]
TE2IP	*TERF2IP*	−3.34	1.74	Part of a prognostic signature of 15 inflammation-related gene markers in PDAC		[117]
TEAD1	*TEAD1*	−3.79	1.52	Associated with PDAC progression		[118, 119]
TELO2	*TELO2*	−1.23	2.10	Regulator of mTOR activity		[120]
TTI1	*TTI1*	−2.30	1.41	TELO2 regulator; takes part in mTOR regulation		[121]
UHRF2	*UHRF2*	−2.15	2.02	Pro-growth effect; overexpression linked with lower DFS and OS in CRC patients		[122]

*AP1B1* adaptor related protein complex 1 subunit beta 1, *ARID2* AT-rich interactive domain-containing protein 2, *ATRX* ATRX chromatin remodeler, *BRE1A* RING-type E3 ubiquitin transferase BRE1A, *CDC73* cell division cycle 73, *CEBPB* CCAAT/enhancer-binding protein beta, *CRC* colorectal cancer, *CUL3* cullin 3, *CDKN2A* cyclin-dependent kinase inhibitor 2A, *DCAF1* DDB1 and CUL4 associated factor 1, *DFS* disease-free survival, *DPOD1* DNA polymerase delta catalytic subunit, *ERCC2* ERCC excision repair 2, TFIIH core complex helicase subunit, *FACD2* Fanconi anaemia complementation group D2, *FANCI* Fanconi anaemia complementation group I, *FC* fold change, *HUS1* HUS1 checkpoint clamp component, *KEAP1* Kelch-like ECH-associated protein 1, *KRAS* Kirsten rat sarcoma virus, *mTOR* mammalian target of rapamycin, *NER* nucleotide excision repair, *NMRL1* NmrA like redox sensor 1, *NPM* nucleophosmin, *Nrf2* nuclear factor erythroid 2-related factor 2, *OS* overall survival, *PARGi* poly (ADP-ribose) glycohydrolase (PARG) inhibitor, *RFA3* replication factor A protein 3, *SQSTM* sequestosome 1, *SWI/SNF* switch/sucrose non-fermentable, *TCTP* translationally controlled tumour protein, *TE2IP* telomeric repeat-binding factor 2-interacting protein 1, *TEAD1* TEA domain transcription factor 1, *TELO2* telomere length regulation protein TEL2 homologue, *TTI1* TELO2 interacting protein 1, *UHRF2* ubiquitin like with PHD and ring finger domains 2.

### *RS*-35d built-in synthetic lethality overcomes PARPi resistance in *BRCA2*-mutated PDAC cells

**Fig. 7:**
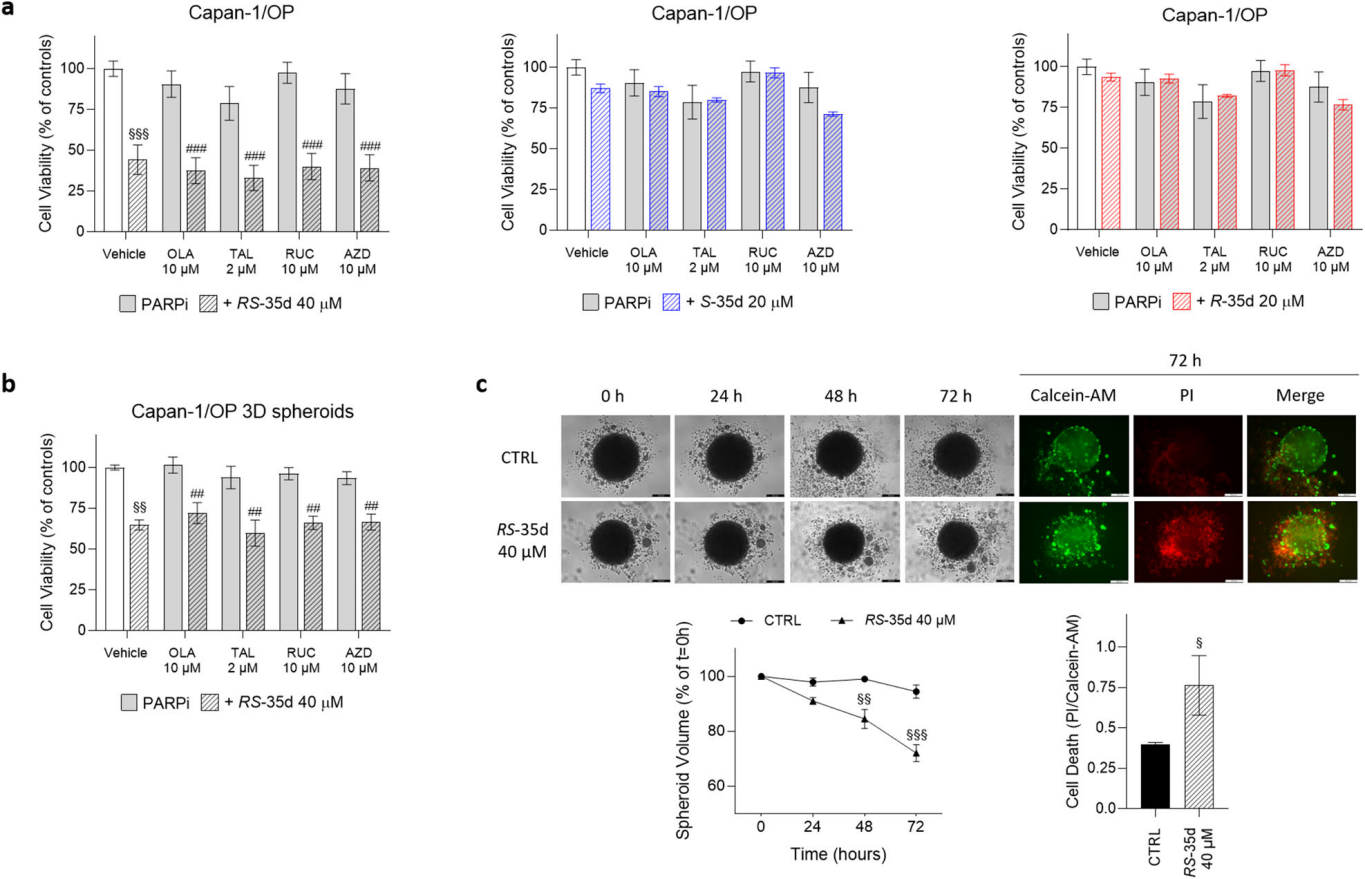
*RS*-35d exerts intrinsic synthetic lethality in PARPi-resistant, *BRCA2*-mutated Capan-1/OP 2D and 3D cultures. Cell viability after 72 h exposure to 10 µM olaparib (OLA), 2 µM talazoparib (TAL), 10 µM rucaparib (RUC) or 10 µM AZD2461 (AZD), alone or in combination with 40 µM *RS*-35d, 20 µM *S*-35d or 20 µM *R*-35d in Capan-1/OP 2D (**a**) and 3D (**b**) cultures. **c** Evaluation of the effect of the 72 h treatment with 40 µM *RS*-35d on Capan-1/OP 3D spheroid volume and cell death. Time-course representative brightfield and fluorescence images of Capan-1/OP 3D spheroids treated as previously described (scale bar, 200 μm), analysis of the corresponding 3D spheroid volume (expressed as % of the spheroid volume at t = 0 h) and cell death in 3D spheroid at 72 h (expressed as PI/Calcein-AM ratio). Results are expressed as mean ± SD (n = 6). Statistical analysis was performed with Student’s t-test (**c** *right*) or two-way ANOVA (**a**, **b**, **c** *middle*), followed by Tuckey’s multiple comparison test, with ^§^*p* < 0.05, ^§§^*p* < 0.01 or ^§§§^*p* < 0.001 vs CTRL; ^##^*p* < 0.01 or ^###^*p* < 0.001 vs PARPi alone.

As reported in the Introduction section, different PARPi were developed to induce synthetic lethality in cancer patients harbouring *BRCA1/2* mutations and HR deficiencies, leading to selective cancer cell death. To date, several PARP-targeting inhibitors have been developed and approved for multiple clinical indications, while more recent or ongoing clinical trials aim to further expand their use [2]. However, as also experienced with several other anticancer agents, the emergence of resistances in patients receiving PARPi treatments is often observed. There are multiple mechanisms at the basis of PARPi resistance, including PARP-related effects, restoration of HR function, replication fork stability restoration and replication gaps repair (reviewed in [57]). Different pharmacological approaches designed to enhance PARPi treatment effects or replace PARPi use are currently under clinical investigation. These association treatment strategies combine a PARPi with various anticancer agents, including inhibitors of multiple DDR players (e.g. WEE1, CHK1/2, ATR, ATM and DNA-PK), to prevent or overcome PARPi resistance [58, 59]. Hence, considering its ability to concurrently inhibit RAD51-BRCA2 interaction as well as ATM, ATR and DNA-PK, we investigated if exposure to 40 µM *RS*-35d, 20 µM *S*-35d or 20 µM *R*-35d, alone or in combination with multiple PARPi (olaparib, talazoparib, rucaparib and AZD2461), could result in intrinsic/more robust synthetic lethality also in olaparib-resistant, *BRCA2*-mutated Capan-1 (Capan-1/OP) cells (Supplementary Fig. S14). While PARPi single treatment did not exert any cytotoxic effect after 72 h exposure, 40 µM *RS*-35d alone significantly affected cell viability in both 2D (Fig. 7a) and 3D cultures (Fig. 7b). In addition, both 20 µM *S*-35d and 20 µM *R*-35d did not exert any anticancer effect either alone or in combination with the different PARPi in 2D cultures (Fig. 7a). The lower *RS*-35d impact on Capan-1/OP spheroids compared to 2D cultures can be attributed to 3D models’ higher drug resistance [35, 36], consistent with data obtained on BxPC-3 3D spheroids. To note, *RS*-35d did not synergise with any of the tested PARPi, indicating that the observed cytotoxic effects on Capan-1/OP cultures were mediated solely by *RS*-35d. In this regard, 40 µM *RS*-35d 72 h exposure significantly decreased spheroid volume and increased cell death (Fig. 7c), in line with data obtained on BxPC-3 3D cultures. Altogether, these data indicate that the multiple target inhibition exerted by *RS*-35d can elicit intrinsic synthetic lethality not only in *BRCA2*-proficient cells, but also in *BRCA2*-mutated, olaparib-resistant PDAC cells, suggesting *RS*-35d as a potential anticancer agent to possibly overcome PARPi resistance.

### *RS*-35d nanoparticles suppress *BRCA2*-proficient and *BRCA2*-mutated/PARPi-resistant cells growth

Despite its interesting pharmacologic profile due to the intrinsic synergism exerted by its two enantiomers, *RS*-35d possesses physical and chemical properties that poorly support its development as a therapeutic agent, including low aqueous solubility (<1 µM) [14]. Since our previous medicinal chemistry campaign found limitations in improving *RS*-35d solubility and target potency (patent: WO2021116999A1), we developed a nanoparticle formulation to improve *RS*-35d solubility and drug delivery (Supplementary Fig. S15a). These particles (hereafter termed *RS*-35d NPs) were produced by (nano)precipitation of *RS*-35d in water, using vitamin E poly(ethylene glycol) succinate (TPGS) as a surfactant, and had a 2 mM *RS*-35d concentration, a Z-average particle size of ~250 nm, a polydispersity index (PDI) of ~0.24 and a ζ-potential of -6 mV (Supplementary Fig. S15b–d). TPGS is a generally regarded as safe (GRAS) component of nanoformulations [60] and specifically of drug nanocrystals [61]. We studied *RS*-35d NPs anticancer activity in both *BRCA2*-proficient (BxPC-3) and *BRCA2*-mutated PARPi-resistant (Capan-1/OP) PDAC cells. We observed that *RS*-35d NPs effectively suppressed cell viability in both PDAC cell lines in a dose-dependent manner (Supplementary Fig. S15e). This suggests that the nanoparticle approach not only can improve *RS*-35d dispersability, but may even potentiate its pharmacological effects, which may depend on its entrance in cells via endocytosis, where ‘large parcels’ may correspond to transiently very high intracellular concentrations.

## Discussion

Accumulating evidence correlates elevated RAD51 expression and HR-related function with poorer prognosis, metastasis and resistance to chemotherapy and radiotherapy in various tumour types, including PDAC, which remains a significant unmet medical need [17–19]. In this context, RAD51 holds great potential as drug target. Combining a small molecule RAD51-BRCA2 inhibitor with PARPi could effectively target cancer cells, even when *BRCA1/2* genes and HR are fully functional [14–16]. The chemical effort aimed at improving the solubility and potency of the best-in-class compound *RS*-35d capable of inhibiting RAD51-BRCA2 interaction yielded no better-performing molecule in terms of HR inhibition and impact on cell viability (patent: WO2021116999A1). *RS*-35d’s intrinsic ability to affect cell viability even without olaparib coadministration [14] suggested a differential activity of its two enantiomers and prompted an in-depth dissection of their properties. Computational and biophysical analyses indicated clear differential RAD51 binding trends for the two enantiomers, pinpointing *S*-35d as a better RAD51-BRCA2 inhibitor than *R*-35d. Consistently, *S*-35d significantly impaired HR and RAD51 nuclear function compared to *R*-35d, synergising with olaparib to induce marked DNA damage, reduced cell viability/increased cell death, decreased proliferation and migration and increased apoptotic rate. To note, only *RS*-35d racemate showed antineoplastic activity without olaparib co-exposure even in 3D cultures that more closely resemble cancer complexity. The retained antineoplastic effect of the racemate was then linked to *S*-35d and *R*-35d ability to intrinsically synergise by simultaneously inhibiting DDR sensor kinases ATM, ATR and DNA-PK, both in vitro and in cells, in addition to RAD51. By affecting cell cycle regulation, redox state and mitochondrial efficiency as part of its complex mechanism of action, *RS*-35d ultimately led to cancer cell death and possibly elicited cell response pathways of immunotherapy-related oncological interest. Finally, the broad DDR impairment—as evidenced by the downregulation of HR impairment indicators FANCD2, FANCI and RPA3 as well as downregulated proteins in Table 1—exerted by *RS*-35d and its complex mechanism of action were effective in tackling also *BRCA2*-mutated, olaparib-resistant PDAC cells, suggesting *RS*-35d possible contribution to overcome PARPi resistances. Moreover, our proposed *RS*-35d nanoparticle formulation establishes a foundation for enhancing the compound’s solubility and delivery, and lays the groundwork for a more advanced formulation, potentially enriched with tumour-targeting moieties for targeted delivery to pancreatic tumours.

The detailed characterisation of *RS*-35d enantiomers clearly suggests that *S*-35d is the major contributor to the inhibition of HR-related RAD51 activity. This is supported not only by our biochemical, biophysical and computational data, but also by the biological evidence demonstrating the inhibition of RAD51 foci formation/HR activity and synergism with olaparib. Conversely, *R*-35d does not significantly impact these functions, suggesting that, when combined together as a racemate, *S*-35d mostly mediates RAD51 inhibition, while *R*-35d contribution to this aspect is limited. Regarding DDR kinases activity, provided evidence shows that both enantiomers can inhibit ATM, ATR and DNA-PK activity at similar rate. Mechanistically, it is possible to hypothesise that, when administered as a racemate at the effective concentration (i.e. 40 µM), *S*-35d (i.e. 20 µM) inhibits RAD51-mediated HR, while *R*-35d (i.e. 20 µM) can affect ATR, ATM and DNA-PK activity. This is consistent with the limited effect of *R*-35d (alone or combined with olaparib) and with *S*-35d exhibiting stronger RAD51 binding but lesser impact on cell viability compared to *RS*-35d when co-administered with olaparib. Therefore, the stronger effects of *RS*-35d that could not be recapitulated by single enantiomer treatment—as evidenced by data on cell cycle, ROS production and mitochondrial activity as well as FANCD2, FANCI and RPA3 expression—results from the individual effects of *S*-35d and *R*-35d, which synergise and become more effective when combined together as a racemate.

The innovative framework of synthetic lethality has made great strides in discovering of novel synthetic lethal gene pairs and anticancer drug candidates. In this context, a prominent role is held by members of DDR pathways, whose reliance and overexpression in cancer cells prompted their analysis within the synthetic lethality rationale. Besides PARP-BRCA1/2 pairs, DDR sensor kinases ATM, ATR and DNA-PK recently gained attention as synthetic lethal partners [38] in different tumour types, as demonstrated by the increasing number of their inhibitors in clinical trials [2]. In the traditional mechanistic explanation of synthetic lethality, gene pairs function in parallel, mutually compensatory pathways, defined ‘between-pathway synthetic lethality’ (bpSL) [20]. However, *S*-35d and *R*-35d have the ability to inhibit functional entities belonging to the same pathway by simultaneously targeting both upstream (ATM, ATR, DNA-PK) and downstream (RAD51-BRCA2) DDR effectors. This gives rise to a serendipitous, intrinsic synergism that falls within the yet-poorly-investigated paradigm of ‘within-pathway synthetic lethality’ (wpSL), where impairments affecting the same molecular pathway or complex result in multiple negative interactions [20]. This enantiomer built-in synergism pinpoints *RS*-35d as a proof-of-concept small molecule for the wpSL paradigm, recalling a concept firstly expressed a decade ago [20] and here proposed for the first time within the fully small molecule-induced synthetic lethality framework. Building upon the putative off-target effects of *RS*-35d and in the context of wpSL, here we also demonstrate that, with a single molecule, it is possible to concurrently induce BRCAness and to inhibit synthetic lethal partners (i.e. ATM, ATR, DNA-PK). Despite requiring further optimisation, this suggests not only that *RS*-35d/PARPi coadministration results in a more robust synthetic lethality, but also a potential alternative to PARPis able to intrinsically give rise to synthetic lethality alone, thus possibly overcoming PARPi resistance. In this regard, despite not synergising with different PARPi developed to be poor substrates for drug transporters or to target multiple PARP isoforms (e.g. talazoparib and AZD2461), *RS*-35d ability to be effective in *BRCA2*-mutated cancer cells after the emergence of PARPi resistances further suggests the wpSL paradigm as an interesting pharmacological strategy of clinical interest. However, the translation of this strategy into clinic should be considered when the safety profile of the drug is acceptable, bearing in mind that such mechanism of action could rise unwanted side effects that should be carefully balanced by its potency and efficacy. Despite being serendipity-driven and limited to DDR pathways, this study is only the tip of an iceberg that highlights the possibility of exploiting the alleged off-target effects of a new compound to avoid or reduce unwanted side effects and shows the huge room for improvement in the search for targetable synthetic lethal pairs. Nonetheless, this work paves the way for future studies aiming to unveil the hidden world of synthetic lethality pairs network, moving away from the classic PARP-BRCA1/2 pair and to fully exploit the potentials of multitarget small molecule approaches to boost the development of personalised medicine strategies.

## Materials and methods

### Chemicals, reagents and antibodies

Olaparib (PubChem CID: 23725625, #S1060), talazoparib (PubChem CID: 135565082, #S7048), rucaparib (PubChem CID: 9931954, #S4948), and *cis*-diamminedichloroplatinum(II) (CDDP) (PubChem CID: 2767, #S1166) were purchased from Selleck Chemicals (Houston, TX, USA). The BRC4-biotinylated peptide (N-term biotin-KEPTLLGFHTASGKKVKIAKESLDKVKNLFDEKEQ) was purchased from Life Technologies (Carlsbad, CA, USA). *RS*-35d was synthesised as previously detailed [14]. AZD2461 (PubChem CID: 44199317, #SML1858), 2′,7′-dichlorofluorescein diacetate (DCFH-DA) (PubChem CID: 104913, #35845), 4′,6-diamidino-2-phenylindole (DAPI) (PubChem CID: 2954, #D9542), Propidium Iodide (PI) (PubChem CID: 104981, #P4864), 2-(3,5-diphenyltetrazol-2-ium-2-yl)-4,5-dimethyl-1,3-thiazole;bromide (MTT) (PubChem CID: 64965, #M2128) and Crystal Violet (PubChem CID: 11057, #C0775) were obtained from Sigma-Aldrich (St. Louis, MO, USA). Calcein-AM (PubChem CID: 390986, #C3099) and Lipofectamine^TM^ 2000 (#11668019) were purchased from Thermo Fisher Scientific Inc. (Waltham, MA, USA). Primary antibodies against RPA3 (rabbit, #ab97436), FANCI (rabbit, #ab245219), FANCD2 (rabbit, #ab178705), γH2AX (pS139) (rabbit, #ab11174) and GLUT1 (rabbit, #ab115730) were purchased from Abcam (Cambridge, UK). Primary antibodies against β-actin (rabbit, #A2066) and β-tubulin (mouse, #T0198) were obtained from Sigma-Aldrich. Primary antibodies against phospho-CHK1 (Ser345) (rabbit, #2348), phospho-CHK2 (Thr68) (rabbit, #2197) and phospho-Akt (Ser473) (rabbit, #4060S) were obtained from Cell Signaling Technology, Inc. (Danvers, MA, USA). Primary antibodies against RAD51 (rabbit, #70-001), HIF-1α (rabbit, #NB100-449) and CDH1 (mouse, #610182) were from BioAcademia (Osaka, Japan), NOVUS Biologicals (Centennial, CO, USA) and BD Biosciences (Franklin Lakes, NJ, USA) respectively. Host-specific peroxidase-conjugated (HRP) IgG secondary antibodies (anti-rabbit #ab6721, anti-mouse #ab6728) were purchased from Abcam.

### Molecular docking

#### RAD51-BRC4 molecular docking calculations

The structure of RAD51 (PDB: 7EJC [62]) was retrieved from the Protein Data Bank [63]. Subsequently, only the ‘B’ chain was selected for further analysis, undergoing preparation using the Protein Preparation Wizard tool (Schrödinger Release 2022-1: Protein Preparation Wizard; Epik, Schrödinger, LLC, New York, NY, 2022; Impact, Schrödinger, LLC, New York, NY; Prime, Schrödinger, LLC, New York, NY, 2022). A grid was then generated, centred on the ligand ‘J46’, with dimensions appropriate for accommodating ligands of similar size. Docking of both *RS*-35d enantiomers (*S*-35d and *R*-35d), pre-processed with the Ligprep tool (Schrödinger Release 2022-1: LigPrep, Schrödinger, LLC, New York, NY, 2022), was conducted utilising Glide (Schrödinger Release 2022-1: Glide, Schrödinger, LLC, New York, NY, 2022) in XP mode. To assess potential conformational changes in the protein induced by the ligand, Induced Fit Docking (Schrödinger Release 2022-1: Induced Fit Docking protocol; Glide, Schrödinger, LLC, New York, NY, 2022; Prime, Schrödinger, LLC, New York, NY, 2022) was used, employing a standardised protocol and a redocking in XP mode for both *RS*-35d enantiomers.

#### ATM, ATR and DNA-PK molecular docking calculations

The Protein Data Bank was queried for the retrieval of the following PDB files: 7SIC, 7NI6, 7NI5, and 7NI4 for ATM; 5YZ0 for ATR; and 6ZHA for DNA-PK. The kinase domain of chain A was selected for each protein. To facilitate a rapid comparison of molecular docking results, all structures were aligned to 7NI4. Utilising the Protein Preparation Wizard tool, structures underwent a comprehensive preparation process, involving the removal of water molecules, elimination of ions, addition of hydrogen atoms, optimisation of histidine residue protonation states, refinement of the protein’s hydrogen bond network, and a restrained minimisation allowing for the free minimisation of hydrogen atoms while permitting ample movement of heavy atoms to alleviate strained bonds, angles, and clashes. For structures featuring a bound inhibitor (7NI5, 7NI4), a grid was built with Glide centred on the inhibitor, sized to accommodate the docking of ligands similar in dimensions to the bound inhibitors. In the cases of 7SIC and 7NI6, the grid was centred above ANP and ATP, respectively, with dimensions suitable for docking ligands up to 20 Å. For 5YZ0 and 6ZHA, the coordinates 193.201, 245.405, 233.298 were fixed as the grid centre, and the chosen size allowed docking of ligands up to 20 Å. The docking of both *RS*-35d enantiomers, prepared with the LigPrep tool, was conducted using Glide in SP mode. Induced fit docking with Glide was performed in standard mode, utilising standard precision (SP) for redocking. All computational procedures were executed within the Schrödinger 2022-1 suite. Image preparation was accomplished using Pymol 2.5.0 (Pymol 2.5.0, Schrödinger, LLC).

### Enantiomers separation

*R*-35d and *S*-35d enantiomers separation from the *RS*-35d racemate was carried out by the certified and validated external service Reach Separations SAS (Strasbourg, France). *RS*-35d was dissolved to 8.33 mg/mL in 30 mL acetone at 50 °C and was then purified by supercritical fluid chromatography [column details: ChiralPak IC (30 × 250 mm 5 μm), column temperature: 40 °C, flow rate: 150 mL/min, back pressure regulator: 100 BarG, detector wavelength 236 nm, injection volume: 1200 μL (10 mg), isocratic conditions: MeOH:EtOH:iPOH(1:1:1):CO_2_ 40% (0.2%% v/v isopropylamine)].

Combined fractions containing enriched *R*-35d and *S*-35d were concentrated and each re-purified individually under the same chromatographic conditions. Combined fractions of each of polish *R*-35d (first-eluted fraction, chemical purity 100%, m/z 486.1, 100% ee) and *S*-35d (second-eluted fraction, chemical purity 98.65%, m/z 486.2, 100% ee) were then evaporated to near dryness using a rotary evaporator, dissolved in acetone then evaporated to dryness at 40 °C. The resultant solids were then transferred into final vessels which was removed under a stream of N_2_ gas at 35 °C before being stored in a vacuum oven at 30 °C and 20 mbar until constant weight to afford polish *R*-35d and *S*-35d as yellow/orange powder.

*Chiral Purity Analysis Conditions* [column details: ChiralPak IC-3 (4.6 × 100mm 3 μm), column temperature: 40 °C, flow rate: 3 mL/min, detector wavelength: (210-400 nm 254 nm), injection volume: 1.0 μL, isocratic conditions: MeOH:EtOH:iPOH(1:1:1):CO_2_ 40% (0.2% v/v isopropylamine)].

*Chemical Purity Analysis Conditions* [column details: Poroshell CS-C18 (2.1 × 50 mm, 2.7 μm), column temperature: 60°C, flow rate: 1.0 mL/min, detector wavelength: 200–500 nm, injection volume: 1.0 μL, mobile phases: A water, B EtOH, C water with 10% formic acid. Linear gradient: 0–0.05 min, 2% B and 2% C; 0.05–3.0 min, 98% B and 2% C; 3.0–5.0 min, 98% B and 2% C].

### Stereochemical characterisation of *RS*-35d

The stereochemical characterisation of *RS*-35d enantiomers was performed by means of a combination of experimental circular dichroism (CD) spectroscopy and density functional theory (DFT) calculations, following a well-established protocol [64, 65]. A preliminary conformational search was carried out on two tautomeric structures of *S*-35d (Supplementary Fig. S2a, b) by molecular mechanics (MM) calculations with the RDKit software (version 2020.03.1), using the ETKDG search method (2000 initial conformers, 200 attempts) and the MMFF94 force field for energy minimisation (2000 maximum iterations, 0.01 Å RMSD clustering threshold). All 36 conformers within a MM energy window of 20 kcal mol^−1^ from the minimum (Supplementary Table S1) were used for geometry optimisation and frequency calculations at the DFT level, using the B97D3 functional [66], the def2-TZVP basis set [67] with the density fitting approximation and the IEFPCM solvation model for methanol. No conformers displayed imaginary frequencies or converged to the same geometry (identified by RMSD values for heavy atoms lower than 0.01 Å). 12 optimised conformers within an electronic energy window of 2.5 kcal mol^−1^ from the minimum (Supplementary Table S1) were further used for time-dependent DFT (TD-DFT) calculations, using the PBE0-^1^/_3_ functional [68], the def2-TZVPD basis set [69] and the IEFPCM solvation model for methanol. Excitation wavelengths (*λ*_*j*_), oscillator strengths (*f*_*j*_) and rotational strengths (*R*_*j*_, in dipole length formalism) were calculated for the first 50 excited states (Supplementary Table S2). The theoretical UV and CD spectra of *S*-35d were then derived by approximation of *f*_*j*_ and *R*_*j*_ values to Gaussian bands (Δ*σ* = 0.2 eV), summation over all excited states and Boltzmann conformational averaging based on relative electronic energies (Δ*E*_SCF_). The theoretical UV and CD spectra of *R*-35d were derived by reflection across the *x*-axis (Supplementary Fig. S1c). All DFT and TD-DFT calculations were performed using the Gaussian 16 software package (Rev. B.01) (Gaussian, Inc., Wallingford CT, 2016). The experimental UV and CD spectra of the first-eluted enantiomeric fraction of *RS*-35d were recorded using a Jasco J-715 spectropolarimeter and a 1 mm Suprasil quartz cell (Hellma Analytics). Measurements were carried out in the 500–200 nm spectral region on samples dissolved in methanol at two concentrations, namely 0.5 mg/mL (500–270 nm) and 0.1 mg/mL (300–200 nm), using a 1 nm spectral bandwidth, a 100 nm/min scanning speed, a 0.5 s data integration time, a 0.5 nm data pitch and an accumulation cycle of 3 scans per measurement. Spectra were corrected for blank and baseline drift, then converted to molar units (*ε* and Δ*ε*, in M^−1^ cm^−1^) and compared to the theoretical spectra of both *RS*-35d enantiomers (Supplementary Fig. S1c).

### ELISA assay

Competitive ELISA screening assay using biotinylated BRC4 peptide to disrupt the BRC4–RAD51 interaction was performed as described in [14]. BRC4-biotinylated peptide-coated 384-well plates (Nunc) were washed with 0.05% Tween-20 1X PBS (PBS-T) and blocked with 1% BSA/PBS-T solution, followed by overnight hybridisation with human RAD51 protein (NP_002866 Creative Biomart, NY). Compounds were added in dose response (0.01–100 μM) in triplicate with constant DMSO 1% (BRC4 and RAD51 included as positive control). Staining was performed with primary anti-RAD51 and HRP-secondary antibodies. Assay readout was developed with 3,3′,5,5′-tetramethylbenzidine signal (Sigma Aldrich) quenched with 1 M HCl. Colorimetric measure was read on a Victor5 (PerkinElmer) plate reader. Results were analysed by using GraphPad software.

### Expression and purification of His-hRAD51

Recombinant histidine-tagged human RAD51 (His-hRAD5)1 was expressed and purified as previously reported [14]. hRAD51 was expressed in *E. coli* Rosetta2(DE3)pLysS cells. A saturated overnight culture of Rosetta2(DE3)pLysS/pET15b-His-hRAD51 was diluted (1:1000) into a fresh TB-5052 autoinduction medium containing ampicillin (100 μg/mL). The flasks were shaken at 200 rpm at 20 °C for 72 h. The pellet was subsequently resuspended in an appropriate volume of buffer A (20 mM Tris-HCl (pH 8.00), 500 mM NaCl, 10 mM imidazole, 2 mM DTT, 10% (v/v) glycerol) supplemented with protease inhibitor cocktail (SIGMA-FAST protease inhibitor cocktail tablets, EDTA-50 free). The cell suspension was lysed on ice through sonication (24 rounds of 30 in.; amplitude 85%; Tip KE76; Bandelin Sonoplus HD2070 sonicator). The disrupted cell suspension was centrifuged for 30 min at 20,000 × *g*. The supernatant fraction was filtered with a 0.45 μm (MiniSart syringe filter 0.45 μm) membrane to remove residual particulates before chromatography. The supernatant was applied onto a HisTrap HP chromatography column (Cytiva), equilibrated with buffer A. A wash step was performed using 10% of buffer B (20 mM Tris-HCl (pH 8.00), 500 mM NaCl, 500 mM imidazole, 2 mM DTT, 10% (v/v) glycerol). The protein was then eluted with a linear gradient from 10% to 100% of buffer B over 10 column volumes. Fractions (0.5 mL) were collected and analysed by SDS-PAGE. Collected fractions corresponding to the recombinant protein were dialyzed overnight at 4 °C against buffer C (50 mM Tris-HCl (pH 8.00), 200 mM KCl, 0.25 mM EDTA, 2 mM DTT, 10% (v/v) glycerol). Dialyzed protein was loaded onto RESOURCE Q anion exchange chromatography column (Cytiva) equilibrated in buffer C. The elution was performed with a linear gradient of buffer D (50 mM Tris-HCl (pH 8.00), 1 M KCl, 0.25 mM EDTA, 2 mM DTT, 10% (v/v) glycerol). Fractions (0.5 mL) were collected and analysed by SDS-PAGE. Fractions containing His-hRAD51 were pooled and dialyzed against the storage buffer (20 mM HEPES (pH 8.00), 250 mM KCl, 0.1 mM EDTA, 2 mM DTT, 10% (v/v) glycerol). The protein yield was determined from the optical absorption at 280 nm (extinction coefficient 14,900 M^–1^ cm^–1^) of the final sample.

### Microscale thermophoresis

Microscale thermophoresis (MST) analyses were carried out as previously detailed [14]. The labelling of hRAD51 recombinant protein was performed with the Monolith His-Tag labelling kit RED-tris-NTA 2nd Generation kit (NanoTemper Technologies). To determine a concentration-dependent MST binding curve, MST measurements were simultaneously performed on 16 capillaries containing a constant concentration (50 nM) of labelled RED-tris-NTA 2nd Generation His-hRAD51 protein and 16 different concentrations of the compounds. The highest concentration tested was 100 μM. Measurements were carried out in MST buffer (20 mM HEPES (pH 8.00), 250 mM KCl, 0.05% (v/v) tween 20, 5% (v/v) glycerol, 5% DMSO).

### Cell lines and treatments

The human PDAC cell lines BxPC-3 (*BRCA2* wild-type), Capan-1 (*BRCA2* mutated) and the olaparib-resistant Capan-1/OP (obtained as performed in [70]) were grown in RPMI 1640 supplemented with 10% Foetal Bovine Serum (FBS), 100 U/mL penicillin/streptomycin, 2 mM glutamine. For 3D spheroid cultures, BxPC-3 and Capan-1/OP cells were seeded in sterile 1% agarose in 1X PBS-coated 96-multiwell plates and cultured for 96 h before carrying out experiments. HPAC cells were grown in RPMI 1640 supplemented with 5% FBS, 100 U/mL penicillin/streptomycin, 2 mM glutamine. The immortalised embryonic kidney cell line HEK293 was grown in DMEM high-glucose supplemented with 10% FBS, 100 U/mL penicillin/streptomycin, 2 mM glutamine. The human primary pancreatic epithelial cell line H-6037 (Cell Biologics) was grown in its specific medium (Cell Biologics, H6621) supplemented with epithelial cell growth supplement (Cell Biologics, H6621-Kit). Cell cultures were maintained at 37 °C in a humified 5% CO_2_ atmosphere. All culture media and supplements, unless otherwise specified, were from Sigma-Aldrich. All cultures were purchased from American Type Culture Collection (ATCC, Manassas, VA, USA), routinely tested for Mycoplasma contamination and authenticated through the IDEXX BioResearch (Ludwigsburg, Germany) service (Case # 63121-2018). PDAC cell line mutation status is reported in Table 2. DCFH-DA, olaparib, talazoparib, rucaparib, AZD2461, *RS*-35d, *S*-35d and *R*-35d were dissolved in DMSO at the final concentration of 10 mM. CDDP was dissolved in 1X PBS at the final concentration of 5 mM. Stock aliquots were stored at −20 °C and diluted in complete medium prior to each experiment. Treatments were administered in culture medium supplemented with 0.6% DMSO when required. The same amount of DMSO was added to the control, untreated cultures (vehicle control, CTRL).

**Table 2:** PDAC driver genes mutation status of the employed cell lines.

Cell line	*BRCA2*	*KRAS*	*TP53*	*CDKN2A*	*SMAD4*
BxPC-3	–	–	Y220C (MV)	gene del	gene del
HPAC	–	G12A (MV)	–	G120X	–
Capan-1	5946delT (FD)	G12V (MV)	A159V (MV)	–	S344X

Data obtained from [123].

*del* deletion, *FD* frameshift deletion, *MV* missense variant, *SNV* single nucleotide variant.

### Organoid cultures

PDM-41 (ATCC, HCM-CSHL-0094-C25), PDM-37 (HCM-CSHL-0090-C25) and PDM-106 (HCM-BROD-0008-C25) organoid cultures were commercially available and provided by ATCC. The patient-derived xenograft PT-127 and PT-291 organoids were previously established and characterised [37]. PDAC organoids mutation status is reported in Table 3. The Complete Human Feeding Media (CHFM) components were as follows: DMEM-F12 (Merck, D642, Darmstadt, Germany) supplemented with DMEM/F12 GlutaMAX™ Supplement (Thermo Fisher, 10565018), 1 mM HEPES (Merck, H3375), 1X Antibiotic Antimycotic (Thermo Fisher, 15240062), 50% v/v L-WRN (Wnt3a-R-spondin3 Noggin) conditioned media, 500 nM A83-01 (Sigma-Aldrich, SML0788), 100 ng/mL hFGF10 (Biolegend, 559308, San Diego, CA, USA), 50 ng/mL EGF (Thermo Fisher, PHG0311), 0.01 µM Gastrin (Tocris, 3006, Bristol, UK), 1.25 mM N-acetylcysteine (R&D, 5619, Minneapolis, MN, USA), 10 mM Nicotinamide (Sigma-Aldrich, N0636), 1X B-27 supplement (Life Technologies, 17504-044, Carlsbad, CA, USA), 10.5 µM Y-27632 ROCK inhibitor (ROCKi) (Sigma-Aldrich, Y0503). Organoids were established by resuspending cells in Cultrex Basement Membrane (BME) (R&D System Biotechne, 3432-005-01), then 20 μL of organoid/BME solution was pipetted into a 24-multiwell polyHEMA coated plate. The plate was placed at 37 °C for 15 min to allow BME polymerisation, then CHFM with 10.5 μM ROCKi was added to each well. 48 h after organoid sample establishment, cells were fed using CHFM without ROCKi, then subsequently fed every 2 days.

**Table 3:** PDAC driver genes mutation status of the employed organoids.

Organoid	*KRAS*	*TP53*	*CDKN2A*	*SMAD4*
PDM-37	G12D (MV)	K139Rfs*311 (FV)	R87_D92del	Q422X
PDM-41	G12V (MV)	P47R (MV) 599delA (FD) M237I (MV)	*29G>C (SNV)	–
PDM-106	G12V (MV)	P47R (MV)	*29G>C (SNV)	–
PT-127	G13D (MV)	P72R (MV) 599delA (FD)	*29G>C (SNV)	–
PT-291	G12D (MV)	599delA (FD) 710_711insA (I)	*29G>C (SNV)	–

Data obtained from [37].

*FD* frameshift deletion, *FV* frameshift variant, *I* insertion, *MV* missense variant, *SNV* single nucleotide variant.

### Cell viability assessment

#### 2D cells lines

Cell viability for 2D cell cultures was assessed via MTT [3-(4, 5-dimethylthiazol-2-yl)-2,5-diphenyltetrazolium bromide] assay as described in [71]. BxPC-3, Capan-1, Capan-1/OP (10^4^ cells/well), HPAC (5·10^3^ cells/well) and H-6037 (2·10^4^ cells/well) were seeded in a 96-multiwell plates and let adhere overnight. After a 72 h treatment with PARPis and *RS*-35d, *S*-35d or *R*-35d given alone or in combination, a sterile solution of 5 mg/mL MTT in 1X PBS was added to each well at the final concentration of 0.5 mg/mL. Plates were incubated at 37 °C for 4 h and formazan crystals were solubilised overnight by adding a 1:1 volume of SDS 10%/0.01 M HCl solution to each well. Absorbance was measured at 570 nm and 690 nm wavelengths on a Tecan Spark® multiplate reader.

#### 3D spheroids

MTT assay was used to assess cell viability also in 3D spheroids [72]. BxPC-3 and Capan-1/OP cells (3·10^5^ cells/well) were seeded in sterile 1% agarose in 1X PBS-coated 96-multiwell plates, grown for 96 h and treated for 72 h as previously indicated. After treatment, cell viability was assessed using the same MTT protocol described above.

#### Organoids

Organoid viability was assessed using the CellTiter-Glo® 3D Cell Viability Assay (Promega, G9681) as described in [24]. 5·10^3^ organoids in 10 μL of Cultrex BME were seeded in a 96-multiwell polyHEMA-coated black-sided, clear-bottom plate. After incubation at 37 °C for 20 min to allow BME solidification, 100 μL of CHFM, supplemented with ROCKi, was added to each well. 72 h post seeding, organoids were treated as previously indicated. After treatment, cell viability was measured using CellTiter-Glo® 3D as per the manufacturer’s instructions. The luminescence was then read with an integration of 1.0 s and gain of 100 on a plate reader (Biotek) using Gen4 software. The percentage growth was calculated relative to an untreated control.

### Interaction index calculation

The evaluation of the antineoplastic potency of drug association (*RS*-35d, *S*-35d, *R*-35d in combination with PARPis; *S*-35d and *R*-35d combination) in the employed cell lines was performed by calculating the interaction index according to the Fischel et al. adaptation of the Chou and Talalay method as previously reported [14–16]:

$${\rm{Interaction}}\,{\rm{Index}}=\frac{{\rm{Survival}}[{\rm{Drug1}}+{\rm{Drug2}}]}{{\rm{Survival}}[{\rm{Drug1}}]{\rm{Survival}}[{\rm{Drug2}}]}$$

Interaction index (i. index) values were made explicit in their respective graphs when the drug association showed synergism (i. index < 0.6: strong synergism; i. index < 0.8: synergism; 0.8 < i. index < 1.2: additive effect; i. index > 1.2: antagonism).

### Homologous Recombination Quick Assay (HR-QA)

HR was assessed by a commercially available assay from Norgen (Thorold, ON, Canada) as previously reported [14]. BxPC-3 cells (2·10^5^ cells/well) were seeded in a 24-multiwell plate and allowed to adhere overnight. The two assay plasmids were co-transfected with Lipofectamine^TM^ 2000 (Invitrogen) according to the manufacturer’s instructions. During transfection (5 h), cells were exposed to *RS*-35d, *S*-35d or *R*-35d, dissolved in RPMI in the presence of 0.6% DMSO. After washing with 1X PBS, cells were harvested, and DNA was isolated using Illustra Tissue and Cell Genomic Prep Mini Spin kit (GE Healthcare). Sample concentration was measured using an ONDA Nano Genius photometer. HR efficiency was assessed via real-time PCR, using 25 ng of template, the primer mixtures included in the assay kit and following the protocol indicated by the manufacturer. Data analysis was based on the 2^−ΔΔCt^ method: [recombination product/backbone plasmids]^treated^ versus [recombination product/backbone plasmids]^control^.

### mClover-based Homologous Recombination assay (mCL-HR)

The mClover-based Homologous Recombination assay (mCL-HR) was performed as detailed in [24]. HEK293 cells (6·10^4^ cells/well) were seeded on glass coverslips placed in a 24-multiwell plate and allowed to adhere overnight. *RS*-35d, *S*-35d or *R*-35d were added to HEK293 cells 1 h before transfection. Cells were transfected with 500 ng sgRNA plasmid targeting Lamin A (pUC CBA-SpCas9.EF1a-BFP.sgLMNA, Addgene Plasmid, #98971) and 500 ng donor plasmid (pCAGGS Donor mClover-LMNA, Addgene Plasmid, #98970), using Lipofectamine^TM^ 2000. The next day, cell culture media was replaced with fresh media containing the same compounds. 3 days after transfection, cells were fixed with a 4% paraformaldehyde (PFA) solution in 1X PBS for 15 min and washed twice with 1X PBS before mounting. Images were analysed by using the Cell Counter Plug-in of the ImageJ software (W. Rasband, Research Service Branch, National Institute of Mental Health, National Institutes of Health, Bethesda, MD and Laboratory for Optical and Computational Instrumentation, University of Wisconsin).

### Immunofluorescence

Immunofluorescence analysis for the evaluation of RAD51 nuclear translocation and DNA damage through γH2AX nuclear foci detection was performed as previously detailed [14]. For RAD51 foci detection in cell nuclei, BxPC-3 cells (2·10^5^ cells/well) were seeded on glass coverslips placed in a 6-multiwell plate and allowed to adhere overnight. Cultures were then preincubated with *RS*-35d, *S*-35d or *R*-35d for 1 h and subsequently exposed to 50 μM cisplatin for an additional 1.5 h. Medium was removed, and cells were maintained in the presence of *RS*-35d, *S*-35d or *R*-35d for 5 h. After treatment, cells were fixed with a 4% PFA 1X PBS solution for 15 min, permeabilized in 0.1% Triton^TM^ X-100 1X PBS for 15 min and washed twice with 1X PBS. Samples were incubated in 5% (w/v) bovine serum albumin (BSA) solution in 1X PBS (5% BSA/PBS) for 30 min at room temperature (RT) and subsequently stained with anti-RAD51 mouse monoclonal antibody (BioAcademia, 1:1000 in 5% BSA in PBS) overnight at 4 °C. After washing, coverslips were incubated with an anti-mouse Rhodamine-conjugated secondary antibody (1:1000 in 5% BSA/PBS) for 1 h at RT, washed, air-dried, and mounted with a solution of DAPI (2 μg/mL) and DABCO. To evaluate DNA damage through γH2AX nuclear foci, BxPC-3 and cells (2·10^5^ cells/well) were seeded on glass coverslips in 6-multiwell plate and allowed to adhere overnight. After 48 h treatment with olaparib and *RS*-35d, *S*-35d or *R*-35d given alone or in combination, cells were fixed and underwent the same protocol described above. For this experiment, a rabbit polyclonal anti-γH2AX primary antibody (Abcam, 1:1000 in 5% BSA/PBS) and an anti-rabbit Alexa Fluor™ Plus 488 secondary antibody (Thermo Fisher Scientific, 1:1000 in 5% BSA/PBS) were employed. The percentage of cells bearing nuclear foci was estimated by two independent observers, by analysing 100–250 cells for each treatment sample.

### Cytotoxicity assay

Cell death was assessed using the CellTox Green cytotoxicity assay (#G8741, Promega) as described in [14]. BxPC-3 cells (10^4^ cells/well) were seeded in a clear-bottom black 96-multiwell plate. After a 72 h treatment with olaparib and *RS*-35d, *S*-35d or *R*-35d given alone or in combination, CellTox dye was added to each well and the green fluorescence signal, produced by the binding interaction with dead cell DNA, was measured following the manufacturer’s instructions.

### Cell death assessment with vital dyes

#### 2D cell lines

Cell death assessment with vital dyes was performed as previously described [14]. BxPC-3 cells (5·10^5^ cells/well) were seeded on sterile coverslips in a 24-multiwell plate. After a 72 h treatment with olaparib and *RS*-35d, *S*-35d or *R*-35d given alone or in combination, wells were washed with PBS and filled with 500 μL of a PBS solution containing DAPI (4.6 μg/mL) and PI (50 μg/mL). After a 10 min incubation at RT under light-shielded condition, cells were washed with 1X PBS, fixed with a 4% PFA 1X PBS solution for 10 min, and washed again to eliminate the fixative excess. Coverslips were then applied on glass slides using two drops of mounting media (DABCO). The percentage of PI-positive cells was estimated by two independent observers, by analysing 100–250 cells for each treatment sample.

#### 3D spheroids

For cell death assessment with vital dyes in 3D spheroids, BxPC-3 and Capan-1/OP 3D spheroids obtained as previously described were treated as detailed above. After treatment, Calcein-AM and PI were added to each well at the final concentration of 2.5 and 3.75 µM respectively as detailed in [73]. To determine cell death rate, Calcein-AM (Ex = 485 nm, Em = 530 nm) and PI (Ex = 535 nm, Em = 620 nm) fluorescence signals were measured on a Tecan Spark® multiplate reader and results were expressed as PI/Calcein-AM ratio.

### Micronuclei visualisation

Micronuclei visualisation was carried out as previously detailed [14]. BxPC-3 cells (5·10^5^ cells/well) were seeded on sterile coverslips in a 24-multiwell plate. After a 72 h treatment with olaparib and *RS*-35d, *S*-35d or *R*-35d given alone or in combination, wells were washed with 1X PBS and fixed with ice-cold methanol for 10 min. Coverslips were air-dried and mounted on glass slides using a solution of DAPI (5 μg/mL)/DABCO. A cell was considered to contain micronuclei if the following criteria were met at the same time: (i) one or more round fluorescent bodies were present in the cytoplasm which did not touch the main nucleus; (ii) they were <1/3 of the main nucleus diameter; (iii) they were non-refractile, to exclude foreign bodies. The percentage of cells bearing micronuclei was estimated by two independent observers, by analysing 100–250 cells for each treatment sample.

### Colony formation assay

The colony formation assay was carried out as described in [74]. BxPC-3 cells (500 cells/well) were seeded in a 6-multiwell plate and let adhere overnight. After 48 h treatment with olaparib and *RS*-35d, *S*-35d or *R*-35d given alone or in combination, cells were gently washed with 1X PBS and fresh medium was added to each well. After 10 days, colonies were fixed with ice-cold methanol at −20 °C overnight, stained with a 0.5% (w/v) crystal violet (Sigma-Aldrich) solution dissolved in a 20% (v/v) methanol solution and air-dried before image acquisition. Image processing and colony counting were performed with ImageJ software using the ColonyArea plugin described in [75]. Results are expressed as colony rate and as % of vehicle controls (CTRL).

### Scratch wound-healing assay

The scratch wound-healing assay was carried out as detailed in [74]. BxPC-3 cells (5·10^5^ cells/well) were seeded in a 12-multiwell plate and grown at confluence. After performing the scratches with a sterile tip, cells were washed with 1X PBS and treated for 48 h with olaparib and *RS*-35d, *S*-35d or *R*-35d given alone or in combination. After treatment, cells were washed with 1X PBS and fixed with ice-cold methanol at −20 °C overnight, stained with a 0.5% (w/v) crystal violet (Sigma-Aldrich) solution dissolved in a 20% (v/v) methanol solution and air-dried before image acquisition. Image processing and wound area were performed with ImageJ software using the WH_NJ macro described in [76]. Results are expressed as % of the wound area at t = 0 h.

### Apoptosis analysis

#### 2D cell lines

Apoptosis evaluation was carried out by surface labelling with the Ca^2+^-dependent phosphatidylserine-binding protein Annexin V and the dead cell-permeable PI with the commercially available Dead Cell Apoptosis Kits with Annexin V for Flow Cytometry (Cat No. V13242, Invitrogen, Paisley, UK). BxPC-3 cells (7·10^5^ cells/well) were seeded in 6-multiwell plates and let adhere overnight. After a 48 h treatment with olaparib and *RS*-35d, *S*-35d or *R*-35d given alone or in combination, cells were detached with 0.05% trypsin-EDTA, centrifuged 120 g for 7 min and washed in ice-cold 1X PBS. Cells were labelled via incubation with a FITC-conjugated Annexin V antibody and PI in the provided Annexin binding buffer for 15 min at RT in the dark, according to the manufacturer’s instruction. Stained cells were analysed (10,000 events) on a BD FACSAria™ II flow cytometer (BD Biosciences). Flow cytometry data were visualised and analysed with the BD FACSDiva^TM^ software (version 9.0.1).

#### Organoids

Apoptosis evaluation in human PDAC organoids was carried out using the Caspase 3/7 apoptosis assay. Organoids were seeded as previously outlined, 72 h post seeding, the drugs were added in combination with the Caspase 3/7 Green Dye (Sartorius, 4440) was added at a concentration of 1:2000 (final assay concentration 1.25 μM). Cells were treated with 100 μL 2X drug-Caspase 3/7 green dye. The plate was placed in the Incucyte (SG, Sartorius) and imaged using the phase contrast and green fluorescence channels every 2 h over a 5-day period.

### Cell cycle analysis

Cell cycle analysis was performed following the same protocol detailed in [74]. BxPC-3 cells (5·10^5^ cells/well) were seeded in a 6 multiwell plate in complete RPMI medium and were subsequently starved for 24 h in RPMI without FBS. After 24 h of starvation, BxPC-3 cells were recovered in complete RPMI medium for 24 h and then treated with the different compounds for 24 h (a timing without visible cytotoxic effects based on preliminary screenings). After treatment, pellets were washed in ice-cold 1X PBS, fixed with ice-cold ethanol 70% dispensed drop-wise while vortexing and stored at −20 °C overnight. Pellets were stained with a 1X PBS solution containing RNase 100 μg/mL RNase-A, 0.05% (v/v) Triton X-100 and 60 μg/mL PI and incubated at 37 °C for 1 h. Samples were incubated on ice and protected from light before FACS acquisition. FACS analysis was performed on a BD FACSAria™ II flow cytometer (BD Biosciences). Data were analysed and visualised with the FlowJo software (version 10.6.2).

### Detection of intracellular reactive oxygen species (ROS)

Intracellular ROS were detected using the oxidation-sensitive DCFH-DA fluorescent probe adapting the protocol detailed in [77]. BxPC-3 cells (5·10^5^ cells/well) were seeded in a 6 multiwell plate, let adhere overnight and treated for 24 h (a timing without visible cytotoxic effects based on preliminary screenings) with the selected compounds. After treatment, cells were harvested, resuspended in 10 µM DCFH-DA in 1X PBS and incubated 45 min at 37 °C. Pellets were resuspended in DCFH-DA-free RPMI medium without FBS and cells (5·10^4^/100 µL) were dispensed in each well of a black bottom 96 multiwell plate. DCF (Ex = 485 nm, Em = 530 nm) fluorescence signal was measured on a Tecan Spark® multiplate reader. DCF signal was detected in parallel through fluorescence microscopy to corroborate the in-plate analysis.

### Mitochondrial activity assay

Treatment impact on mitochondrial efficiency was measured through ATP levels quantification as detailed in [78]. BxPC-3 cells (2·10^4^ cells/well) were seeded in a clear-bottom black 96-multiwell plate. After a 24 h treatment (a timing without visible cytotoxic effects based on preliminary screenings) with the different compounds, ATP levels were measured with CellTiter-Glo® (Promega) following the manufacturer’s instructions. Luminescence signal was measured on a Tecan Spark® multiplate reader. Cell viability in the same conditions was monitored in parallel with the non-enzymatic crystal violet assay. After 24 h treatment, cell media were removed and collected for the NMR exometabolomic analysis (described further on) and wells were washed twice with 1X PBS. Cells were then fixed with 120 µL/well of 1% (v/v) glutaraldehyde solution (in water) for 20 min, washed twice with 1X PBS and stained with 0.01% (w/v) crystal violet solution (in water) for 30 min at RT. Wells were then washed with 1X PBS until excess staining dye was removed and cell-bound crystal violet was solubilised with 100 µL/well of ice-cold 70% (v/v) ethanol for 30 min in agitation. Absorbance was then measured at 570 nm on a Tecan Spark® multiplate reader.

### NMR exometabolomic analysis

#### Sample preparation

Metabolomic studies on cell culture media to investigate treatment-induced changes on mitochondrial function and oxidative metabolism in BxPC-3 cells were performed as described in [46, 47]. Culture media of the same cells used for the mitochondrial activity assay were collected right before cell viability evaluation, immediately frozen in liquid nitrogen and stored at −80 °C. Right before NMR analysis, culture media of cells exposed to the different treatments were thawed on ice, centrifuged at 20,000 × *g* 4 °C for 15 min. 400 μL of each sample were diluted with 100 μL of a prepared NMR buffer for a final concentration of 150 mM buffer phosphate pH 7.4, 1 mM 2,2′,3,3′-deuterotrimethylsilylproprionic acid (TSP) as chemical shift reference, 0.04% sodium azide, and 10% D_2_O (for the lock signal) into a 5 mm NMR tube.

#### NMR analysis

All the NMR experiments were recorded with a Bruker Ultrashield Plus FT-NMR 600 MHz ADVANCENEO equipped with a Cryoprobe™ QCI ^1^H/^19^F–^13^C/^15^N–D with a SampleJet™ autosampler with temperature control. For each sample, the probe was automatically locked, tuned, matched, and shimmed. Before measurement, the samples were kept for 5 min inside the NMR probe head for temperature equilibration at 298 K. Two NMR spectra were recorded for each sample: a monodimensional (1D) 1H NMR spectrum with a standard pulse sequence water suppression (noesygppr1d, Bruker), with 128 scans, 64k data points, a spectral width of 30 ppm, an acquisition time of 1.835 s, a relaxation delay of 4 s, and a mixing time of 100 ms and a 1D 1H spin-echo Carr-Purcell-Meiboom-Gill sequence [79, 80] (cpmgpr1d, Bruker) to suppress large NMR signals arising from high molecular weight molecules (i.e. serum proteins). In the cpmg experiments, the total echo time was to 38.4 ms consisting of 128 repetitions with a τ time of 300 μs and a 180° pulse of ~36 μs. Each spectrum was recorded with a total of 128 scans, 64k data points, a repetition time of 4 s, and an acquisition time of 1.835 s. The free induction decay was multiplied with an exponential window function with 0.3 Hz line broadening prior to Fourier transformation. All the 1 H NMR chemical shifts are referenced to the TSP signal.

#### Metabolomic analysis

Obtained spectra were analysed MetRENova 15.01 Chemometris package from spectra processing, bucketing, and normalisation to statistical principal component analysis (PCA). For bucketing, a width of 0.04 ppm was used, and the samples were normalised based on the total intensity (each bucket integration is divided by the integration of the total spectrum). The significant NMR buckets, resulted from MetRENova, were assigned by Assure 2.2 Bruker programme, the Human Metabolome Database (https://hmdb.ca/) and Chenomx Profiler (Chenomx NMR suite 8.5 evaluation). The identification and quantification of 29 different metabolites was obtained automatically by Assure 2.2, using an external standard (10 mM dimethyl malonic acid), and the PULCON method [81]. The consumption or the production/release of each metabolite was calculated by comparing their amounts with those in the procedural blank (medium incubated in the same experimental conditions but without cells). Only significant (p < 0.05 as reference value, one-way ANOVA followed by Bonferroni’s post hoc test) consumption/release values were included in the respective figure.

### Protein extraction and immunoblot analysis

Protein extraction and immunoblot analysis was performed as detailed in [82, 83]. After treatment, cells were scraped in ice-cold 1X PBS, centrifuged at 13,000 × *g* for 5 min and lysed in 100 µL of homogenisation buffer (50 mM Tris−HCl pH 7.5, 150 mM NaCl, 5 mM EDTA, 0.5% Triton X-100 and proteases and phosphatases inhibitor mix). Protein content was assessed via the Bradford method using BSA as a calibration standard. Immunoblot samples were prepared mixing the cell lysate with sample buffer (125 mM Tris−HCl pH 6, 8.4% SDS, 20% glycerol, 6% β-mercaptoethanol, 0.1% bromophenol) and denaturing at 95 °C for 5 min. Equivalent amounts of extracted protein were electrophoresed into an appropriate % SDS-PAGE under reducing conditions. The proteins were then transferred onto a nitrocellulose membrane (Amersham, Little Chalfont, UK) that was blocked in 5% (w/v) non-fat milk, 1X TBS, 0.1% Tween-20 for 1 h with gentle shaking. Proteins were visualised using primary antibodies diluted in 5% w/v non-fat milk, 1X TBS, 0.1% Tween-20 for HIF-1α (1:1000), GLUT1 (1:1000), CDH1 (1:1000), β-tubulin (1:1000), p-CHK1 (1:1000), p-CHK2 (1:1000), p-Akt (1:1000), FANCI (1:1000), FANCD2 (1:5000), RPA3 (1:200) and β-actin (1:2000). Immunoreactivity was measured with host-specific secondary IgG peroxidase-conjugated antibodies (1:5000 diluted). Chemiluminescence was developed with the long-lasting chemiluminescent substrate LiteAblot® EXTEND (Cat. N° EMP013001, EuroClone, Milan, Italy). For signal detection ChemiDoc^TM^ MP Imaging System (BioRad) was used. After Western blot acquisition, bands optical analysis was performed with the ImageJ software. Bands relative densities were expressed as arbitrary units and normalised over control sample run under the same conditions.

### Real-time PCR

Real-time PCR was performed as described in [84, 85]. Total RNA was extracted using RNeasy Plus Mini Kit (Qiagen, Valencia, CA, USA), following the manufacturer instructions and RNA quantification was performed with NanoDrop 2000™ (Thermo Fisher Scientific, Rockford, IL, USA). 1 µg of total RNA was retrotranscribed into first-strand cDNA by using the Super-Scripts® VILO™ cDNA Synthesis Kit (Thermo Fisher Scientific). Real-time PCR was performed with ViiA™ 7 Real-Time PCR System (Applied Biosystems) using universal cycling conditions (95 °C, 10 min; 95 °C, 15 s; and 60 °C, 1 min for 40 cycles). PCR reactions were performed in duplicate, according to the standard protocols suggested by the manufacturer. For each PCR reaction, 25 ng total RNA was used. GAPDH mRNA was used as an endogenous reference. Transcript quantification was performed with 2^−ΔΔCt^ method [86]. Primers sequences of the human genes analysed are listed in Table 4.

**Table 4:** Primers employed for RT-PCR analyses.

Gene	Forward primer	Reverse primer	Reference
*GAPDH*	AAGGTGAAGGTCGGAGTCAA	AATGAAGGGGTCATTGATGG	[124]
*HIF1A*	GTGGAAGTGGCAACTGATGA	ATTCACCATGGAGGGCG	[35]
*GLUT1*	AGCCTGTGTATGCCACCATT	GCCCACAATGAAATTTGAGG	[35]
*CDH1*	GCAGGAGAGCTTGTCATTGAC	AGACTCCTCCATTCCTTCCAG	[125]

### Kinase activity profiling

The evaluation of *RS*-35d, *R*-35d and *S*-35d inhibition on the panel of selected kinases was carried out with the KinaseProfiler™ service at Eurofins Cerep SA. Specifically, kinase enzymatic ELISA/EIA [*K*_m_ ATP] was employed for ATM, ATR/ATRIP, DNA-PK, radiometric [*K*_m_ ATP] for CDK2, CHK1, CHK2, CK2, Akt, PLK1, WEE1 and Time-resolved fluorescence energy transfer (TR-FRET) [*K*_m_ ATP] for PI3K.

#### Kinase enzymatic ELISA/EIA [K_m_ ATP]

ATM, ATR or DNA-PK were incubated with ATM buffer (25 mM HEPES pH 8.0, 0.01% Brij-35, 1% Glycerol, 10 μM ATP, 5 mM MgAc, 5 mM MnCl_2_), ATR buffer (25 mM HEPES pH 8.0, 0.01% Brij-35, 1% Glycerol, 10 μM ATP, 10 mM MnCl_2_ and 50 nM) or DNA-PK buffer (Mg/ATP) respectively with 50 nM GST-cMyc-p53 as a substrate. The reaction was initiated with the addition of ATP. After incubation for 30 min RT, the reaction was stopped by the addition of stop solution containing EDTA. Finally, detection buffer was added, which contains d2-labelled anti-GST monoclonal antibody, and a Europium-labelled anti-phospho Ser15 antibody against phosphorylated p53. The plates were then read in time-resolved fluorescence (TRF) mode. The homogeneous time-resolved fluorescence (HTRF) signal was determined according to the formula HTRF = 10,000 × (Em 665 nm/Em 620 nm).

#### Kinase enzymatic radiometric [K_m_ ATP]

CDK2, CHK1, CHK2, PKB, PLK1 were incubated with 8 mM MOPS pH 7.0, 0.2 mM EDTA, 10 mM Magnesium acetate. CK2 was incubated with 20 mM HEPES pH 7.6, 0.15 M NaCl, 0.1 mM EDTA, 5 mM DTT, 0.1% Triton X-100. WEE1 was incubated with 20 mM Tris/HCl pH 8.5, 0.2 mM EDTA. The respective substrates were added to each assays, the reaction was initiated by the addition of Mg/[^33^P- γ]-ATP mix. After incubation for 40 min RT, the reaction was stopped by the addition of phosphoric acid to a concentration of 0.5%. An aliquot of the reaction is then spotted onto a filter and washed four times for 4 min in 0.425% phosphoric acid and once in methanol prior to drying and scintillation counting.

#### Kinase TR-FRET [K_m_ ATP]

PI3K was incubated in assay buffer containing 10 μM phosphatidylinositol 4,5-bisphosphate and Mg/ATP. The reaction was initiated by the addition of the ATP solution. After incubation for 30 min RT, the reaction was stopped by the addition of stop solution containing EDTA and biotinylated phosphatidylinositol- 3,4,5-trisphosphate. Finally, detection buffer was added, which contained europium-labelled anti-GST monoclonal antibody, GST-tagged GRP1 PH domain and streptavidin allophycocyanin. The plate was then read in TRF mode and the HTRF signal was determined according to the formula HTRF = 10,000 × (Em 665 nm/Em 620 nm).

### Fluorescence and brightfield imaging

Fluorescence images were taken using a Leica DFC360 FX camera through a Leica DMI6000 B inverted microscope (Leica Microsystems, Wetzlar, Germany) equipped with filters for FITC, TRITC and DAPI (scale bars were added using the ImageJ software). Brightfield imaging of 3D spheroids was performed at 0, 24, 48 and 72 h treatment timings to determine spheroid volume change. Pictures were taken using a Leica DFC360 FX camera through a Leica DMI6000 B inverted microscope (scale bars were added using the ImageJ software). Image processing was performed with ImageJ software using the SA_NJ macro described in [76]. Spheroid volume was calculated using the general formula described in [87]:

$${\rm{Spheroid}}\; {\rm{Volume}}\left({\rm{V}}\right)=0.5\cdot \left({\rm{Length}}\right)\cdot {({\rm{Width}})}^{2}$$

### Whole-cell lysate MS-based proteomic analyses

Proteomic analyses on whole-cell lysates were carried out as described in [13]. BxPC-3 cells (5·10^6^ cells/T75 flask) were cultured for 2 days, washed with PBS and treated with 20 µM *RS*-35d or DMSO (as vehicle control) for 24 h. After the treatment, cells were harvested, washed twice with cold PBS and stored at −80 °C. BxPC-3 cells were lysed in whole-cell lysis buffer (25 mM Tris·HCl pH 7.6, 150 mM NaCl, 1% NP-40, 1% sodium deoxycholate, 0.1% SDS, 0.5 mM DTT) supplemented with 1X complete protease inhibitor cocktail tablets (Sigma-Aldrich). Lysates were incubated for 15 min on ice, sonicated for 2 s at high power, incubated on ice for another 15 min and cleared by centrifugation at 15,000 × *g* for 15 min at 4 °C. Supernatant was recovered and transferred into a new tube. Samples were stored at −80 °C until further use. Protein concentration of cell lysates was assessed via the Pierce 660-nm Protein Assay (Thermo Fisher Scientific) following the manufacturer instruction/protocol. For each sample, 50 μg of proteins were diluted in 20 μL of buffer/MS grade water. Samples were homogenised and denatured in urea (final concentration, 4 M), ammonium bicarbonate (100 mM), and calcium chloride (100 mM), then reduced in DTT (final concentration, 1 mM) for 15 min at room temperature (RT) and alkylate in iodoacetamide (IAA) (3 mM) in the dark for 15 min at RT. Tryptic digestion protocol was performed using automated KingFisher DuoPrime purification system in a series of steps (Thermo Fisher) described in [88] with minor adjustments. A combination of magnetic hydrophobic and hydrophilic beads were washed multiple times in MS grade water and added to the deep-well plate in the KingFisher along with the samples and the same volume as the sample of 100% ethanol. Next, solutions were mixed at low speed for 10 min, then the protein-coupled beads were collected with the magnetic arm of the KingFisher and transferred to be washed in 3 different deep-well plates each containing 80% of ethanol. The washed beads-proteins were released into trypsin (Promega, V5111)-containing deep-wells at a 50:1 (w/w) protein to protease ratio and mixed at low speed for 4 h of digestions into peptide fragments at 37 °C in the KingFisher. Peptide samples were transferred into low protein binding tubes, 1% of trifluoroacetic acid (TFA) was added to acidify the samples ready to be desalted, cleaned, and concentrated on C18Tips (Thermo Fisher, 87,784) [89]. Purified peptides were dried and stored at −80 °C prior to use.

### Mass spectrometry analysis

MS analysis was carried out as described in [13]. Three biological samples were prepared for each condition, and each biological sample was further analysed once more in full-process technical replicate (×2). Samples were run on a Bruker timsTOF Pro mass spectrometer (Bruker Daltonics) connected to a Bruker nanoElute nano-LC chromatography system (Bruker Daltonics) as previously described [13]. Tryptic peptides were resuspended in LC-MS water with 0.1% (v/v) trifluoroacetic acid (TFA). Each sample was loaded onto an Acclaim PepMap C18 trap cartridge (0.3 mm inside diameter, 5 mm length, Part 160434, Thermo Fisher Scientific) at an approximate flow rate of 10 μL/min with buffer A (99.9% water, 0.1% formic acid). Peptides were then separated on an Aurora UHPLC column (25 cm × 75 μm ID, C18, 1.6 μm) (Ionopticks) over 60 min. Separation was carried out with a linear gradient: from 0 to 53 min buffer B (99.9% acetonitrile, 0.1% TFA) increases from 2 to 32% at a flow rate of 250 nL/min, from 53 to 55 min buffer B increases from 32 to 95%, from 55 to 60 min the column is washed with 95% buffer B. The mass spectrometer, Bruker timsTOF Pro was operated in positive ion mode with a capillary voltage of 1500 V, dry gas flow of 3 L/min and a dry temperature of 180 °C. All data was acquired with the instrument operating in trapped ion mobility spectrometry (TIMS) mode. Trapped ions were selected for ms/ms using parallel accumulation serial fragmentation (PASEF). A scan range of (100–1700 m/z) was performed at a rate of 10 PASEF MS/MS frames to 1 MS scan with a cycle time of 1.15 s. The data analysis was done using PEAKS X+ (PEAKS Studio/Q/IMS, Bioinformatics Solutions Inc., Waterloo, Ontario, Canada). In terms of proteomic depth and data management, for each biological sample the relative technical replicates were combined averaging both protein ion abundance values. Database searches were performed using default parameters: a parent mass tolerance of 20 ppm; a fragment tolerance of 0.5 Da; Carbamidomethyl (C) was specified as fixed modifications, Oxidation (M) was specified as dynamic modification, and acetylation was specified as N-terminal modification. Trypsin/P digest enzyme (maximum 3 missed cleavages, or nonspecific cleavages allowed), de novo sequencing and the match-between-runs feature were enabled according the default settings [13]. PEAKS database searching was performed against the human proteome (UniprotKB/SwissProt human protein sequence database UP000005640, reviewed protein only). FDR was estimated with target decoy fusion and set to 0.01. Label-free quantification with PEAKS Q was used. PEAKS was allowed to autodetect the reference sample and automatically align the sample runs [13]. To improve the quality of analysis results, the software further filtered the retrieval results: combining the identified protein (contained at least 1 unique peptide) and subsequently contaminant was removed. Protein table are listed in the Supplementary Info excel tables: Protein Group. For MS data visualisation, volcano plot was generated using Origin Software (OriginPro 2020). RAD51, ATM, ATR, and DNA-PKcs interactomes (homo sapiens) were calculated using the online free database BioGRID (version 4.4.214) [90], last access on January 2024 and visualised using FunRich (version 3.1.3). Protein network analysis was conducted using the free online software STRING (version 11.5) [13].

### TPGS-based nanoparticles preparation and characterisation

In a 20 mL glass vial, 2 mL of a 1.5 mg/mL *RS*-35d solution in acetone were dispensed in 10 mL of a 0.3 mg/mL TPGS solution in deionized water in two fast shot using a 1 mL pasteur pipette, stirring (PTFE-coated, cylindrical magnetic stirring bar, L 10 mm, Ø 3 mm) at 500 rpm for 10 min. The mixture was then kept at 45 °C for 2 h under a flow of argon to ensure acetone evaporation. 90 mg of solid NaCl were added to reach the physiological concentration of 0.9% (w/v), and the resulting mixture was sonicated for 5 min (Bandelin Sonoplus HD2070 tip sonicator; amplitude 52%; Tip MS72). The dispersion was then sterilised via exposure to germicidal UV lamp (253.7 nm wavelength) for 20 min and directly analysed via dynamic light scattering (DLS) using a Zetasizer Nano (Malvern, Worcestershire, U.K.) equipped with a 4.5 mW laser diode and operating at 670 nm, with a constant scattering angle of 173°, finally obtaining Z-average hydrodynamic size, polydispersity index (PDI) as a measure of the width of the size distribution, and ζ-potential [91].

### Statistical analysis

Each experiment consists of at least three independent experiments. Data are presented as means ± standard deviation (SD). Statistical analyses were conducted with GraphPad Prism version 10 (GraphPad Software, San Diego, CA, USA) and outliers were determined with GraphPad outlier calculator (alpha = 0.05). Statistical differences were determined by analysis of variance (one-way or two-way ANOVA) followed, when significant, by Dunnett’s or Tukey’s multiple comparison post hoc tests. In all reported statistical analyses, effects were designated as significant if the *p*-value < 0.05. For the proteomic analysis, the statistical significance was calculated with the criterion of *p*-value < 0.05 and log_2_(fold change) > 1 or < −1. The gene ontology (GO) analysis of the differentially regulated proteins were carried out through the gene ontology visualisation tool EnrichR [92] (the top 10 significant proteins were analysed and sorted according to their *p*-value).

## Supplementary information

Supporting Material—Supplementary Data

Supporting Information—Uncropped Immunoblot Images

Supporting Information—Proteomic Profiling

## Data Availability

The MS proteomics data have been deposited to the ProteomeXchange Consortium via the PRIDE partner repository with the dataset identifier PXD054120.
