# Supplementary material for: Investigating synthetic lethality and PARP inhibitor resistance in pancreatic cancer through enantiomer differential activity
Source: Cell Death Discov. 2025 Mar 16;11:106. doi: 10.1038/s41420-025-02382-3 (PMC11911456; doi:10.1038/s41420-025-02382-3)
Supplement: Supplementary file 1 — Supporting Material—Supplementary Data [file 41420_2025_2382_MOESM1_ESM.docx]

**SUPPLEMENTARY INFORMATION**

**Investigating Synthetic Lethality and PARP Inhibitor Resistance in Pancreatic Cancer Through Enantiomer Differential Activity**

Mirco Masi^1,#^, Laura Poppi^2,#^, Viola Previtali^1,#^, Shannon R Nelson^3^, Kieran Wynne^4,5^, Giulia Varignani^1^, Federico Falchi^1,2^, Marina Veronesi^6^, Ennio Albanesi^7^, Daniele Tedesco^8^, Francesca De Franco^9^, Andrea Ciamarone^1,2^, Samuel H Myers^1^, Jose Antonio Ortega^1^, Greta Bagnolini^2^, Giovanni Ferrandi^1,2^, Fulvia Farabegoli^2^, Nicola Tirelli^10^, Giuseppina Di Stefano^11^, Giorgio Oliviero^4^, Naomi Walsh^3^, Marinella Roberti^2^, Stefania Girotto^1,6,*^, Andrea Cavalli^1,2,12,*^

1 Computational and Chemical Biology, Italian Institute of Technology IIT, via Morego 30, 16163 Genoa, Italy

2 Department of Pharmacy and Biotechnology, University of Bologna, Via Belmeloro 6, 40126 Bologna, Italy

3 National Institute for Cellular Biotechnology, School of Biotechnology, Dublin City University, Dublin 9, Ireland

4 Systems Biology Ireland, School of Medicine, University College Dublin, Belfield Dublin 4, Ireland

5 Conway Institute of Biomolecular & Biomedical Research, University College Dublin, Dublin 4, Ireland.

6 Structural Biophysics Facility, Italian Institute of Technology IIT, via Morego 30, 16163 Genoa, Italy

7 Department of Neuroscience and Brain Technologies, Neurofacility, Italian Institute of Technology IIT, via Morego 30, 16163, Genoa, Italy.

8 Institute for Organic Synthesis and Photoreactivity (ISOF), National Research Council of Italy (CNR), via P. Gobetti 101, I-40129 Bologna, Italy

9 TES Pharma S.r.l., Via Palmiro Togliatti 22bis, I-06073 Corciano, Perugia, Italy

10 Laboratory for Polymers and Biomaterials, Italian Institute of Technology IIT, via Morego 30, 16163 Genoa, Italy

11 Department of Surgical and Medical Sciences, University of Bologna, 40126 Bologna, Italy

12 Centre Européen de Calcul Atomique et Moléculaire (CECAM), Ecole Polytechnique Fédérale de Lausanne, Lausanne, Switzerland.

# These authors contributed equally;

* These authors contributed equally; Correspondence: andrea.cavalli@iit.it; stefania.girotto@iit.it

**Table of Contents**

Supplementary Table S1. Conformational search on the low-energy conformers of *S*-35d 1

Supplementary Table S2. Rotational strengths of *S*-35d conformers 2

Supplementary Figure S1. *RS*-35d enantiomers separation through supercritical fluid chromatography4

Supplementary Figure S2. Stereochemical characterisation of *RS*-35d5

Supplementary Figure S3. Enantiomers differential inhibiting activity towards RAD51-BRCA26

Supplementary Figure S4. Molecular docking for *S*-35d and *R*-35d in RAD51 structure and MST traces of *RS*-35d, *S*-35d and *R*-35d binding to His-hRAD517

Supplementary Figure S5. Complete cell viability graphs reporting all tested concentrations of RS-35d, S-35d and R-35d in BxPC-3, HPAC, Capan-1 and H-6037 cell lines8

Supplementary Figure S6. Gating strategy used in flow cytometry for apoptosis analysis9

Supplementary Figure S7. Characterisation of tumour-mimicking BxPC-3 3D spheroids and optimisation of single-agent treatments in human PDAC organoids10

Supplementary Figure S8. Characterisation of *S*-35d+*R*-35d reconstituted racemic mixture11

Supplementary Figure S9. Molecular docking of *RS*-35d enantiomers in ATM structure12

Supplementary Figure S10. Molecular docking of *RS*-35d enantiomers in ATR structure13

Supplementary Figure S11. Molecular docking of *RS*-35d enantiomers in DNA-PK structure14

Supplementary Figure S12. Further dissection of *RS*-35d mechanism of action15

Supplementary Figure S13. *RS*-35d proteomic profile in BxPC-3 cells16

Supplementary Figure S14. Establishment of olaparib-resistant Capan-1/OP cell line and characterisation of tumour-mimicking Capan-1/OP 3D spheroids17

Supplementary Figure S15. *RS*-35d NPs characterisation and effect on *BRCA2*-proficient and *BRCA2*-mutated, PARPi resistant PDAC cells18

**Supplementary Table S1.** Results of the conformational search on the low-energy conformers of *S*-35d: absolute MM energies (E_MM_) at the MMFF94 level; absolute and relative DFT electronic energies (*E*_SCF_ and Δ*E*_SCF_), Boltzmann populations based on electronic energies (*χ*_SCF_) and absolute free energies (*G*), as obtained after DFT optimization at the B97D3/def2-TZVP/fit level (IEFPCM solvation model for methanol); [*α*]_D_ values as obtained by TD-DFT calculations at the PBE0-⅓/def2-TZVPD//B97D3/def2-TZVP/fit level (IEFPCM solvation model for methanol).

| **Conf. ID** | **Δ*E*_MM_ (kcal mol^−1^)** | ***E*_SCF_ (Ha)** | **Δ*E*_SCF_ (kcal mol^−1^)** | ***χ*_SCF_ (%)** | ***G* (Ha)** | **[*α*]_D_ (deg)** |
| --- | --- | --- | --- | --- | --- | --- |
| ***S*-35d.T1c** | 76.1680 | **−1931.2531635** | **0.0000** | 26.301 | −1930.863561 | −148.87 |
| ***S*-35d.T1d** | 76.1766 | −1931.2529199 | 0.1529 | 20.320 | −1930.863289 | −198.61 |
| ***S*-35d.T1a** | **75.5215** | −1931.2524124 | 0.4713 | 11.871 | −1930.863292 | −331.62 |
| ***S*-35d.T1b** | 75.5217 | −1931.2521714 | 0.6226 | 9.197 | −1930.864147 | −430.23 |
| ***S*-35d.T1e** | 76.5141 | −1931.2521686 | 0.6243 | 9.170 | −1930.863301 | −270.57 |
| ***S*-35d.T1f** | 76.5151 | −1931.2519620 | 0.7540 | 7.367 | −1930.862897 | −354.51 |
| ***S*-35d.T1j** | 77.4396 | −1931.2510266 | 1.3409 | 2.736 | −1930.863474 | 296.09 |
| ***S*-35d.T1i** | 77.3846 | −1931.2509866 | 1.3660 | 2.622 | −1930.863043 | 278.82 |
| ***S*-35d.T1l** | 77.9507 | −1931.2508659 | 1.4418 | 2.308 | −1930.862225 | 523.67 |
| ***S*-35d.T1h** | 76.5634 | −1931.2508538 | 1.4494 | 2.278 | −1930.864126 | 478.16 |
| ***S*-35d.T1k** | 77.8384 | −1931.2508046 | 1.4802 | 2.162 | −1930.862315 | 534.30 |
| ***S*-35d.T1g** | 76.5175 | −1931.2507511 | 1.5138 | 2.043 | −1930.864561 | 448.22 |
| ***S*-35d.T1q** | 85.1116 | −1931.2492930 | 2.4288 | 0.436 | −1930.859448 |  |
| ***S*-35d.T1s** | 85.4901 | −1931.2491088 | 2.5444 | 0.359 | −1930.858310 |  |
| ***S*-35d.T1m** | 83.4551 | −1931.2488080 | 2.7331 | 0.261 | −1930.860243 |  |
| ***S*-35d.T1n** | 83.8464 | −1931.2485059 | 2.9227 | 0.190 | −1930.860393 |  |
| ***S*-35d.T1o** | 84.7821 | −1931.2482929 | 3.0563 | 0.151 | −1930.859247 |  |
| ***S*-35d.T1r** | 85.1958 | −1931.2479929 | 3.2446 | 0.110 | −1930.858839 |  |
| ***S*-35d.T1x** | 87.3846 | −1931.2467428 | 4.0291 | 0.029 | −1930.857743 |  |
| ***S*-35d.T1v** | 86.5882 | −1931.2466640 | 4.0785 | 0.027 | −1930.857798 |  |
| ***S*-35d.T1t** | 85.8594 | −1931.2463015 | 4.3060 | 0.018 | −1930.859651 |  |
| ***S*-35d.T1p** | 85.0632 | −1931.2462757 | 4.3222 | 0.018 | −1930.859510 |  |
| ***S*-35d.T1w** | 87.3015 | −1931.2460006 | 4.4948 | 0.013 | −1930.857645 |  |
| ***S*-35d.T1u** | 86.4831 | −1931.2459545 | 4.5237 | 0.013 | −1930.857971 |  |
| ***S*-35d.T2c** | 87.4102 | −1931.2396399 | 8.4862 | 0.000 | −1930.850195 |  |
| ***S*-35d.T2d** | 87.4542 | −1931.2393874 | 8.6446 | 0.000 | −1930.850195 |  |
| ***S*-35d.T2a** | 86.7414 | −1931.2387913 | 9.0187 | 0.000 | −1930.850855 |  |
| ***S*-35d.T2e** | 87.7279 | −1931.2385624 | 9.1623 | 0.000 | −1930.850311 |  |
| ***S*-35d.T2b** | 86.7787 | −1931.2385409 | 9.1758 | 0.000 | −1930.850316 |  |
| ***S*-35d.T2f** | 87.7646 | −1931.2383282 | 9.3093 | 0.000 | −1930.849918 |  |
| ***S*-35d.T2k** | 89.1601 | −1931.2373550 | 9.9200 | 0.000 | −1930.849970 |  |
| ***S*-35d.T2i** | 88.7638 | −1931.2373143 | 9.9455 | 0.000 | −1930.849443 |  |
| ***S*-35d.T2l** | 89.2361 | −1931.2372769 | 9.9690 | 0.000 | −1930.849487 |  |
| ***S*-35d.T2j** | 88.8696 | −1931.2372333 | 9.9964 | 0.000 | −1930.850336 |  |
| ***S*-35d.T2g** | 87.9052 | −1931.2371686 | 10.0370 | 0.000 | −1930.849870 |  |
| ***S*-35d.T2h** | 88.0282 | −1931.2370683 | 10.0999 | 0.000 | −1930.850612 |  |

**Supplementary Table S2.** Rotational strengths in dipole length formalism (*R_j_*), oscillator strengths (*f_j_*) and excitation wavelengths (*λ_j_*) for the first 50 electronic transitions of the conformers of *S*-35d, as obtained by TD-DFT calculations at the PBE0-⅓/def2-TZVPD//B97D3/def2-TZVP/fit level (IEFPCM solvation model for methanol).

| ***j*** | ***R_j_*, 10^−40^ erg cm^3^; *f_j_* (*λ_j_*, nm)** | | | | | |
| --- | --- | --- | --- | --- | --- | --- |
|  | ***S*-35d.T1a** | ***S*-35d.T1b** | ***S*-35d.T1c** | ***S*-35d.T1d** | ***S*-35d.T1e** | ***S*-35d.T1f** |
| **1** | −47.2263; 0.3798 (326.03) | −57.8956; 0.3962 (327.06) | −19.9966; 0.2934 (318.26) | −23.7371; 0.2984 (318.27) | −38.9541; 0.3656 (324.70) | −46.1781; 0.3798 (325.62) |
| **2** | 4.7802; 0.0141 (307.15) | 3.8999; 0.0172 (307.29) | −7.8043; 0.0176 (301.18) | −8.1573; 0.0213 (300.79) | 1.2441; 0.0229 (305.32) | 0.0901; 0.0283 (305.42) |
| **3** | 0.3218; 0.0117 (298.35) | 0.3578; 0.0066 (298.43) | 0.6645; 0.0222 (292.34) | −0.8094; 0.0190 (291.99) | 1.0151; 0.0155 (297.74) | 0.9567; 0.0086 (297.89) |
| **4** | 31.6301; 0.0516 (275.00) | 29.3092; 0.0475 (275.43) | 32.9481; 0.0676 (270.64) | 30.1287; 0.0672 (270.78) | 32.7611; 0.0516 (274.74) | 30.4207; 0.0493 (275.31) |
| **5** | −14.2837; 0.0310 (263.52) | −16.3001; 0.0304 (263.98) | −19.4706; 0.0435 (261.95) | −22.2568; 0.0473 (261.87) | −12.1572; 0.0281 (263.23) | −13.3222; 0.0280 (263.53) |
| **6** | −29.3126; 0.0497 (256.79) | −29.0079; 0.0509 (257.14) | −20.8603; 0.0243 (252.90) | −20.0871; 0.0255 (252.85) | −26.1728; 0.0431 (256.53) | −27.0932; 0.0461 (256.88) |
| **7** | −9.6220; 0.0053 (249.77) | −0.0125; 0.0045 (250.29) | 52.5601; 0.0195 (248.28) | −0.6708; 0.0020 (248.44) | −11.8031; 0.0061 (249.58) | −2.4052; 0.0042 (250.11) |
| **8** | 68.0580; 0.0248 (248.85) | 6.0564; 0.0210 (247.60) | −5.1547; 0.0024 (248.17) | 28.5853; 0.0202 (246.94) | 47.1795; 0.0196 (248.71) | 11.0553; 0.0197 (247.51) |
| **9** | −138.6357; 0.1661 (247.20) | −87.1809; 0.1718 (246.97) | −128.3086; 0.2356 (243.09) | −77.6746; 0.1653 (242.81) | −124.2858; 0.1744 (245.60) | −98.5687; 0.1754 (245.50) |
| **10** | −49.7909; 0.0383 (240.76) | −54.5438; 0.0446 (240.49) | −37.1435; 0.0236 (242.47) | −80.2423; 0.1143 (242.47) | −62.2169; 0.0381 (240.90) | −71.6632; 0.0475 (240.84) |
| **11** | 2.8353; 0.0099 (238.56) | −5.4138; 0.0281 (238.77) | 19.2741; 0.0513 (235.59) | 22.1165; 0.0859 (235.53) | 2.0976; 0.0071 (238.36) | −3.1011; 0.0228 (238.58) |
| **12** | −14.1089; 0.0451 (236.27) | −17.7872; 0.0694 (236.18) | 47.3899; 0.3058 (234.65) | 44.9866; 0.2753 (234.37) | −15.8109; 0.0440 (235.35) | −19.8344; 0.0641 (235.40) |
| **13** | 40.4965; 0.5334 (234.04) | 48.3123; 0.5179 (233.71) | −11.5995; 0.1682 (232.74) | −16.3805; 0.1666 (232.68) | 137.9702; 0.3701 (234.09) | 104.7661; 0.1455 (234.26) |
| **14** | −25.1519; 0.0041 (231.49) | −25.4998; 0.0099 (231.96) | −30.7630; 0.0207 (230.16) | −37.6542; 0.0169 (230.05) | −102.8644; 0.1982 (233.43) | −62.5052; 0.4229 (233.40) |
| **15** | 5.4958; 0.0318 (230.59) | 6.3942; 0.0515 (230.29) | −3.2367; 0.0028 (229.88) | −8.5796; 0.0065 (229.66) | 1.9855; 0.0320 (230.43) | 3.3052; 0.0393 (230.32) |
| **16** | −3.1215; 0.0012 (228.85) | −1.3622; 0.0074 (227.29) | −3.6529; 0.0039 (229.15) | 13.3003; 0.0095 (228.08) | −1.9564; 0.0009 (228.51) | −0.9014; 0.0051 (227.00) |
| **17** | 21.7281; 0.0358 (224.82) | 50.7766; 0.0689 (224.27) | 42.0363; 0.1164 (224.66) | −23.8095; 0.3176 (224.31) | 12.5452; 0.0642 (224.27) | 49.1905; 0.1199 (223.69) |
| **18** | −1.2343; 0.1887 (224.00) | −1.0123; 0.0943 (223.52) | −9.0955; 0.3215 (223.95) | 78.2340; 0.1547 (223.72) | 18.8926; 0.1994 (223.53) | 7.8985; 0.1219 (223.25) |
| **19** | 30.2492; 0.0219 (222.37) | 4.4777; 0.0098 (222.35) | 27.2622; 0.0338 (220.75) | 21.6237; 0.0202 (221.21) | 37.4521; 0.0392 (221.33) | 8.0599; 0.0095 (221.46) |
| **20** | 14.3777; 0.1452 (221.58) | −29.5776; 0.1920 (220.83) | −22.6118; 0.0298 (219.21) | −14.1790; 0.0335 (217.78) | −14.7095; 0.1171 (220.73) | −36.8014; 0.1583 (219.79) |
| **21** | 30.7379; 0.1056 (219.33) | 103.3300; 0.1117 (220.24) | −65.3557; 0.0259 (217.24) | −32.1140; 0.0215 (217.30) | 25.1250; 0.0781 (218.40) | 91.7950; 0.0836 (219.19) |
| **22** | 107.5888; 0.0585 (214.87) | 95.9569; 0.0578 (214.54) | 59.9826; 0.0566 (216.70) | 84.6320; 0.0672 (216.49) | 68.2727; 0.0358 (215.33) | 100.7494; 0.0321 (215.03) |
| **23** | −21.5432; 0.0570 (214.01) | 54.6986; 0.0394 (213.62) | −14.8788; 0.0506 (213.50) | 4.5666; 0.0564 (212.97) | −5.5964; 0.0562 (214.03) | −10.0619; 0.0559 (213.81) |
| **24** | 11.1079; 0.0061 (213.09) | −32.8756; 0.0191 (212.94) | 41.3448; 0.0507 (212.58) | −5.6168; 0.0296 (212.32) | 59.2220; 0.0325 (212.57) | 40.3459; 0.0307 (212.58) |
| **25** | −28.5363; 0.0120 (212.48) | −41.1510; 0.0226 (212.59) | 7.6344; 0.0195 (212.27) | 37.3032; 0.0338 (212.10) | −38.5799; 0.0219 (212.27) | −42.4190; 0.0211 (212.05) |
| **26** | 3.7836; 0.0033 (210.42) | −14.9164; 0.0056 (210.59) | 10.9448; 0.0290 (210.57) | −1.5860; 0.0309 (210.05) | −12.9443; 0.0029 (210.40) | −40.4190; 0.0133 (210.46) |
| **27** | −13.3719; 0.0118 (210.04) | 6.5318; 0.0060 (209.92) | 16.5874; 0.0215 (209.95) | 26.7349; 0.0487 (209.61) | −29.8089; 0.0111 (209.79) | 2.7288; 0.0108 (209.32) |
| **28** | −24.3342; 0.0061 (209.01) | 0.3839; 0.0074 (209.41) | 5.0867; 0.1730 (208.44) | −20.2698; 0.1066 (208.46) | 1.9221; 0.0018 (209.16) | 3.3580; 0.0082 (208.87) |
| **29** | 8.5160; 0.0051 (208.12) | −35.7128; 0.0206 (207.93) | 9.5442; 0.0070 (207.72) | 5.0817; 0.0145 (207.47) | 14.4315; 0.0054 (207.88) | −1.6955; 0.0118 (208.32) |
| **30** | 10.6785; 0.0263 (207.76) | 9.0110; 0.0076 (207.61) | 45.5371; 0.0194 (206.68) | −79.8127; 0.0506 (206.41) | 9.5261; 0.0576 (207.32) | −7.1982; 0.0104 (207.34) |
| **31** | −42.5285; 0.0490 (207.04) | −36.7415; 0.0435 (206.96) | −62.8454; 0.0523 (205.63) | 33.5377; 0.0325 (206.25) | −34.3023; 0.0239 (206.74) | −40.7647; 0.0454 (206.94) |
| **32** | −18.4488; 0.0859 (206.68) | −16.6200; 0.0580 (206.26) | −1.6685; 0.0006 (205.47) | −8.7635; 0.0118 (205.49) | −22.6092; 0.0692 (206.23) | 2.3742; 0.0163 (206.03) |
| **33** | 7.3467; 0.0139 (206.11) | 5.0009; 0.0595 (206.14) | 16.1099; 0.0155 (205.20) | −24.3164; 0.0316 (205.27) | −1.0777; 0.0032 (205.85) | −23.9535; 0.0785 (205.88) |
| **34** | −1.2666; 0.0361 (205.23) | −10.5500; 0.0620 (205.17) | −17.6675; 0.0177 (204.19) | −5.3261; 0.0534 (204.69) | −31.5648; 0.0482 (204.93) | −31.6791; 0.0854 (205.08) |
| **35** | −63.8786; 0.0752 (204.63) | −54.5490; 0.0436 (204.37) | −33.1125; 0.0728 (203.34) | 10.2815; 0.0364 (203.52) | −59.8553; 0.0987 (204.34) | −45.4737; 0.0560 (204.23) |
| **36** | −21.1207; 0.0389 (203.53) | −22.0945; 0.0444 (203.79) | −1.2012; 0.0023 (201.80) | 22.1910; 0.0261 (201.88) | −8.3906; 0.0160 (202.81) | −6.7964; 0.0132 (202.85) |
| **37** | 68.3443; 0.0553 (201.70) | 54.2819; 0.0387 (201.70) | 32.3379; 0.0320 (201.55) | −3.2950; 0.0070 (201.26) | 71.0619; 0.0644 (201.73) | 63.6253; 0.0576 (201.78) |
| **38** | −18.8335; 0.0243 (201.35) | −74.3938; 0.0383 (201.50) | −9.8930; 0.0043 (200.93) | −2.4626; 0.0062 (200.81) | −14.4214; 0.0334 (201.22) | −79.0720; 0.0325 (201.39) |
| **39** | −38.2996; 0.0105 (200.87) | 62.6501; 0.0242 (200.86) | 4.3743; 0.0607 (200.43) | 8.1682; 0.0520 (200.43) | −40.1566; 0.0077 (200.70) | 58.9914; 0.0216 (200.53) |
| **40** | 105.4065; 0.0498 (199.39) | 90.1237; 0.0438 (199.57) | 8.6847; 0.0900 (198.35) | −3.6792; 0.0996 (198.40) | 123.3154; 0.0556 (199.10) | 104.6230; 0.0481 (199.24) |
| **41** | −33.6391; 0.0204 (198.25) | 3.7036; 0.0044 (197.98) | −21.7049; 0.0079 (198.05) | −48.3398; 0.0302 (197.41) | −1.6812; 0.0156 (198.37) | −4.8110; 0.0145 (198.25) |
| **42** | 32.4388; 0.1336 (197.72) | −65.9091; 0.1551 (197.60) | −16.8111; 0.0141 (197.25) | 17.5410; 0.0055 (196.82) | −3.8114; 0.1168 (197.76) | −69.9488; 0.1571 (197.49) |
| **43** | −31.2929; 0.0147 (197.05) | −0.0489; 0.0020 (196.75) | −139.2893; 0.1996 (196.53) | −234.8506; 0.1553 (196.55) | −48.7077; 0.0417 (197.01) | −19.3218; 0.0035 (196.72) |
| **44** | −77.7853; 0.1635 (195.83) | −124.4420; 0.1276 (195.53) | −3.4522; 0.0339 (196.10) | 46.0984; 0.0336 (196.06) | −54.4509; 0.1609 (195.78) | −121.5502; 0.1162 (195.62) |
| **45** | 29.5787; 0.0758 (195.00) | 45.7955; 0.0370 (194.85) | 31.1694; 0.0960 (195.40) | 94.6249; 0.0858 (195.48) | −33.2263; 0.0062 (195.01) | 9.5707; 0.0078 (195.16) |
| **46** | −129.7173; 0.0469 (193.89) | −183.1979; 0.0721 (193.47) | 10.6809; 0.1221 (194.46) | 12.4850; 0.0496 (194.58) | 37.3827; 0.0718 (194.84) | 58.5794; 0.0423 (194.75) |
| **47** | −34.7337; 0.2035 (193.52) | −65.0360; 0.1185 (193.22) | 7.7263; 0.0296 (194.04) | −15.1875; 0.0239 (194.00) | −121.5420; 0.0356 (193.75) | −156.8316; 0.0529 (193.36) |
| **48** | −22.4784; 0.0047 (192.82) | −26.0870; 0.0369 (192.83) | 23.0738; 0.0471 (193.92) | 25.0379; 0.0969 (193.80) | −26.1945; 0.2082 (193.37) | −87.2754; 0.1578 (193.05) |
| **49** | −127.7997; 0.0443 (192.02) | 5.5463; 0.0580 (192.27) | 93.3764; 0.0242 (192.17) | 48.1579; 0.0128 (192.57) | −181.6738; 0.0435 (191.85) | −14.2317; 0.0536 (192.09) |
| **50** | 48.1072; 0.0393 (191.66) | −25.6406; 0.0453 (191.43) | 24.7043; 0.2091 (191.78) | 79.5249; 0.1259 (191.23) | −2.0205; 0.0070 (191.44) | −19.3874; 0.0141 (191.06) |

**Supplementary Table S2.** (*continued*)

| ***j*** | ***R_j_*, 10^−40^ erg cm^3^; *f_j_* (*λ_j_*, nm)** | | | | | |
| --- | --- | --- | --- | --- | --- | --- |
|  | ***S*-35d.T1g** | ***S*-35d.T1h** | ***S*-35d.T1i** | ***S*-35d.T1j** | ***S*-35d.T1k** | ***S*-35d.T1l** |
| **1** | 76.7406; 0.4859 (349.21) | 72.9614; 0.4913 (348.37) | 67.1864; 0.5061 (349.78) | 61.4105; 0.5088 (349.33) | 85.8633; 0.4554 (335.65) | 77.7304; 0.4582 (334.73) |
| **2** | −25.2698; 0.0287 (321.06) | −22.3522; 0.0276 (320.67) | −29.7689; 0.0271 (321.23) | −26.3457; 0.0273 (321.16) | −21.7548; 0.0358 (316.06) | −17.0991; 0.0356 (315.54) |
| **3** | −12.2336; 0.0047 (303.41) | −11.2935; 0.0063 (302.84) | −10.6665; 0.0035 (305.00) | −10.5935; 0.0047 (304.65) | −13.3183; 0.0040 (299.66) | −12.6901; 0.0057 (299.22) |
| **4** | −0.0473; 0.0423 (280.79) | −0.3685; 0.0414 (280.18) | −5.4712; 0.0497 (282.24) | −5.5255; 0.0501 (281.54) | 15.0370; 0.0397 (274.59) | 16.5824; 0.0385 (274.23) |
| **5** | 24.6035; 0.0614 (270.84) | 24.3991; 0.0634 (270.58) | 20.6636; 0.0445 (271.73) | 21.2784; 0.0473 (271.53) | 16.7152; 0.0758 (267.08) | 15.3046; 0.0770 (266.83) |
| **6** | 18.4242; 0.0823 (261.54) | 13.0435; 0.0832 (261.34) | 12.6579; 0.0698 (261.81) | 8.2266; 0.0724 (261.74) | 19.0732; 0.0623 (258.31) | 12.8657; 0.0622 (258.05) |
| **7** | 0.7875; 0.0020 (255.59) | 1.2376; 0.0020 (255.33) | 1.9154; 0.0016 (255.77) | 2.1377; 0.0017 (255.59) | −3.5278; 0.0143 (253.65) | −4.9115; 0.0145 (253.53) |
| **8** | 150.6015; 0.1717 (251.50) | 121.6296; 0.1796 (251.30) | 148.5125; 0.1567 (250.47) | 121.1388; 0.1671 (250.35) | 193.3098; 0.1439 (248.57) | 117.6560; 0.1709 (248.25) |
| **9** | 4.2617; 0.0213 (248.56) | 23.9710; 0.0170 (247.85) | 28.3846; 0.0172 (248.83) | 38.7324; 0.0189 (248.21) | −22.2669; 0.0404 (247.62) | 44.4303; 0.0225 (246.94) |
| **10** | −12.8233; 0.0219 (244.02) | −8.2081; 0.0197 (243.75) | −64.9007; 0.0268 (245.64) | −41.6384; 0.0119 (245.12) | −14.5422; 0.0098 (242.42) | −2.8653; 0.0064 (241.51) |
| **11** | −29.8685; 0.0275 (243.71) | −18.3722; 0.0240 (242.96) | 3.9717; 0.0180 (243.13) | 16.6571; 0.0131 (243.27) | 2.7226; 0.0203 (240.40) | 6.1573; 0.0084 (240.14) |
| **12** | 9.4490; 0.0038 (240.07) | 4.0006; 0.0024 (240.42) | 14.3536; 0.0303 (242.74) | −1.6502; 0.0380 (243.08) | 25.1205; 0.0352 (239.37) | 16.8943; 0.0416 (239.94) |
| **13** | −94.7227; 0.2158 (235.23) | −102.0799; 0.2616 (235.01) | −112.3048; 0.2401 (235.01) | −118.8647; 0.2777 (234.81) | −28.3139; 0.7312 (233.69) | −64.4726; 0.6675 (233.68) |
| **14** | 49.6812; 0.4580 (233.27) | 61.7866; 0.4375 (233.23) | 49.8687; 0.4441 (233.18) | 59.2013; 0.4252 (233.21) | −41.5975; 0.0203 (233.53) | 12.4458; 0.1048 (233.43) |
| **15** | −59.4270; 0.0355 (231.76) | −49.5686; 0.0266 (231.71) | −52.0896; 0.0399 (231.38) | −44.6062; 0.0332 (231.41) | −72.9436; 0.0727 (231.21) | −72.8701; 0.0767 (231.03) |
| **16** | 5.5053; 0.1178 (228.23) | 14.1865; 0.1122 (228.10) | 11.0040; 0.1260 (227.31) | 18.5094; 0.1248 (227.47) | 16.8322; 0.0649 (227.93) | 17.5839; 0.0573 (227.82) |
| **17** | −21.0611; 0.0098 (226.79) | −4.3917; 0.0010 (224.51) | −34.1789; 0.0159 (226.40) | −8.7362; 0.0033 (224.18) | −36.0019; 0.0211 (223.57) | −20.4874; 0.0029 (222.52) |
| **18** | 9.8661; 0.0921 (223.10) | 7.1450; 0.0858 (223.04) | 22.6288; 0.1148 (222.48) | 19.2543; 0.1093 (222.36) | −21.3928; 0.0071 (222.63) | −8.7212; 0.0343 (221.40) |
| **19** | −1.8540; 0.0394 (220.93) | −1.6151; 0.0642 (220.72) | 12.5731; 0.0172 (220.61) | 15.2427; 0.0038 (220.41) | 17.4084; 0.0795 (219.20) | −12.1799; 0.0839 (218.92) |
| **20** | 50.2362; 0.0382 (219.58) | 5.2605; 0.1260 (219.91) | −12.6999; 0.0989 (219.53) | −18.0859; 0.1858 (220.17) | −21.5577; 0.1797 (218.29) | 15.4517; 0.1616 (218.36) |
| **21** | −66.9840; 0.1448 (219.12) | −10.5143; 0.0237 (219.09) | −21.7208; 0.0778 (219.20) | −7.3825; 0.0062 (219.26) | 29.2418; 0.0616 (217.16) | 28.8998; 0.0562 (217.18) |
| **22** | −13.8953; 0.0531 (216.83) | −20.6810; 0.0872 (216.91) | −14.7143; 0.0195 (216.44) | −13.5612; 0.0291 (216.39) | 36.1195; 0.0577 (216.34) | 38.5490; 0.0826 (216.50) |
| **23** | −13.9388; 0.0174 (216.05) | −14.4477; 0.0207 (216.07) | −11.3111; 0.0082 (215.37) | −12.7240; 0.0209 (215.66) | −61.2837; 0.0290 (214.60) | −69.0280; 0.0290 (214.35) |
| **24** | 15.3862; 0.0652 (212.60) | −7.1161; 0.0854 (213.43) | 39.7826; 0.0747 (212.83) | 7.7903; 0.1117 (213.66) | −23.9928; 0.0027 (213.83) | −24.4317; 0.0039 (214.01) |
| **25** | 7.6246; 0.0053 (211.96) | 13.4971; 0.0170 (212.09) | −13.9245; 0.0040 (212.52) | −16.9400; 0.0017 (212.63) | 5.1886; 0.0143 (210.08) | −10.2021; 0.0317 (211.19) |
| **26** | 9.9400; 0.0168 (211.78) | 11.8716; 0.0125 (211.67) | 0.5245; 0.0076 (211.78) | 8.8584; 0.0077 (211.79) | −8.4034; 0.0081 (209.65) | −4.5781; 0.0062 (209.57) |
| **27** | −9.9060; 0.0091 (210.95) | −0.9246; 0.0056 (210.95) | −4.4794; 0.0214 (211.36) | −1.9805; 0.0197 (211.31) | 3.6325; 0.0123 (209.12) | −2.6980; 0.0180 (209.21) |
| **28** | 3.2047; 0.0017 (209.45) | −2.1537; 0.0017 (209.64) | 1.4670; 0.0006 (209.46) | 1.6566; 0.0021 (209.39) | 8.1505; 0.0183 (208.77) | 1.9625; 0.0207 (208.63) |
| **29** | −6.5974; 0.0126 (208.31) | −6.9483; 0.0138 (208.28) | −2.0739; 0.0077 (208.12) | −3.5865; 0.0083 (208.06) | −14.6941; 0.0251 (207.86) | −6.1633; 0.0211 (207.82) |
| **30** | −19.0317; 0.0119 (206.97) | −20.5879; 0.0131 (206.93) | −13.0639; 0.0064 (207.26) | −20.0264; 0.0061 (207.04) | 5.1278; 0.0061 (207.56) | −4.5198; 0.0012 (207.61) |
| **31** | −1.8484; 0.0023 (206.71) | −0.0892; 0.0018 (206.63) | −2.9732; 0.0022 (206.78) | −10.2830; 0.0036 (206.73) | −64.5406; 0.0502 (207.18) | −72.4408; 0.0543 (207.13) |
| **32** | −21.3939; 0.0059 (206.02) | −29.0917; 0.0069 (205.77) | −14.6764; 0.0058 (206.68) | −7.3849; 0.0050 (206.67) | −0.7849; 0.0030 (205.91) | 0.2958; 0.0031 (205.88) |
| **33** | 43.8412; 0.0598 (205.34) | 40.0305; 0.0544 (205.29) | 42.4090; 0.0751 (205.30) | 41.5490; 0.0708 (205.26) | 28.2147; 0.0092 (205.07) | 22.9046; 0.0119 (205.08) |
| **34** | −24.0149; 0.0085 (204.54) | −27.0196; 0.0107 (204.54) | −5.4368; 0.0178 (204.15) | −6.5368; 0.0183 (204.15) | −4.6076; 0.0264 (204.21) | −4.3239; 0.0104 (204.27) |
| **35** | −112.9945; 0.0892 (203.31) | −99.2683; 0.0823 (203.22) | −201.1076; 0.1399 (203.06) | −187.5796; 0.1245 (202.99) | −6.3673; 0.0669 (203.79) | 7.6593; 0.0788 (203.85) |
| **36** | −87.4922; 0.0997 (202.36) | −89.6515; 0.1083 (202.37) | −15.6885; 0.0479 (202.13) | −40.3117; 0.0596 (202.17) | 10.8126; 0.0148 (202.26) | 15.6862; 0.0138 (202.19) |
| **37** | 33.2645; 0.0717 (201.98) | 28.7427; 0.0640 (202.11) | −1.6914; 0.0302 (201.24) | 16.9064; 0.0495 (201.40) | −206.3156; 0.1378 (201.69) | −207.6055; 0.1306 (201.67) |
| **38** | −19.1461; 0.0327 (200.65) | −25.5511; 0.0419 (200.43) | −13.0318; 0.0545 (200.77) | −8.7952; 0.0429 (200.53) | −4.8569; 0.0072 (199.96) | 8.6783; 0.0132 (199.32) |
| **39** | 58.1922; 0.1184 (200.17) | 63.4536; 0.1091 (200.15) | 105.0678; 0.1880 (200.22) | 98.8337; 0.1659 (200.12) | 34.1289; 0.0839 (198.96) | 20.2071; 0.0981 (198.61) |
| **40** | −7.5025; 0.0087 (198.37) | −1.3197; 0.0026 (197.32) | −6.6078; 0.0066 (198.84) | −16.0075; 0.0148 (198.44) | 5.4565; 0.0330 (198.46) | 3.1332; 0.0029 (198.08) |
| **41** | −16.8911; 0.1449 (197.28) | −18.0315; 0.1393 (197.23) | −21.3391; 0.0131 (197.83) | −24.7423; 0.0412 (197.13) | 2.5101; 0.1978 (197.45) | 13.4481; 0.1840 (197.47) |
| **42** | −46.1761; 0.0927 (196.30) | −54.6366; 0.0951 (196.36) | −0.3175; 0.1051 (197.00) | 8.8388; 0.0639 (196.87) | −12.4547; 0.0336 (197.15) | −29.8380; 0.0200 (197.34) |
| **43** | 18.8332; 0.0207 (195.37) | 15.4569; 0.0053 (195.57) | −96.6236; 0.0979 (195.47) | −128.5748; 0.1051 (195.61) | −19.6246; 0.0694 (196.07) | −21.6530; 0.0979 (196.12) |
| **44** | −29.2537; 0.0181 (194.91) | −21.9072; 0.0251 (194.77) | 6.6103; 0.0140 (194.89) | 33.3734; 0.0051 (195.15) | 0.5318; 0.0479 (195.40) | 9.1743; 0.0445 (195.23) |
| **45** | −10.9230; 0.0052 (193.65) | −17.4832; 0.0027 (193.51) | 9.5435; 0.0112 (194.17) | 13.0671; 0.0072 (194.00) | 12.2582; 0.0701 (193.81) | 23.2756; 0.0626 (193.91) |
| **46** | 28.7103; 0.0359 (193.11) | 18.3201; 0.0302 (193.05) | −0.3747; 0.0016 (193.06) | 15.2299; 0.0161 (192.92) | 6.8385; 0.0544 (193.15) | −24.1957; 0.0565 (193.14) |
| **47** | −9.7268; 0.0031 (192.99) | −0.0230; 0.0018 (192.83) | 5.1171; 0.0361 (192.97) | −19.9786; 0.0172 (192.83) | −0.6966; 0.0058 (192.65) | −1.0404; 0.0016 (192.65) |
| **48** | 7.0545; 0.0075 (192.80) | 6.4470; 0.0106 (192.74) | 5.1203; 0.0013 (192.84) | 4.0852; 0.0021 (192.69) | 11.8646; 0.0072 (191.20) | 6.7050; 0.0070 (191.08) |
| **49** | 54.3189; 0.1602 (192.02) | 75.5148; 0.1891 (191.95) | −24.5603; 0.2869 (192.25) | 1.8166; 0.0765 (192.26) | −29.1618; 0.0890 (191.01) | −84.5481; 0.1387 (190.88) |
| **50** | −3.7947; 0.2070 (191.94) | 2.0512; 0.1510 (191.86) | 8.4257; 0.0566 (192.01) | −17.8498; 0.2463 (192.06) | 42.9853; 0.2474 (190.80) | 106.1184; 0.1662 (190.65) |


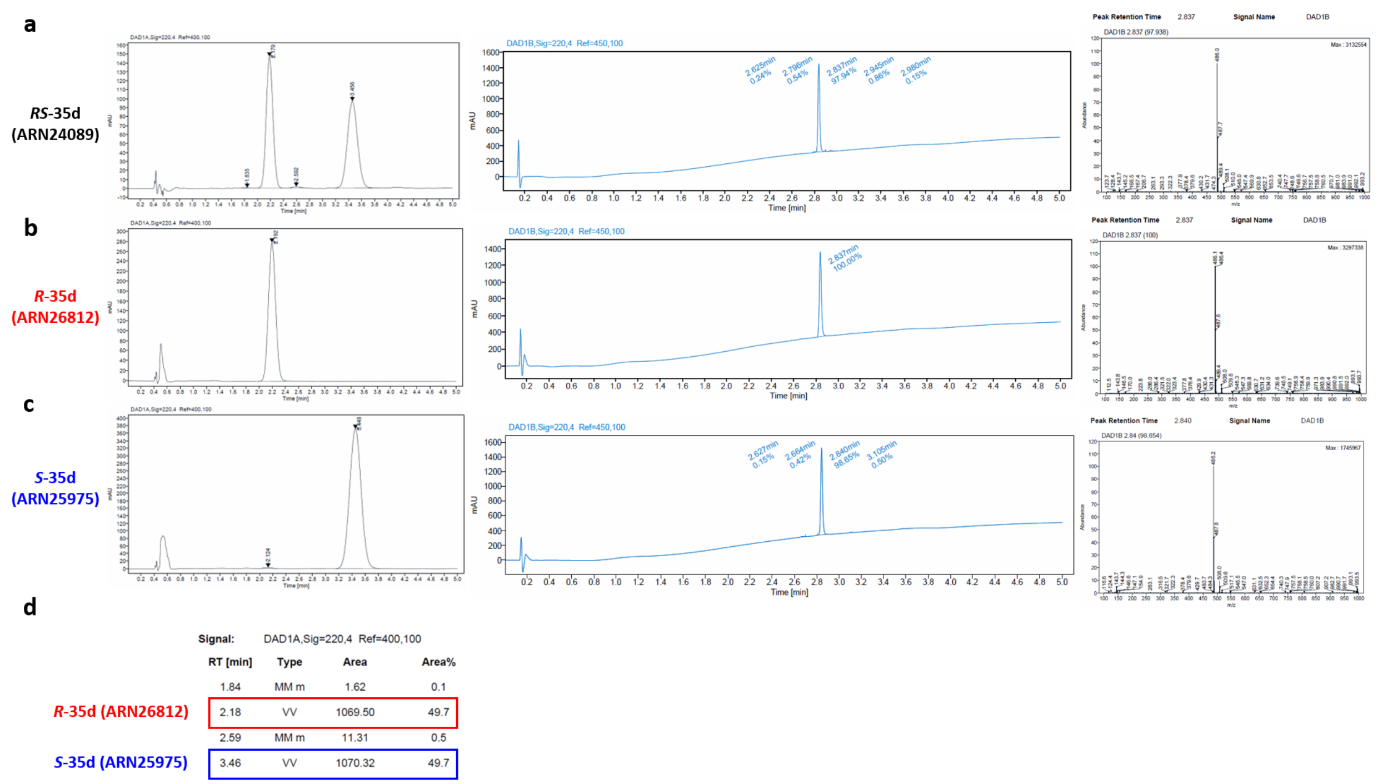


**Supplementary Fig. S1 | *RS*-35d enantiomers separation through supercritical fluid chromatography.** Chiral purity, chemical purity, and MS traces for *RS*-35d (ARN24089) (**a**), *R*-35d (ARN26812) (**b**) and *S*-35d (ARN25975) (**c**). (**d**) Retention time and area % for *R*-35d and *S*-35d.


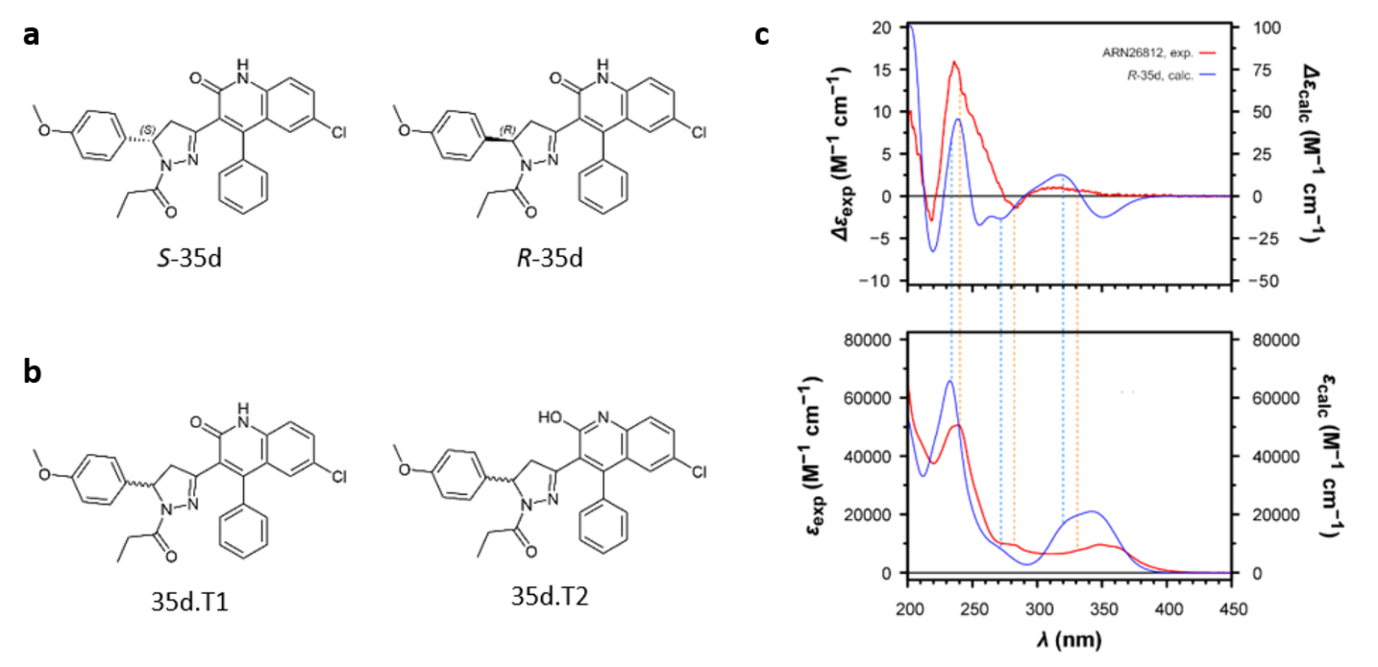


**Supplementary Fig. S2 | Stereochemical characterisation of *RS*-35d.** (**a**) Enantiomers of *RS*-35d. (**b**) Tautomeric structures of *RS*-35d considered for the conformational search by MM and DFT calculations. (**c**) Theoretical CD and UV spectra of *R*-35d, as calculated by TD-DFT calculations at the PBE0-⅓/def2-TZVPD//B97D3/def2-TZVP/fit level (IEFPCM solvation model for methanol), compared to the experimental CD and UV spectra of the first-eluted enantiomeric fraction of *R*-35d (ARN26812).

To gain structural insight into their interactions with their target, molecular docking calculations were performed with both *S*-35d and *R*-35d on the RAD51 structure with an inhibitor bound (PDB: 7EJC) within zone II, allowing to conduct our investigations on a more comprehensive structure compared to the outdated structure (PDB: 1N0W) previously utilised for virtual screening. *S*-35d binding mode predominantly relies on interactions carried out by the quinolone ring. Specifically, the NH group forms a hydrogen bond with Ala44, the carbonyl group forms a hydrogen bond with Tyr205, the ring engages in a π-cation interaction with Arg254, and Tyr205 participates in a π-π interaction with the quinolone ring. Conversely, for *R*-35d, the contacts involving the quinoline moiety persist, while the interaction between the carbonyl group linked to the quinolone ring and Tyr205 is absent. As for the rest of the molecule, a hydrogen bond occurs between the carbonyl linked to pyrazoline and Asn62, which does not occur for *S-*35d.


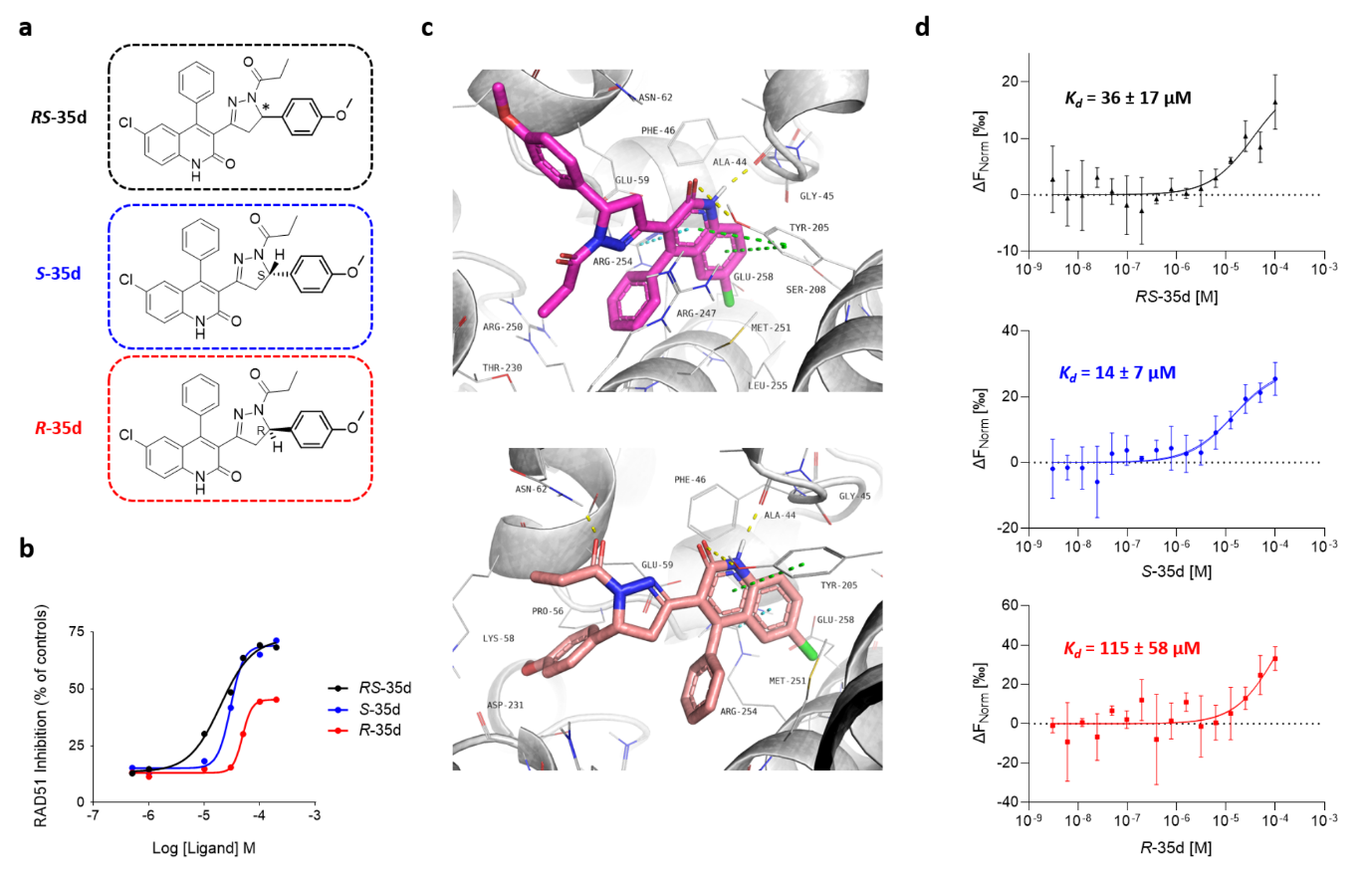


**Supplementary Fig. S3 | Enantiomers differential inhibiting activity towards RAD51-BRCA2.** (**a**) Structures of *RS*-35d racemate, *S*-35d and *R*-35d. (**b**) ELISA- based biological screening of *RS*-35d, *S*-35d and *R*-35d for their RAD51-BRC4 inhibiting activity (EC_50_ values of 20 ± 1, 30 ± 1 and 49 ± 1 µM for *RS*-35d, *S*-35d and *R*-35d respectively). Shown results are indicative of a representative independent replicate of the experiment, which consists of three replicates. (**c**) Binding mode proposed after Induced Fit Docking for *S*-35d (magenta stick) and *R*-35d (pink stick) on RAD51 (yellow dotted lines = hydrogen bonds; green dotted lines = π-π interactions; cyan dotted lines = π-ion interactions). (**d**) MST analysis of His-hRAD51 binding to *RS*-35d, *S*-35d and *R*-35d; titration curve of (RED-tris-NTA 2nd Generation)-HishRAD51 (50 nM) with increasing concentrations of RAD51-BRCA2 inhibitor. MST data are the average of three replicates. Sigmoidal curves were obtained using GraphPad PRISM.


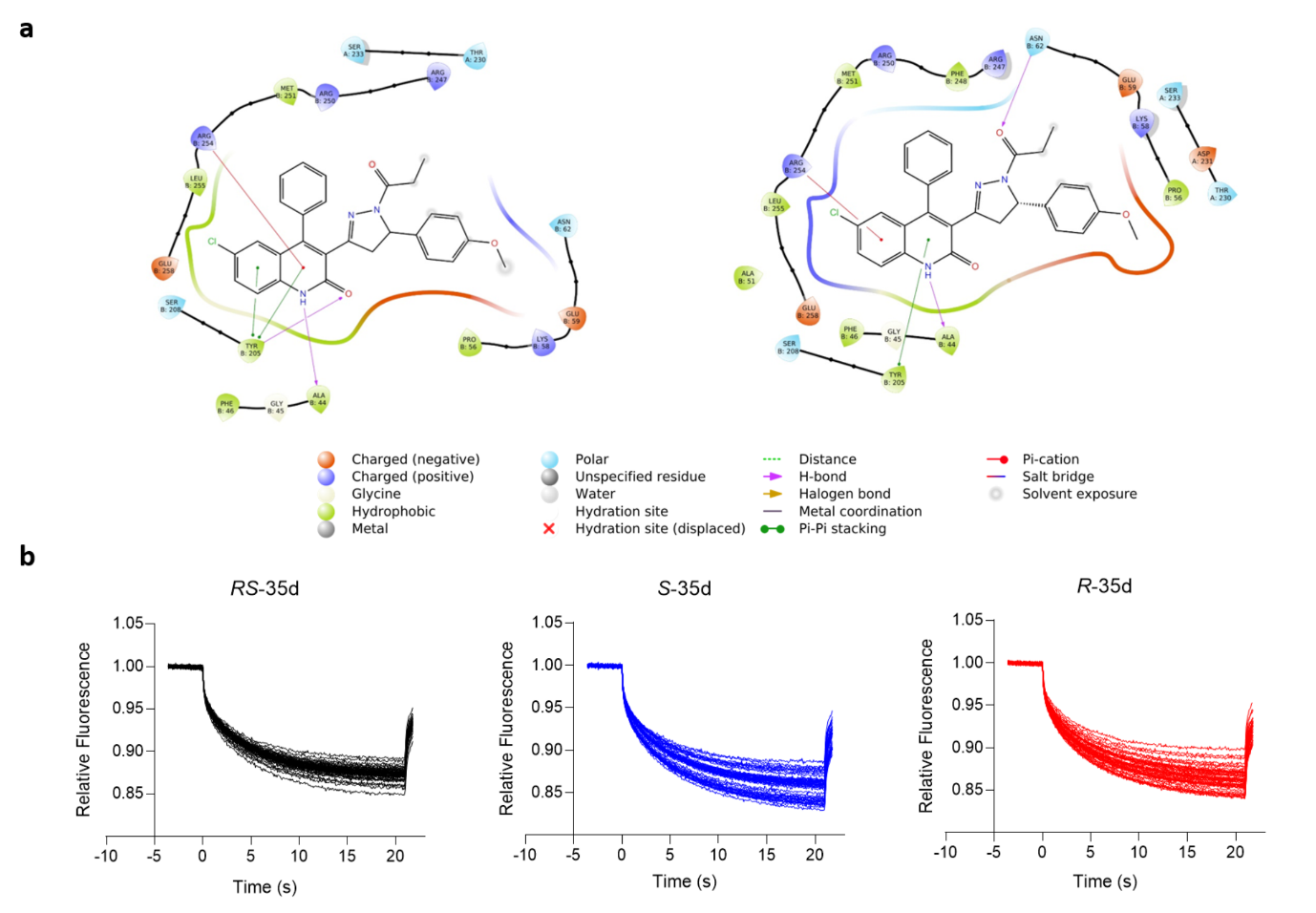


**Supplementary Fig. S4 | Molecular docking for *S*-35d and *R*-35d in RAD51 structure and MST traces of *RS*-35d, *S*-35d and *R*-35d binding to His-hRAD51.** (**a**) Ligand interaction diagram for *S*-35d and *R*-35d enantiomers with RAD51 structure. (**b**) MST traces of *RS*-35d, *S*-35d and *R*-35d binding to RED-tris-NTA-labelled His-hRAD51. MST measurement was performed using 40% MST power and 10% excitation power. An MST-on time of 20s was used for the analysis. Ligand-dependent changes in MST are plotted as F_norm_ (relative fluorescence normalised at 1) values vs ligand concentration in a dose-response curve. For each trace, the F_norm_ value for the dose-response curve is calculated as *F_norm_ = F_1_/F_0_* (F_1_ = relative fluorescence value measured in the heated state; F_0_ = relative fluorescence value measured in the cold state). F_norm_ values are plotted as parts per thousand (‰).


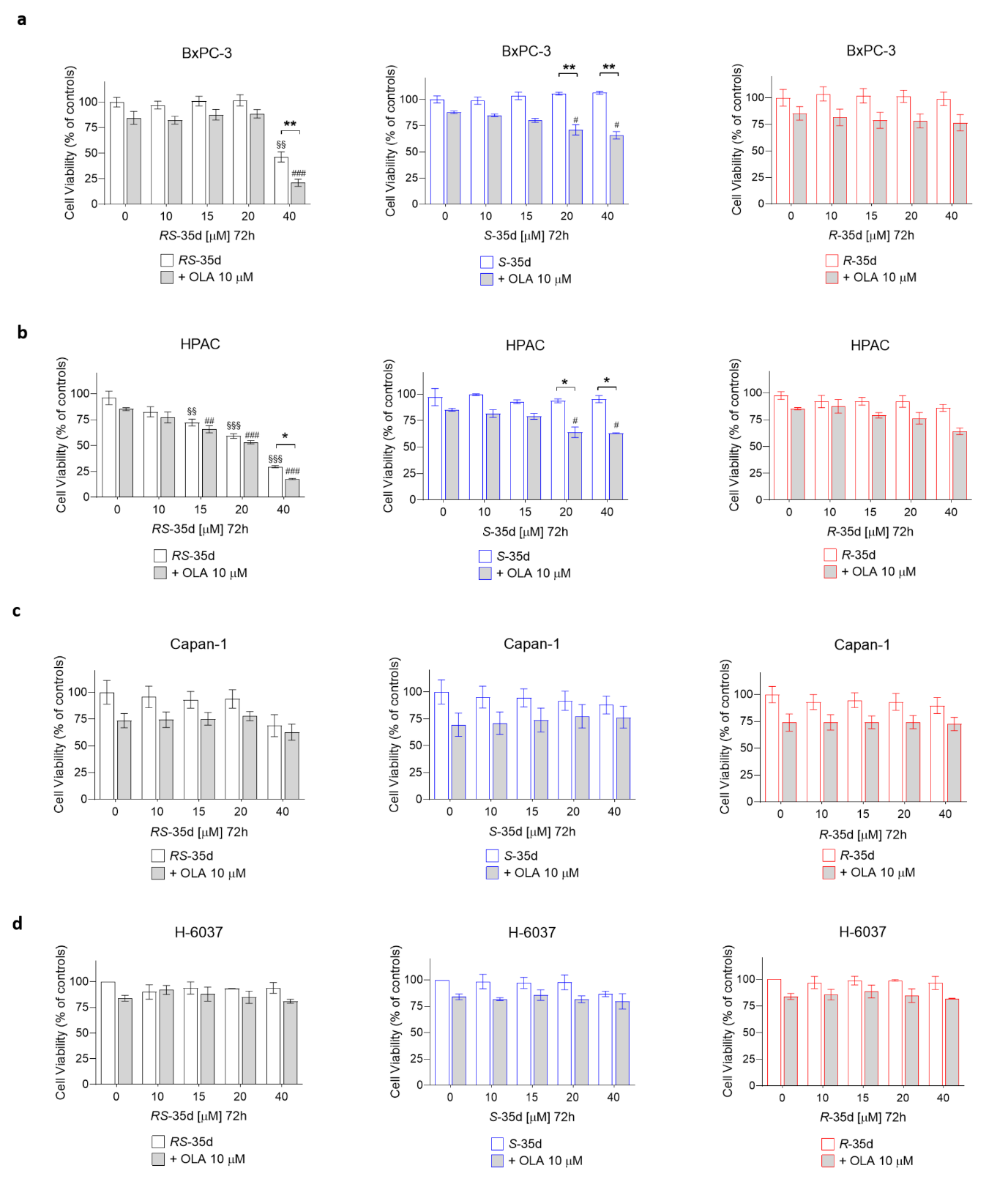


**Supplementary Fig. S5 | Complete cell viability graphs reporting all tested concentrations of *RS*-35d, *S*-35d and *R*-35d in BxPC-3, HPAC, Capan-1 and H-6037 cell lines.** **a-c** Cell viability measured after 72 h exposure to 0, 10, 15, 20, 40 µM *RS*-35d, *S*-35d or *R*-35d alone or in combination with 10 µM olaparib (OLA) in BxPC-3 (**a**), HPAC (**b**), Capan-1 (**c**) and H-6037 (**d**) cells. Statistical analysis was performed with two-way ANOVA followed by Tuckey’s multiple comparison test, with ^§§^*p* < 0.01 or ^§§§^*p* < 0.01 vs CTRL; ^#^*p* < 0.05 or ^###^*p* < 0.001 vs 10 µM OLA; ***p* < 0.01 vs RAD51-BRCA inhibitor alone.


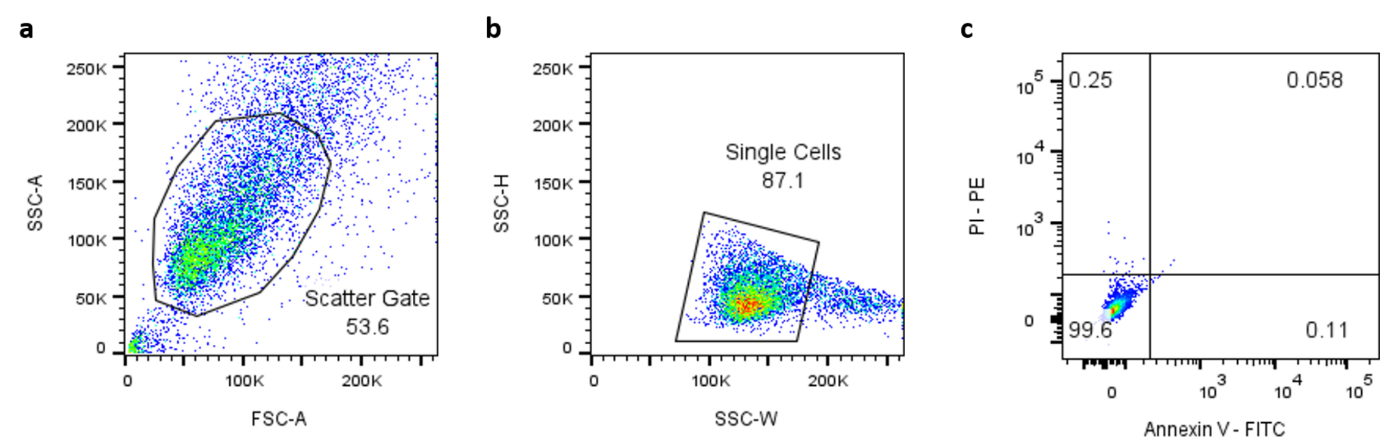


**Supplementary Fig. S6 | Gating strategy used in flow cytometry for apoptosis analysis.** (**a**) BxPC-3 cells were gated based on size and granularity using FSC-A vs SSC-A to eliminate debris and cell clusters. (**b**) BxPC-3 cells were further gated based on side scatter width (SSC-W) and side scatter height (SSC-H) to isolate single cells (singlets) from doublets or clumps. (**c**) Negative control (untreated cells) used to determine BxPC-3 cells levels of autofluorescence.


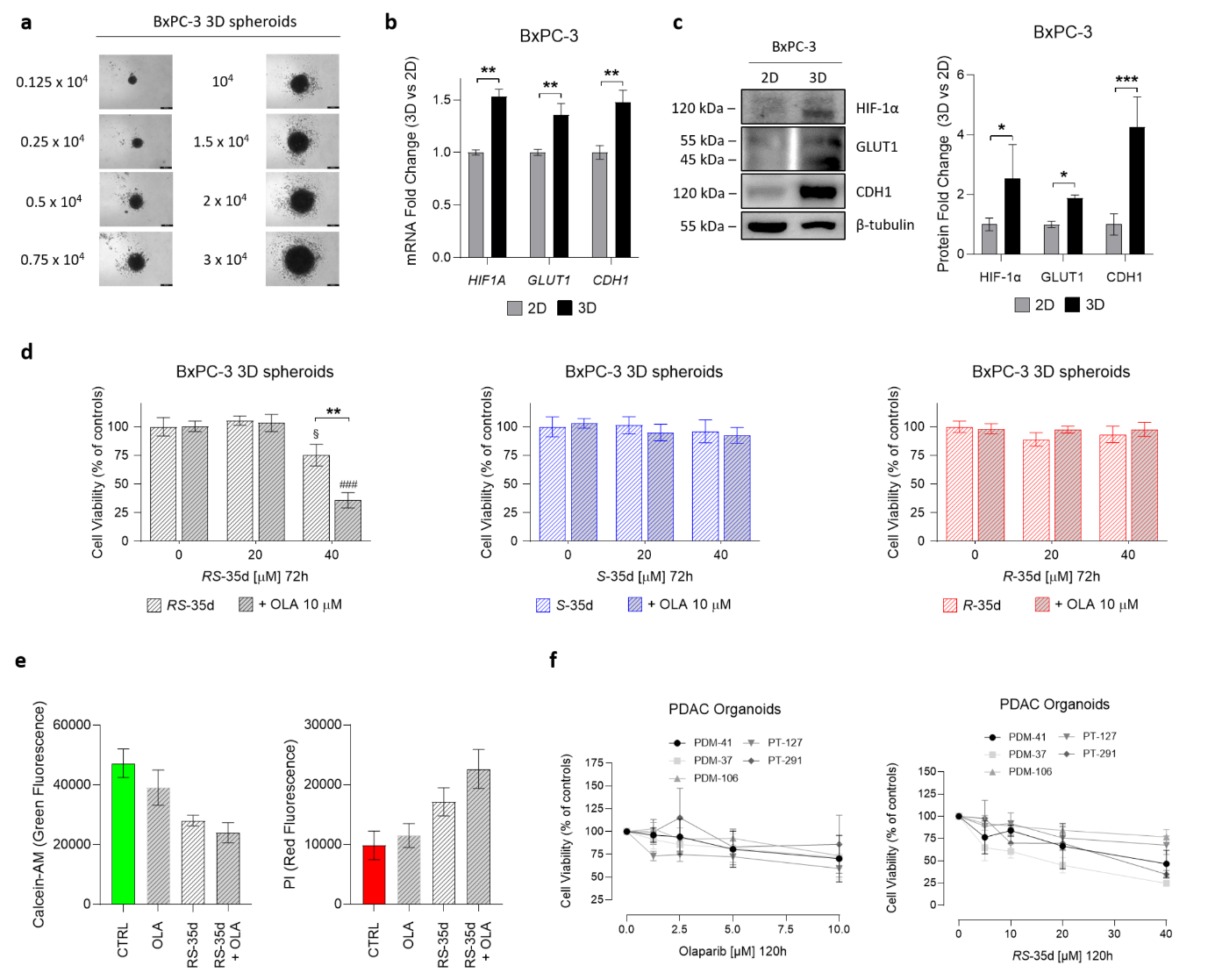


**Supplementary Fig. S7 | Characterisation of tumour-mimicking BxPC-3 3D spheroids and optimisation of single-agent treatments in human PDAC organoids.** (**a**) Representative images of 3D spheroids obtained at different BxPC-3 cell concentrations (scale bar, 200 μm). The 3·10^4^ cells/well concentration was used for all the following experiments. **b**,**c** BxPC-3 3D spheroids (3D) characterisation via Real-Time PCR (**b**) and Western blot (**c**) to assess their correct PDAC tumour-mimicking properties compared to 2D cultures (2D). (**b**) *HIF1A*, *GLUT1* and *CDH1* mRNA expression in 3D vs 2D BxPC-3 cells. *GAPDH* was used as endogenous reference control. (**c**) Representative Western blot images of HIF-1α, GLUT1 and CDH1 in 3D vs 2D BxPC-3 cells; densitometric analysis of the corresponding protein bands. Results are normalised over β-tubulin expression and expressed as mean ± SD of three independent replicates. Statistical analysis was performed with one-way ANOVA followed by Tuckey’s multiple comparison test, with **p* < 0.05, ***p* < 0.01 or ****p* < 0.001 vs the respective 2D value. (**d**) Complete cell viability analysis after 72 h treatment in BxPC-3 3D spheroids with *RS*-35d, *S*-35d or *R*-35d alone or in combination with 10 µM olaparib (OLA). Results are expressed as mean ± SD of at least three independent replicates. Statistical analysis was performed with two-way ANOVA followed by Tuckey’s multiple comparison test, with §p < 0.05 vs CTRL, ###p < 0.001 vs 10 µM OLA and **p < 0.01 vs RAD51-BRCA2 inhibitor alone. (**e**) Analysis of Calcein-AM (green fluorescence) and PI (red fluorescence) signals in *RS*-35d-treated BxPC-3 3D spheroids at 72 h. Measured values were used to calculate PI/Calcein-AM ratio to determine cell death rate. (**f**) Dose-response evaluation of single agents olaparib and *RS*-35d in PDM-37, PDM-41, PDM-106, PT-127 and PT-291 organoids.


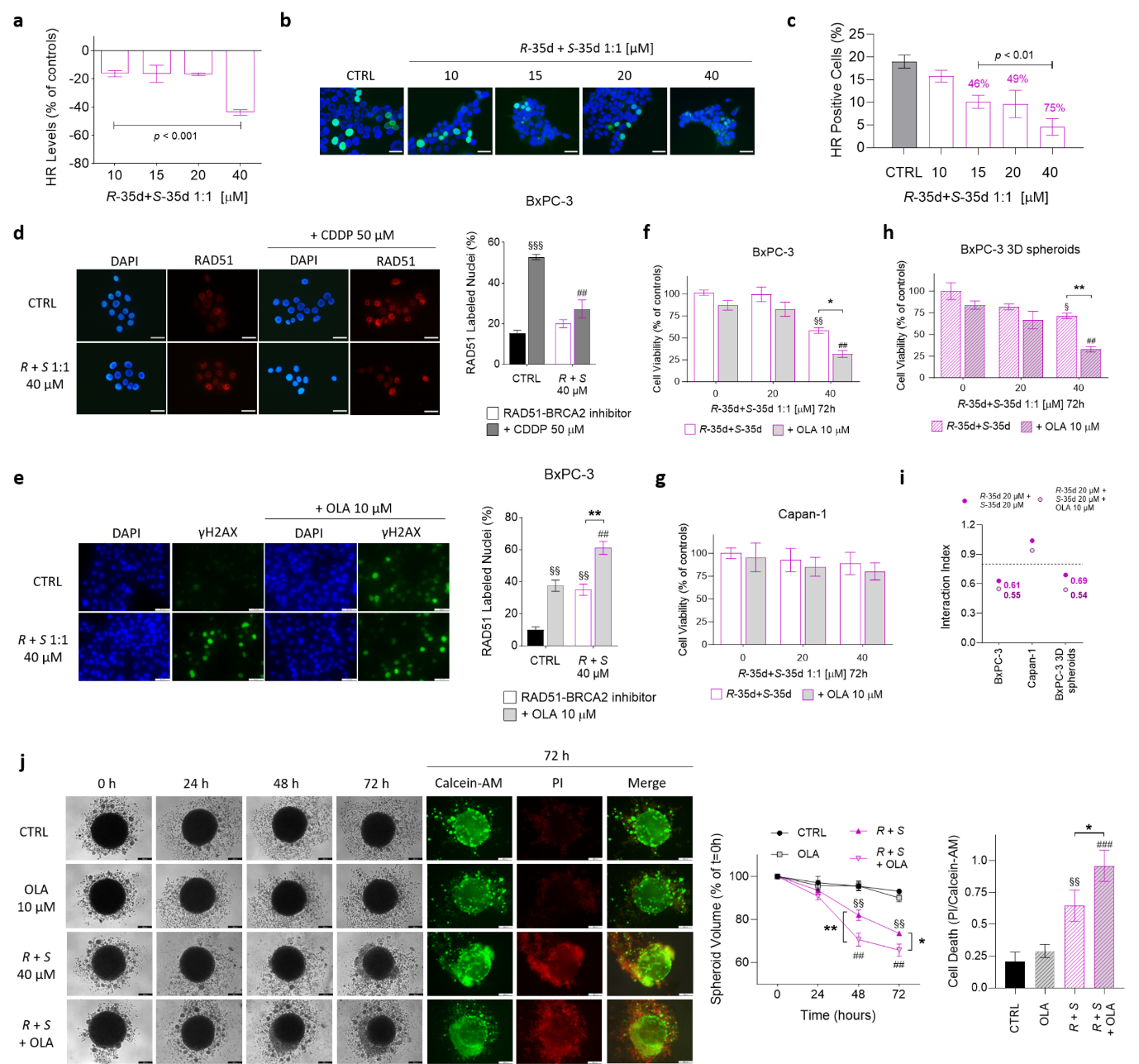


**Supplementary Fig. S8 | Characterisation of *S*-35d+*R*-35d reconstituted racemic mixture.** (**a**) Effect on HR caused by reconstituted racemic mixture (*R*+*S*) administration in BxPC-3 cells. (**b**) Representative merged images of mClover-Lamin A fusion protein and DAPI staining fluorescence after exposure to increasing doses of *R*+*S* (scale bar, 30 μm). (**c**) Analysis of HR-positive cells after administration of increasing doses of *R*+*S*. Results are expressed as mean ± SD of three independent experiments. Statistical analysis was performed with one-way ANOVA followed by Dunnett’s multiple comparison test, with *p* < 0.01 or *p* < 0.001 vs CTRL. (**d**) Immunofluorescence detection of nuclear RAD51 in BxPC-3 exposed to 40 µM *R*+*S* alone or after pre-treatment with 50 µM CDDP. Representative images of nuclear localisation of RAD51 immune-labelling (scale bar, 30 μm); analysis of RAD51-positive nuclei (%). Results are expressed as mean ± SD of three independent experiments. Statistical analysis was performed with one-way ANOVA followed by Tuckey’s multiple comparison test, with ^§§§^*p* < 0.001 vs CTRL and ^##^*p* < 0.01 vs 50 µM CDDP. (**e**) γH2AX foci immune detection in BxPC-3 cells treated for 48 h with 40 µM *R*+*S* alone or in combination with 10 µM OLA. Representative images of γH2AX immune-labelling (scale bar, 50 μm); analysis of γH2AX-positive nuclei (%). **f-h** Cell viability measured after 72 h exposure to 20 or 40 µM *R*+*S* alone or in combination with 10 µM OLA in BxPC-3 (**f**), Capan-1 (**g**) and BxPC-3 3D spheroids (**h**). (**i**) Interaction indices of *S*-35d and *R*-35d association and *R*+*S* combination with olaparib in BxPC-3, Capan-1 and BxPC-3 3D spheroids. (**j**) Evaluation of the effect of the 72 h treatment with 40 µM *R* + *S* alone or in combination with 10 µM olaparib (OLA) on BxPC-3 3D spheroid volume and cell death. Time-course representative brightfield and fluorescence images of BxPC-3 3D spheroids treated as previously described (scale bar, 200 μm); time-course analysis of the corresponding 3D spheroid volume (% of the spheroid volume at t = 0 h); analysis of the corresponding cell death in 3D spheroid at 72 h (PI/Calcein-AM ratio). Results are expressed as mean ± SD of three independent experiments. Statistical analysis was performed with two-way ANOVA (**f**,**g**,**h**,**j** *middle*) or one-way ANOVA (**e**,**j** *right*) followed by Tuckey’s multiple comparison test, ^§^*p* < 0.05 or ^§§^*p* < 0.01 vs CTRL; ^##^*p* < 0.01 or ^###^*p* < 0.001 vs 10 µM OLA; **p* < 0.05 or ***p* < 0.01 vs RAD51-BRCA disruptor alone.

Molecular docking calculations were performed on ATM, ATR and DNA-PK for *S*-35d and *R*-35d interactions. Available PDB structures are sufficient and of good quality, in terms of resolution and metrics, for ATM. However, there is no choice of structure for ATR and DNA-PK, and the available ones are of low quality. Additionally, in the case of ATM, the presence of inhibitors (PDBs: 7NI5 and 7NI4) helps in centring the grid and docking. For ATM, *S*-35d binding mode obtained for all studied structures is always the same, even after Induced Fit Docking. Specifically, a hydrogen bond from the carbonyl of the dihydroquinolone moiety to Lys2717, a hydrogen bond from the carbonyl linked to the pyrazolic ring to Val2696, a halogen bond from chlorine to Cys2770, and a π-π interaction of the phenyl substituent of the dihydroquinolone moiety with Trp2769. In some structures, an additional hydrogen bond of the pyrazolinic ring with Lys2717 is also possible. Conversely, for *R*-35d the binding mode is less confident. In some structures, it reflects that of *S*-35 with a rotation of the pyrazolinic ring, but sometimes it is different, even after Induced Fit Docking. This suggests that *R*-35d binding is more influenced by the environment and therefore weaker. Distinct behaviour was observed for *RS*-35d enantiomers in ATR and DNA-PK. Traditional docking yielded markedly different binding modes from those observed in ATM. With Induced Fit Docking, among the numerous binding possibilities, some poses resembled those seen in ATM. However, this outcome was anticipated considering the low quality of structures used for docking.


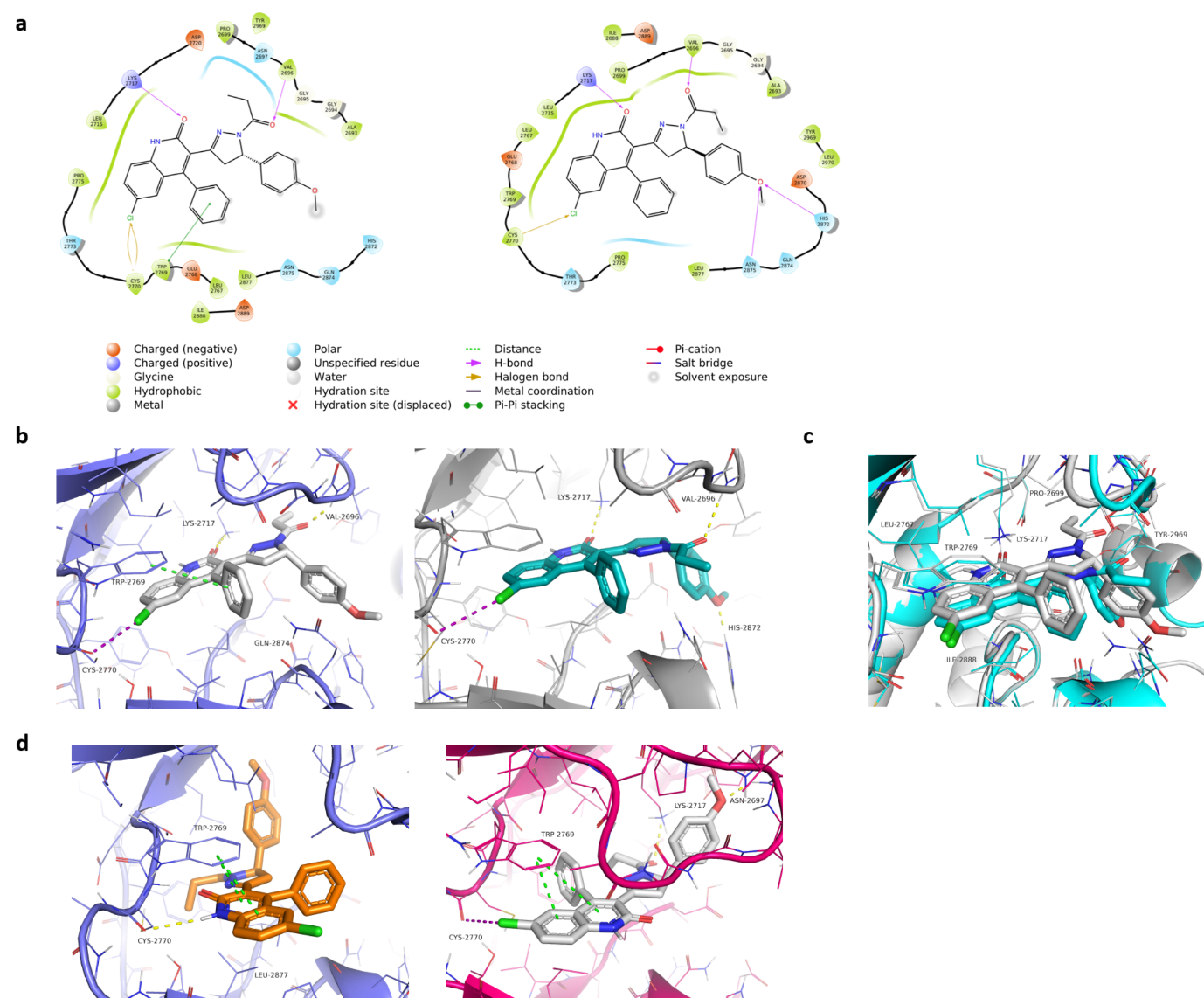


**Supplementary Fig. S9 | Molecular docking of *RS*-35d enantiomers in ATM structure.** (**a**) Ligand interaction diagram for *S*-35d and *R*-35d enantiomers in ATM structure. (**b**) Binding mode obtained by Induced Fit Docking on ATM for *S*-35d (white stick) and *R*-35d (cyan stick) (yellow = hydrogen bonds; magenta = halogen bonds; green = interactions π-π). (**c**) Binding modes for *S*-35d (white) and *R*-35d (cyan) superimposed in ATM structure. (**d**) Alternative binding modes of *R*-35d in ATM structure.


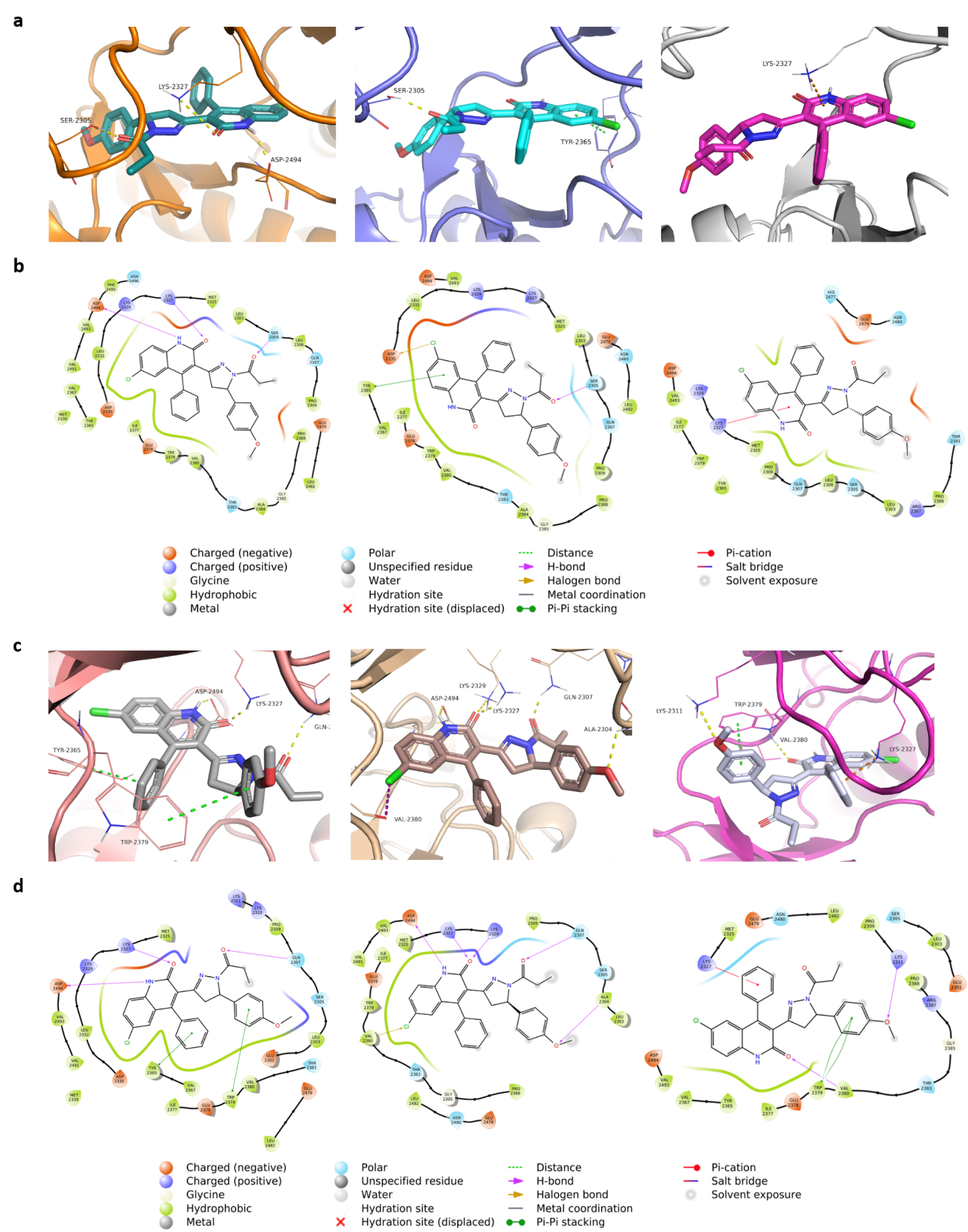


**Supplementary Fig. S10 | Molecular docking of *RS*-35d enantiomers in ATR structure.** **a-b** Three of the most probable alternative binding modes (**a**) and their respective ligand interaction diagram (**b**) of *S*-35d by Induced Fit Docking on ATR structure. **c-d** Three of the most probable alternative binding modes (**c**) and their respective ligand interaction diagram (**d**) of *R*-35d by Induced Fit Docking on ATR structure. Hydrogen bonds are represented in yellow, halogen bonds in magenta, π-cation orange and interactions π-π in green.


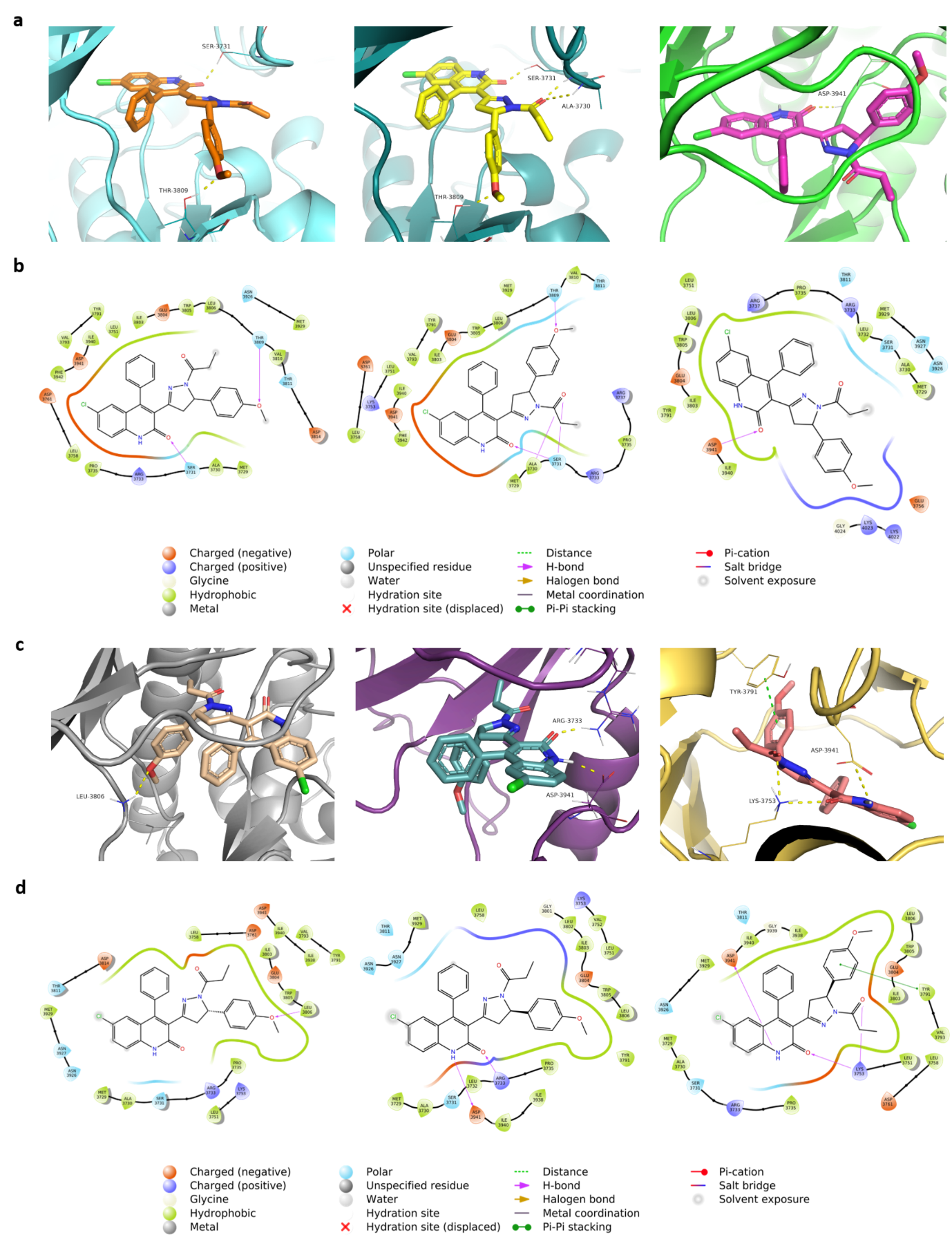


**Supplementary Fig. S11 | Molecular docking of *RS*-35d enantiomers in DNA-PK structure.** **a-b** Three of the most probable alternative binding modes (**a**) and their respective ligand interaction diagram (**b**) of *S*-35d by Induced Fit Docking on DNA-PK structure. **c-d** Three of the most probable alternative binding modes (**c**) and their respective ligand interaction diagram (**d**) of *R*-35d by Induced Fit Docking on DNA-PK structure. Hydrogen bonds are represented in yellow, halogen bonds in magenta, π-cation orange and interactions π-π in green.


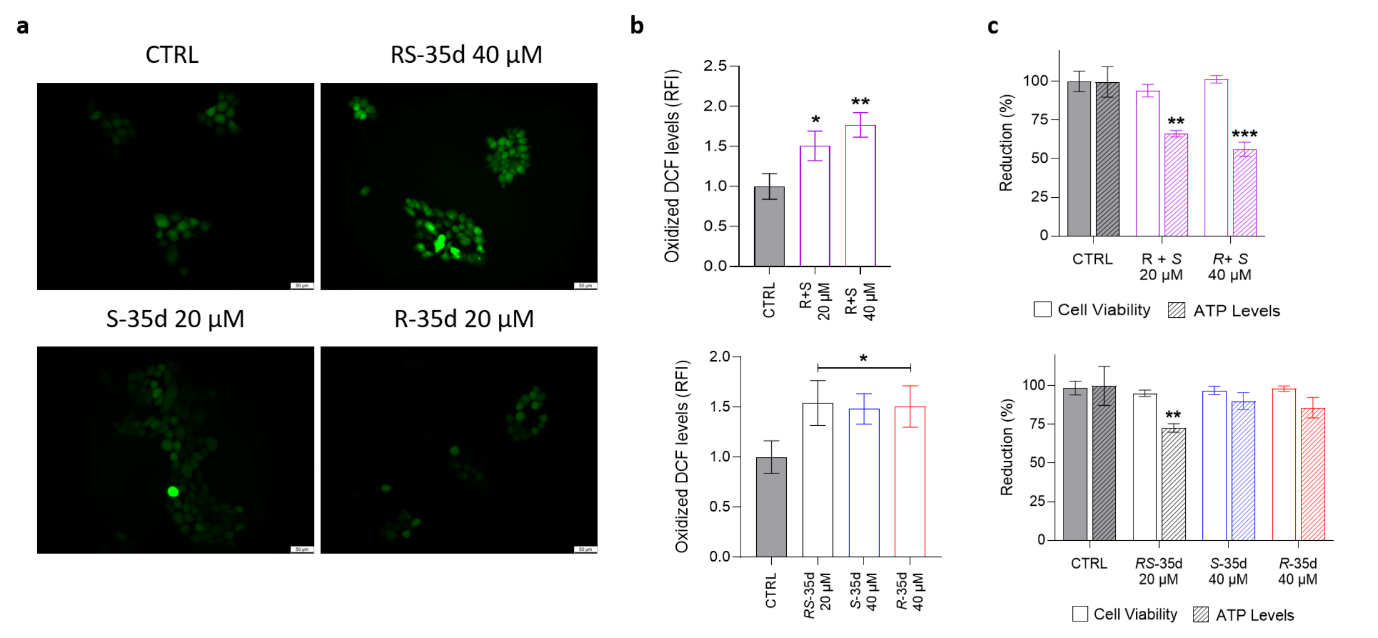


**Supplementary Fig. S12 | Further dissection of *RS*-35d mechanism of action.** (**a**) Fluorescence microscopic images of the effect of 24 h 40 µM *RS*-35d, 20 µM *S*-35d or 20 µM *R*-35d treatment on ROS production (DCF signal); **b-c** Effect of 20 µM *RS*-35d, 40 µM *S*-35d, 40 µM *R*-35d or 20/40 µM *R+S* treatment for 24 h on ROS production (**b**) and on ATP levels (**c**).


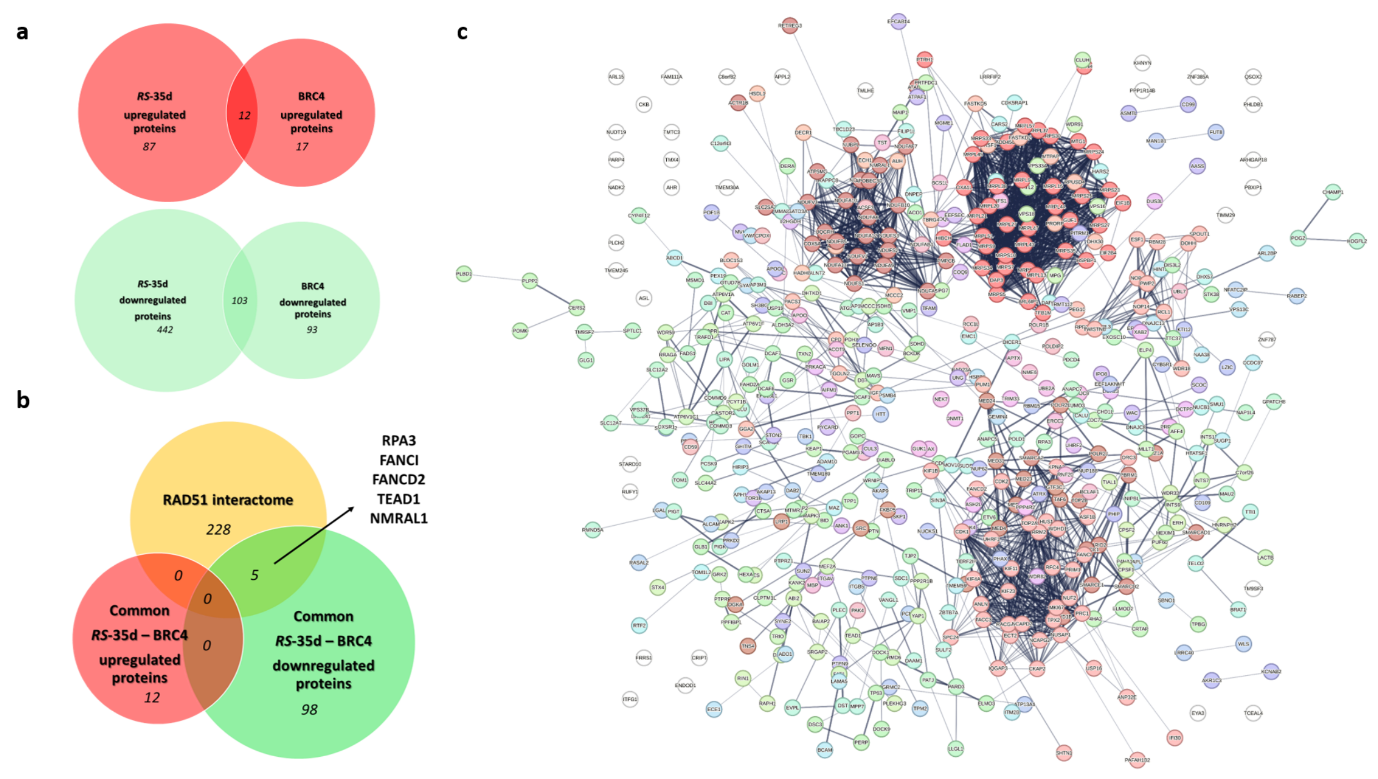


**Supplementary Fig. S13 | *RS*-35d proteomic profile in BxPC-3 cells.** (**a**) Venn diagram of common proteins up- and downregulated by *RS*-35d vs BRC4 treatment; (**b**) Venn diagram of commonly regulated proteins by *RS*-35d and BRC4 within RAD51 interactome. (**c**) STRING functional analysis of down-regulated proteins by *RS*-35d. Proteins were clustered using Markov clustering (MCL), with default inflation parameter = 3; the thickness of lines representing protein-protein interactions indicates the strength of data support.


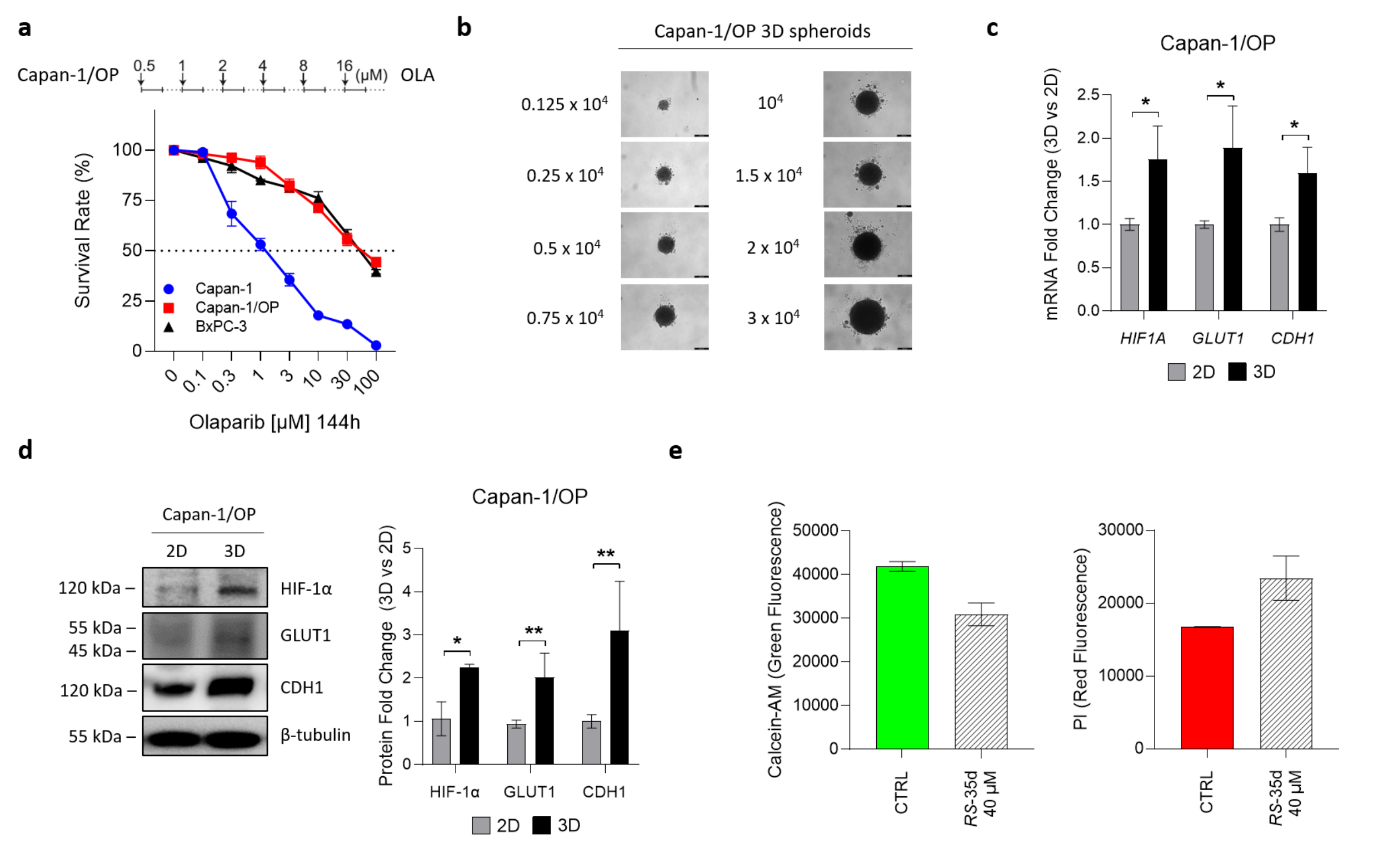


**Supplementary Fig. S14 | Establishment of olaparib-resistant Capan-1/OP cell line and characterisation of tumour-mimicking Capan-1/OP 3D spheroids.** (**a**) 6 months of exposure of Capan-1 cells to the PARP inhibitor olaparib (OLA) for the establishment of the resulting resistant variant Capan-1/OP; cell viability on Capan-1, Capan-1/OP and BxPC-3 cells exposed to increasing OLA concentrations for 144 h. (**b**) Representative images of 3D spheroids obtained at different Capan-1/OP cell concentrations (scale bar, 200 μm). The 3·10^4^ cells/well concentration was used for all the following experiments. **c**,**d** Capan-1/OP 3D spheroids (3D) characterisation via Real-Time PCR (**c**) and Western blot (**d**) to assess their correct PDAC tumour-mimicking properties compared to 2D cultures (2D). (**c**) *HIF1A*, *GLUT1* and *CDH1* mRNA expression in 3D vs 2D Capan-1/OP cells. *GAPDH* was used as endogenous reference control. (**d**) Representative Western blot images of HIF-1α, GLUT1 and CDH1 in 3D vs 2D Capan-1/OP cells; densitometric analysis of the corresponding protein bands. Results are normalised over β-tubulin expression and expressed as mean ± SD of three independent replicates. Statistical analysis was performed with one-way ANOVA followed by Tuckey’s multiple comparison test, with **p* < 0.05, ***p* < 0.01 or ****p* < 0.001 vs the respective 2D value. (**e**) Analysis of Calcein-AM (green fluorescence) and PI (red fluorescence) signals in *RS*-35d-treated Capan-1/OP 3D spheroids at 72 h. Measured values were used to calculate PI/Calcein-AM ratio to determine cell death rate.


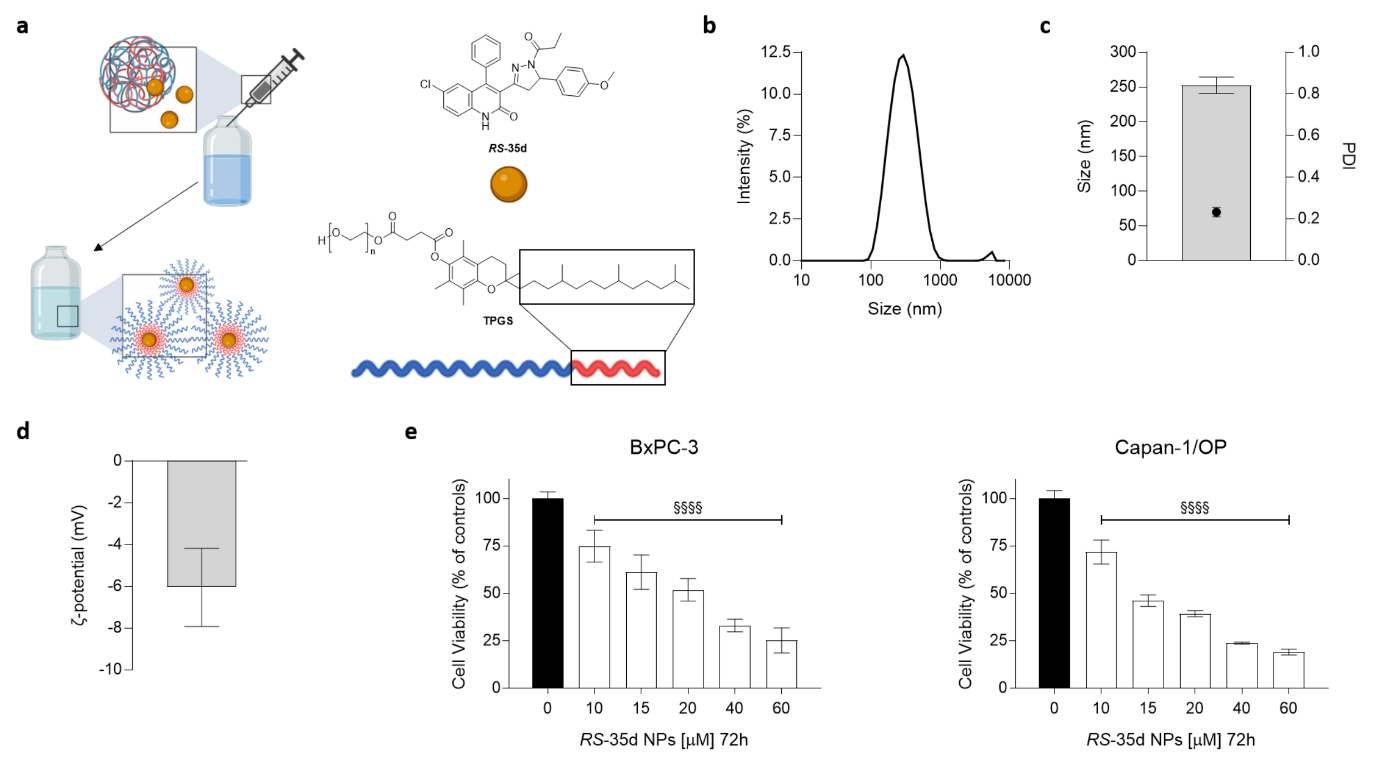


**Supplementary Fig. S15 | *RS*-35d NPs characterisation and effect on *BRCA2*-proficient and *BRCA2*-mutated, PARPi resistant PDAC cells.** (**a**) Diagram of *RS*-35d NPs structure and batch preparation; **b-d** DLS characterisation of *RS*-35d NPs for nanoparticle size (nm) (**b**), polydispersity index (PDI) (**c**) and ζ-potential (mV) (**d**). (**e**) Cell viability after 72 h treatment at different concentrations (10-60 µM) *RS*-35d NPs on BxPC-3 (*BRCA2*-proficient) and Capan-1/OP (*BRCA2*-mutated, PARPi resistant) cells. Results are expressed as mean ± SD of at least three independent experiments. Statistical analysis was performed with two-way ANOVA, followed by Tuckey’s multiple comparison test, with ^§§§§^p < 0.0001 vs CTRL (vehicle).
