# Supplementary material for: Investigating synthetic lethality and PARP inhibitor resistance in pancreatic cancer through enantiomer differential activity
Source: Cell Death Discov. 2025 Mar 16;11:106. doi: 10.1038/s41420-025-02382-3 (PMC11911456; doi:10.1038/s41420-025-02382-3)
Supplement: Supplementary file 2 — Supporting Information—Uncropped Immunoblot Images [file 41420_2025_2382_MOESM2_ESM.pdf]

Investigating Synthetic Lethality and PARP Inhibitor Resistance in Pancreatic Cancer Through Enantiomer Differential Activity

Uncropped Gels

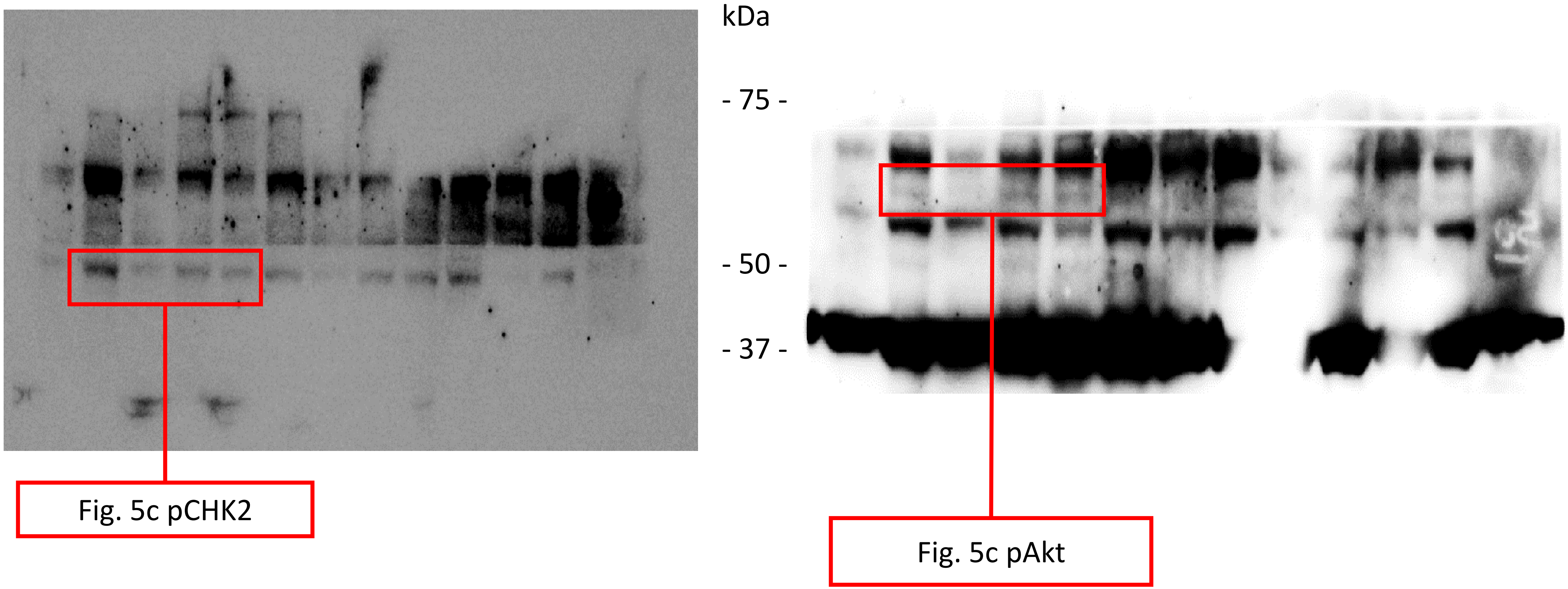
kDa
- 75 -
- 50 -
- 37 -
Fig. 5c pCHK2
Fig. 5c pAkt

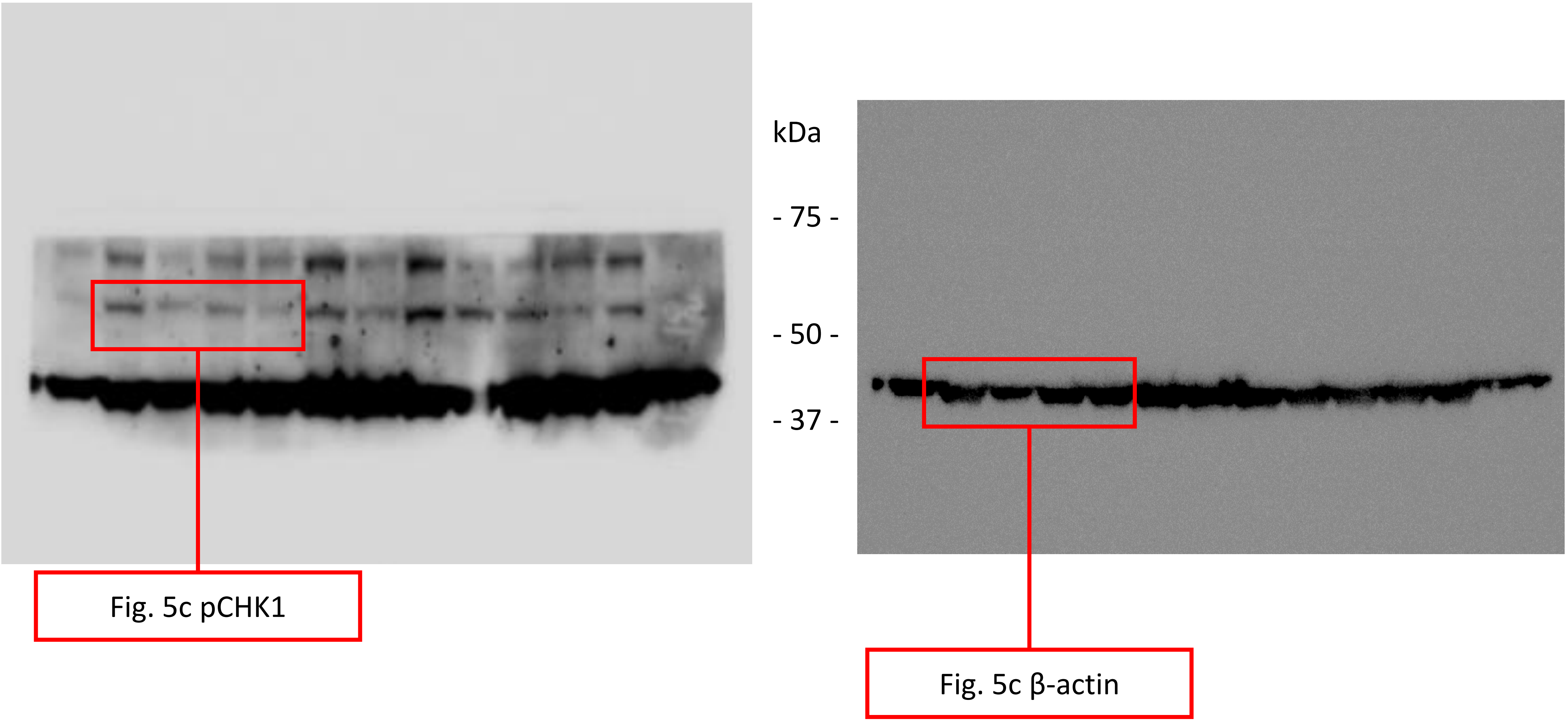

kDa
- 75 -
- 50 -
- 37 -
Fig. 5c pCHK1
Fig. 5c β-actin

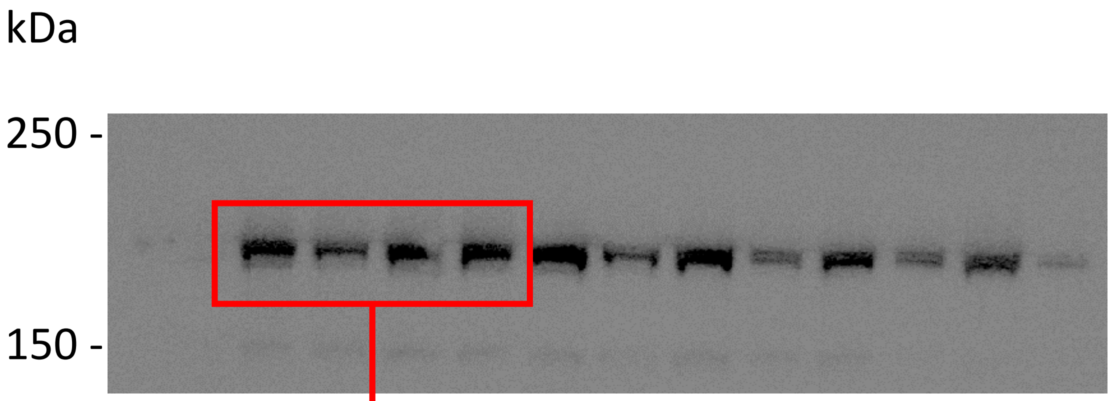

Fig. 6b FANCD2

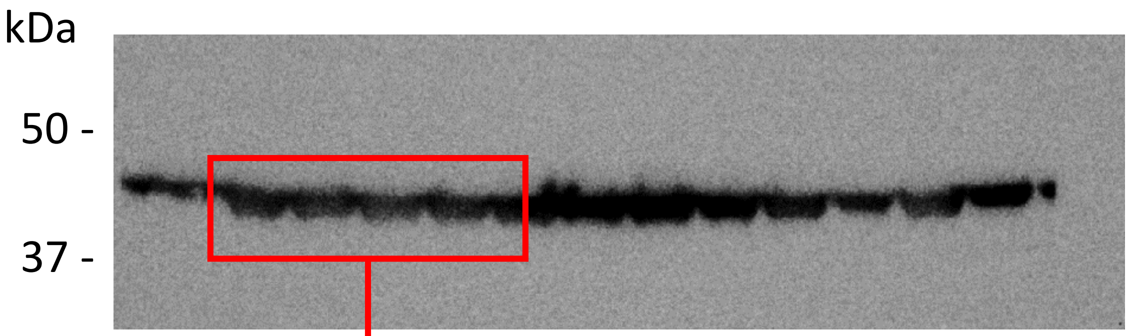

Fig. 6b β-actin

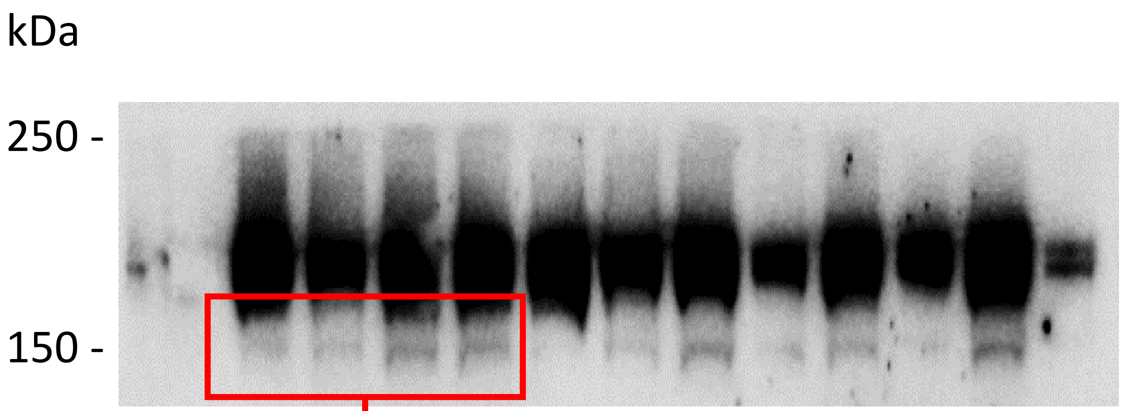

Fig. 6b FANCI

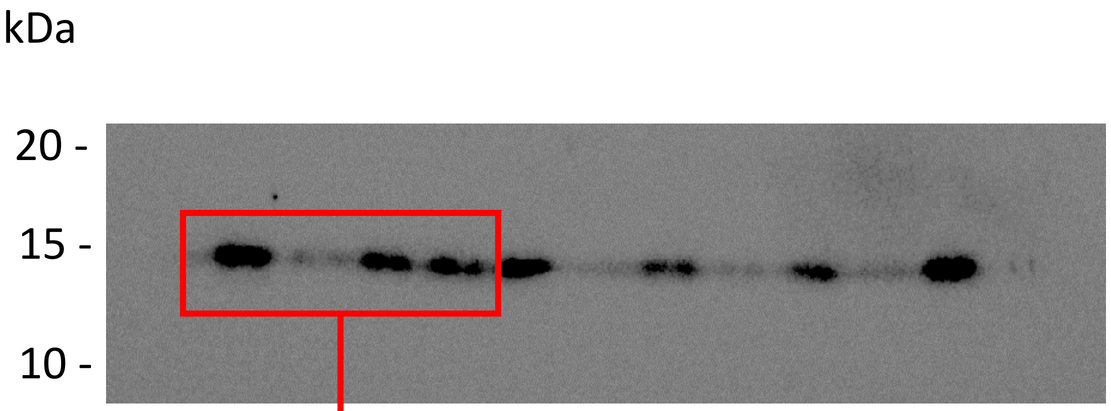

Fig. 6b RPA3
